# Balancing safety and efficiency in large-scale HRC: A dynamic scheduling method confronting temporal and spatial uncertainty of human workers

**DOI:** 10.1016/j.isci.2026.117095

**Published:** 2026-08-07

**Authors:** Houxue Xia, Zhenyu Sun, Huagang Tong

**Affiliations:** 1 School of Economics and Management, Nanjing Tech University, Nanjing 211816, China; 2 College of Management and Economics, Tianjin University, Tianjin 300072, China

**Keywords:** human-robot collaboration, human uncertainty, dynamic scheduling, deep reinforcement learning

## Abstract

In large-scale human-robot collaboration (HRC) workshops, humans exhibit dual uncertainty: temporal uncertainty in task completion times and spatial uncertainty in limb movements. These intertwined uncertainties create a fundamental tension between safety and efficiency. We propose a dynamic scheduling framework addressing both dimensions. For spatial uncertainty, a Perception-Prediction-Decision (PPD) framework is constructed, integrating Age-of-Information-based link resource allocation, digital twin elastic deployment, and probabilistic occupancy grid-based collision prediction to proactively maintain safety. For temporal uncertainty, an Improved Deep Q-Network (IDQN) algorithm is proposed, featuring an idle-duration-based executor priority mechanism, constraint-based action masking, and dynamic priority experience replay. Across 15 test scenarios, the PPD framework reduces collision response triggers by 59.9% while decreasing average Makespan by 12.1%; IDQN achieves only 2.5% performance degradation under high-volatility conditions, significantly outperforming DQN (6.0%), GA (9.7%), and rule-based methods (8.4%).

## Highlights

• A scheduling framework balances efficiency and human safety in large-scale HRC
• Age-of-Information-based prediction and proactive response improve human safety
• Deep reinforcement learning tackles uncertain human task durations for efficiency

## Graphical abstract

**Figure:**
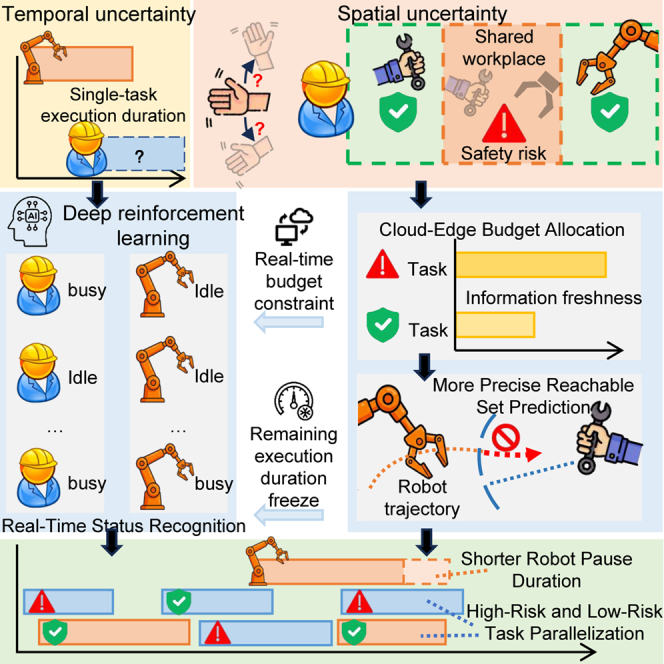

## Teaser

Computer science; Decision science; Industrial engineering

## Introduction

As industry evolves from Industry 4.0 toward Industry 5.0, human-centric manufacturing has become a central research theme in human-robot collaboration (HRC).^1^ Unlike traditional physically separated human-machine paradigms, HRC in the final assembly phase emphasizes close-range interaction and complementary strengths between humans and robots, improving production flexibility and efficiency while posing unprecedented challenges to personnel safety.^2^ Human workers exhibit dual uncertainty during task execution: spatial uncertainty—limb trajectories during task execution are inherently unpredictable; and temporal uncertainty—task completion times are difficult to predict accurately. Spatial uncertainty is the key factor affecting personnel safety, while temporal uncertainty is the key factor affecting scheduling efficiency.

According to statistics from the International Federation of Robotics, human-robot collision incidents have shown an upward trend alongside the expanding deployment of collaborative robots.^3^ In workshop environments widely deploying edge computing networks, real-time limb collision prediction for tens to nearly one hundred human executors incurs enormous instantaneous computational demands.^4^ Existing research indicates that edge computing environments face challenges including latency and bandwidth limitations and limited computational resources, making it difficult to meet the requirements of real-time control and rapid decision-making in large-scale manufacturing environments,^5^ with growing tension between high-bandwidth, low-latency data transmission requirements and limited wireless communication resources.^6^ From a systems perspective, HRC personnel safety assurance can be decomposed into three key stages: state perception, collision prediction, and obstacle avoidance decision-making. These three stages exhibit intrinsic coupling: delays in state perception degrade collision prediction accuracy; inaccurate prediction misjudges the timing of avoidance decisions; and response failures directly threaten personnel safety. Yet existing research has not addressed the intrinsic coupling among these three stages to systematically reduce HRC limb collision risk. Moreover, the inherent unpredictability of human task execution speed in large-scale multi-workstation settings creates dynamic scheduling problems that static or rule-based scheduling methods cannot reliably handle, and existing research rarely addresses the temporal and spatial uncertainty dimensions of human workers jointly within a unified framework.

HRC personnel safety research has evolved from passive response mechanisms (triggering obstacle avoidance only after a collision or near-miss is detected) to proactive collision-prediction-based avoidance. Samo et al.^7^^,^^8^ proposed a predictive velocity-space trajectory deconfliction method using dynamic safety ellipsoids, providing proactive obstacle avoidance capability in shared workspaces. Liu et al.^9^ proposed a graph-based robotic arm motion planner integrating uncertainty-aware human motion prediction, demonstrating the importance of uncertainty quantification for collision avoidance in shared environments. Zheng et al.^10^ proposed an encoder-decoder network-based human motion prediction method for collision avoidance in shared workspaces, providing robots with an advance avoidance time window. Faroni et al.^11^ proposed a safety-aware time-optimal motion planning method that accounts for uncertainty in human state estimation, establishing a forward reachable set model of human limbs through reachability analysis to predict the future possible positions of human limbs. The above studies have achieved good results in small-scale HRC scenarios with one or a few workstations, and also provide literature support for the collision prediction method proposed in this paper. However, in large-scale settings, the instantaneous computational cost of real-time limb collision prediction is enormous. All prior studies are built on the assumption that the upstream state information required for collision prediction arrives without transmission delay, without considering how the quality of upstream state information affects prediction accuracy. If edge network deployment costs are to be controlled, when state information arrives with delay, stale observations systematically degrade the reliability of collision prediction. Therefore, there is an urgent need for an HRC scheduling method that simultaneously accommodates large-scale settings and maintains limb collision prediction accuracy, so as to reduce the enormous costs incurred by edge computing consumption.

In recent years, digital twin (DT) technology has been increasingly applied to HRC safety scheduling. DTs maintain virtual mappings of physical entities, enabling real-time status monitoring and predictive analysis. In the HRC context, the DT serves as the core computational substrate for aggregating executor state information, computing human-robot limb distances, and assessing collision risk. Mao et al.^4^ and Cao et al.^5^ provided comprehensive analyses of edge computing architectures, demonstrating that the deployment location of the computing module (cloud vs. edge) fundamentally determines the end-to-end latency of state information, which directly affects the timeliness of safety monitoring in the HRC context. In this context, the last-hop latency from DT to executor is not a secondary engineering concern, but the primary determinant of whether the system can detect nascent collision risks in a timely manner. A growing body of research has validated the effectiveness of DTs as a platform for HRC safety monitoring. Wang et al.^12^ proposed a deep learning-enhanced DT framework for human-robot collaborative manufacturing, in which the DT generates synthetic training data and supports real-time recognition of human-robot collaborative behavior to ensure safety; experiments on both synthetic and real datasets demonstrated that the framework can accurately detect unsafe states and support autonomous intervention decisions. Choi et al.^13^ proposed a safety-aware HRC integrated mixed-reality system using deep learning and DTs, in which the DT is used to measure in real time the distance between the robot and the human operator in virtual space, providing a practical implementation of DT-driven collision risk quantification directly analogous to the approach adopted in the decision layer of this paper. Zhang et al.^14^ and Li et al.^15^ further demonstrated the applicability of DT technology in HRC manufacturing environments, showing that DTs can serve as an effective platform for coordinating safety monitoring and task execution. However, all of the above studies share a critical limitation: The DT deployment strategy is treated as a static configuration determined independently of the task scheduling decisions being made. This decoupling overlooks a bidirectional constraint relationship in large-scale HRC systems. On one hand, the high cost of edge deployment means that limited edge computing capacity constrains the number of high-risk executors whose DTs can be edge-deployed simultaneously, which in turn constrains the scheduling system’s freedom to simultaneously assign high-safety-risk collaborative tasks. On the other hand, task assignment results dynamically change which executors are executing high-safety-risk collaborative tasks at any given moment, thereby determining whose DTs need edge deployment to ensure low-latency safety monitoring. Deploying all executors’ DTs to the cloud leads to high latency in safety monitoring. Deploying all DTs uniformly to the edge incurs extremely high computational costs, and under the constraint of limited edge computing capacity, this will very likely rapidly exhaust edge resources, severely restricting the freedom to assign high-safety-risk collaborative tasks subsequently, thereby creating the risk of task delays.

Deep reinforcement learning (DRL) has emerged as a promising paradigm for dynamic manufacturing scheduling due to its ability to make real-time task assignment decisions without requiring complete prior knowledge of the production environment. Workneh and Gmira^16^ demonstrated the application of Deep Q-Networks (DQNs) to dynamic job shop scheduling, showing that RL-based methods outperform static optimization approaches under stochastic processing times by enabling online adaptation to actual system states. Qin et al.^17^ proposed a multi-agent dueling DQN method for dynamic production scheduling in large-scale personalized scenarios, achieving adaptive task allocation among heterogeneous agents by decomposing Q-values into state value and advantage components. These studies establish the foundational role of DRL in dynamic manufacturing scheduling and show that online learning significantly outperforms static planning under execution time uncertainty—a property directly relevant to the HRC scheduling challenge addressed in this paper. Constraint-based action masking has been demonstrated to be an effective technique for eliminating infeasible actions from an agent’s exploration space in constrained scheduling problems. Hou et al.^18^ conducted a systematic comparative study of invalid action masking across both on-policy and off-policy reinforcement learning algorithms, demonstrating that masking infeasible actions prior to Q-value evaluation consistently improves sample efficiency and convergence stability: By preventing agents from wasting training samples on permanently infeasible state-action pairs, action masking focuses the agent’s learning capacity on the feasible subspace of the decision problem. In the HRC task scheduling problem studied in this paper, feasibility constraints are particularly complex—simultaneously encompassing task precedence dependencies, executor load limits, edge resource capacity, and collision safety conditions—making action masking not only a convergence accelerator but also a structural necessity for generating valid scheduling decisions. The scheduling literature provides very limited guidance on the executor-level decision sequencing problem that arises in multi-executor HRC scenarios: When multiple executors become idle simultaneously, the order in which the agent assigns tasks to them affects the distribution of idle time across the executor pool, and consequently the overall makespan. Existing DRL schedulers, including those reviewed above, treat all simultaneously idle agents equivalently, or assign tasks in an arbitrary fixed order, lacking a principled mechanism for prioritizing which idle executor should receive a task assignment first. Priority experience replay based on temporal-difference (TD) error has been established as an effective mechanism for improving DRL sample efficiency. Li et al.^19^ proposed a dynamic priority adjustment framework in which the sampling priority of each stored experience is jointly determined by its TD error and the current training stage, demonstrating that this adaptive mechanism accelerates Q-network convergence and avoids the overfitting risk of statically prioritizing high-TD-error samples throughout training. The core insight—that experiences containing the largest discrepancy between the agent’s current Q-value estimate and the true return carry the greatest learning signal and should be preferentially replayed—provides a principled basis for the dynamic priority experience replay mechanism incorporated in the Improved Deep Q-Network (IDQN) proposed in this paper, which extends this idea by dynamically adapting the priority threshold to the current mean priority level of the experience pool.

To this end, this paper proposes a dynamic task scheduling method for large-scale HRC in the final assembly phase, oriented toward human dual uncertainty. To address spatial uncertainty, a systematic Perception-Prediction-Decision (PPD) optimization framework is constructed, coordinating the three stages of safety assurance through cross-layer joint optimization: the perception layer manages communication resource allocation based on Age of Information (AoI), minimizing transmission latency of critical safety state information; the prediction layer co-optimizes DT deployment strategies and task allocation under edge resource constraints, further reducing state information transmission latency between DTs and executors; the decision layer applies collision prediction based on probabilistic occupancy grids, triggering proactive robot action pauses at the nascent stage of collision risk. To address temporal uncertainty, an IDQN scheduling algorithm is proposed, incorporating constraint-based action masking, idle-duration-based executor decision priority, and dynamic priority experience replay to achieve real-time robust scheduling under the assumption that human task execution speeds are unpredictable.

The core contributions of this paper are as follows.

(1) A systematic PPD optimization framework oriented toward HRC safety is proposed. Compared to baseline methods, the collision response trigger count is reduced by 59.9% (from 44.6 to 17.9 times); meanwhile, the average Makespan is reduced by 12.1%.
(2) At the perception layer, a differentiated link resource allocation model based on AoI is proposed. The average AoI of high-priority executors is reduced by 65.7% (from 0.178 s to 0.061 s); collision response trigger count decreases from 24.5 to 16.1 times, and average Makespan is reduced by 8.0%.
(3) At the prediction layer, a co-optimization strategy for DT elastic deployment and task scheduling under resource constraints is proposed. The average total latency of high-priority executors is reduced by 71.0% (from 234.86 ms to 68.17 ms); compared to static cloud deployment, Makespan is reduced by 10.3%; compared to undifferentiated edge deployment, Makespan is reduced by 14.2% while maintaining comparable safety levels.
(4) An IDQN scheduling algorithm is proposed. Convergence episodes are reduced by 27.7% compared to standard DQN (5,907 vs. 8,174 episodes); IDQN’s performance degradation rate (PDR) is only 2.5% under high-volatility scenarios, significantly lower than standard DQN (6.0%), genetic algorithm (GA) (9.7%), and rule-based scheduling (8.4%).

**Figure 1:**
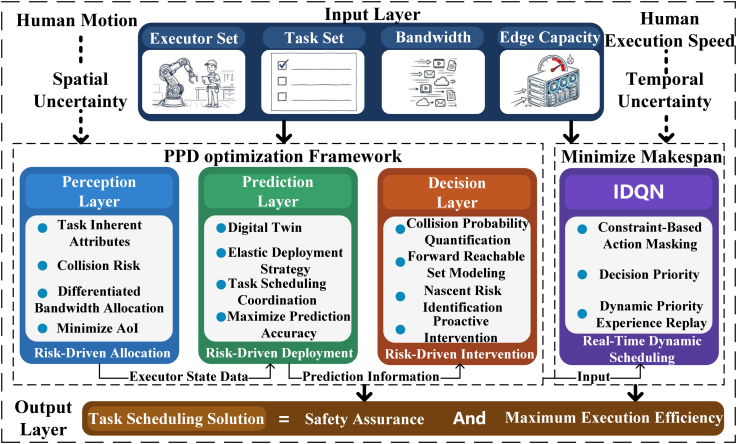
Schematic diagram of research content The figure illustrates the overall structure of the proposed dynamic scheduling framework for large-scale human-robot collaboration under temporal and spatial uncertainty. The framework integrates the perception layer, prediction layer, and decision layer. The perception layer improves the freshness of executor state information through age-of-information-based differentiated bandwidth allocation. The prediction layer supports low-latency collision prediction through digital twin elastic deployment. The decision layer combines probabilistic occupancy-based collision response with the IDQN scheduling algorithm to balance personnel safety and production efficiency.

The research content of this paper is shown in Figure 1.

## Results

### Experimental parameter settings

The experiments in this paper are based on an Intel CORE ULTRA 9 CPU, NVIDIA RTX 5070 GPU, 64 GB memory (hardware), and Python 3.10 (software). To comprehensively validate the effectiveness of the proposed method, a standardized test set containing 85 independent scenarios is constructed to simulate automotive assembly HRC workshops, of which 70 scenarios are used for policy learning of the IDQN agent, and 15 scenarios are used for final performance evaluation.

The 70 training scenarios are designed to cover the full range of scale and structural parameters specified in Table S1, ensuring that the IDQN agent encounters diverse task graph topologies, executor counts, and shared workspace proportions during training. The 15 held-out test scenarios are drawn from the same parameter range but are not used in training, providing an unbiased performance evaluation. All 85 scenarios are generated using fixed random seeds for their structural parameters (task graph topology, executor count, task type proportions), ensuring that the scenario set is reproducible. Within each training episode and each test run, human execution times are independently resampled from *U*(30, 60), so that although the scenario structure is fixed, the learning environment remains stochastic across episodes. Neural network weights are initialized with a fixed global seed, and training uses the Adam optimizer with a learning rate of 0.0001, as shown in Table S1. The standard DQN baseline shares the same Dueling DQN network architecture and state/action space as IDQN, with all hyperparameters in Table S1 identical, differing only in the absence of dynamic priority replay and constraint-based action masking.

The scale parameters of test scenarios are set as follows: number of tasks |J|∈[20,50], number of HRC workstations *N* ∈ [3, 12], number of AMRs *K* ∈ [2, 7], total number of executors |E|=2N+K∈[8,31]. Task type proportions are robot-only tasks (RO) accounting for 20%–30%, human-only tasks (HO) accounting for 20%–30%, human-robot flexible tasks (HRF) accounting for 40%–60%, with shared workspace tasks accounting for 20%–60%. The task precedence graph is generated using a random layer-by-layer construction procedure that guarantees acyclicity by design. The total task set is partitioned into *L* layers, where *L* is sampled uniformly from {2, 3, 4, 5, 6} for each scenario. Tasks are distributed across layers in approximately equal numbers. For each task in layer *k* + 1 (*k* ≥ 1), one or two predecessor tasks are selected uniformly at random from all tasks in layer *k*, with the predecessor count drawn from {1, 2} with equal probability. This construction guarantees the acyclicity of valid DAGs by design, since all edges strictly point from lower layers to higher layers. The resulting graph has an average out-degree of approximately 1.5 tasks per layer, producing task dependency structures representative of multi-stage automotive assembly processes.

Robot execution time $T_{j}^{R}∼U\left(20,40\right)$ is a deterministic value, fixed at task generation; human execution time ${\tilde{T}}_{j}^{H}∼U\left(30,60\right)$ is a random variable, independently sampled each episode, and can only be known at task completion time. This design realistically simulates the uncertainty of human execution time, forcing the scheduling algorithm to learn robust dynamic strategies. To simulate the spatial behavior of human limbs in the shared workspace during task execution in the simulation, the following trajectory generation mechanism is adopted: when a human executor begins executing a shared workspace task, the limb departs from the executor’s workstation boundary, enters the center of the shared workspace with acceleration not exceeding *a*_*max*_, and then returns along a symmetric path. Throughout the entire trajectory, limb velocity follows U(*v*_*min*_,*v*_*max*_) = U(0.1, 0.8) m/s, where *v*_*min*_ = 0.1 m/s represents the low-speed fine manipulation phase during actual assembly operations, and *v*_*max*_ = 0.8 m/s represents the higher-speed entry and exit phases. The total time of the complete trajectory (entry, operation, exit) is sampled from U(30, 60) s, consistent with the scheduling-layer parameter $T_{j}^{H}$. At each simulation time step, the current limb position *p*_*t*_ and velocity *v*_*t*_ are updated according to this trajectory model and transmitted to the DT with an AoI delay of Δ seconds, yielding the input values *p*_*obs*_ = *p*(*t*-Δ) and *v*_*obs*_ = *v*(*t*-Δ) for the forward reachable set computation described. The robot end-effector follows a deterministic three-phase trajectory during each shared-workspace task execution. At scenario generation, one waypoint is randomly sampled within the shared workspace region, and two waypoints are randomly sampled within the robot-exclusive workspace region; the end-effector traverses these three waypoints in sequence along a cubic spline interpolated path and then returns along the same path, with total motion duration $T_{j}^{R}$; the instantaneous speed *v*_*R*_(*t*) at each simulation time step is determined by the trajectory geometry and $T_{j}^{R}$. Each robot trajectory is fixed at scenario generation and remains unchanged across all independent runs. When a safety stop is triggered, the end-effector position and $T_{e}^{remain}\left(t\right)$ freeze instantaneously; when the stop condition clears, the robot immediately resumes from its frozen position at its original speed along the remaining trajectory.

The task-level temporal parameter $T_{j}^{H}$ and the kinematic-level motion prediction parameters are intentionally decoupled in the hierarchical design of the PPD framework: $T_{j}^{H}$ governs the duration of human presence in the shared workspace (determining the exposure time window for collision risk), while the motion prediction parameters govern the instantaneous collision risk at each moment within that window. This decoupling reflects the architectural separation between the IDQN scheduling layer (which handles temporal uncertainty through real-time observation of $T_{e}^{remain}\left(t\right)$) and the PPD safety layer (which handles spatial uncertainty through forward reachable set computation), allowing each layer to be independently parameterized and validated.

### IDQN achieves scalable and robust scheduling across 15 scenarios

The IDQN agent is trained for 10000 episodes using 70 training scenarios. Figure S1 shows the cumulative reward curve during the training process. The training process exhibits three typical phases: In the exploration phase (episodes 0–2,000), the cumulative reward fluctuates dramatically, with a mean of approximately −813; in the rapid learning phase (episodes 2,000–6,000), the reward improves from −813 to −497; in the stable convergence phase (episodes 6,000–10,000), the reward stabilizes and ultimately converges to approximately −317. The training process validates the convergence of the IDQN algorithm under complex constraint conditions.

A medium-scale scenario T5 (30 assembly subtasks, 5 HRC groups, 3 AMRs, 13 executors in total, 7 shared workspace tasks) is selected from the 15 test scenarios to demonstrate the dynamic scheduling results of IDQN. The predecessor dependency chains contained in the task DAG of this scenario include *J*_12_→*J*_25_, *J*_28_→*J*_15_→*J*_6_, *J*_17_→*J*_23_. Figure S2 shows the task scheduling Gantt chart for this scenario.

As shown in Figure S2, the final Makespan is 230 s. H0, H1, and H2 each complete 3 tasks; R0 and R1 each sequentially handle 4 tasks, reflecting a relatively balanced load distribution among the executors. The 6 shared workspace tasks (J7, J13, J18, J21, J22, J26, highlighted with red borders) are distributed among human and robot executors, and all tasks with predecessor dependencies strictly follow the execution order constraints, indicating that IDQN maintains acceptable scheduling efficiency while guaranteeing constraint satisfaction through constraint-based action masking.

To validate the scheduling performance of the IDQN algorithm under different workshop scales, the IDQN agent is run on each of the 15 test scenarios, with each scenario independently run 20 times to eliminate the influence of randomness in human execution time. Table 1 shows the scheduling result statistics for each test scenario. As the number of tasks increases from 20 to 50 and the number of executors increases from 8 to 31, the average Makespan grows from 251.6 s to 349.1 s, an increase of approximately 38.8%. Benefiting from the growth in executor count as scenario scale expands, the Makespan growth rate is significantly lower than the task count growth rate (150%). This indicates that the IDQN algorithm can effectively exploit the parallel potential of executors to offset the effect of increased task volume, maintaining good scheduling efficiency as problem scale grows. The ratio of Makespan standard deviation to mean across scenarios remains stable in the range of 1.3%–10.3%, indicating good algorithmic robustness against the stochasticity of human execution time. This pattern holds consistently from small-scale to large-scale scenarios, indicating that the observed volatility originates from the inherent stochasticity of human execution times rather than from instability in the algorithm itself. The collision response count is positively correlated with the proportion of shared workspace tasks; the average safety pause time accounts for 2.4%–9.5% of the Makespan, indicating that the probabilistic occupancy-based collision prediction mechanism can improve personnel safety at a relatively small efficiency cost. In summary, the experimental results validate the effectiveness of the proposed IDQN dynamic task scheduling algorithm, demonstrating good scalability and robustness.

**Table 1:** IDQN scheduling results for 15 test scenarios

Scenario	Number of tasks	Number of executors	State dimension |***s***_***t***_|	Shared workspace task proportion	Average Makespan (s)	Standard deviation (s)	Average collision response count	Average robot safety pause time (s)
T1	20	8	175	32%	251.6	23.5	3.3	7.8
T2	22	9	193	41%	254.2	12.0	5.1	11.0
T3	25	10	218	35%	282.8	16.1	2.8	6.8
T4	28	12	243	48%	318.8	7.0	7.7	16.6
T5	30	13	263	23%	229.4	6.4	4.1	9.8
T6	32	15	283	52%	257.2	11.8	8.2	17.4
T7	35	17	311	25%	230.4	8.6	3.9	7.6
T8	37	19	331	39%	286.2	3.6	7.1	15.8
T9	39	21	351	42%	293.4	15.7	8.7	15.2
T10	40	23	367	21%	257.3	6.1	7.1	11.1
T11	42	25	391	42%	279.4	17.1	7.1	16.0
T12	44	27	415	58%	301.6	31.2	10.0	28.2
T13	46	29	439	22%	275.1	19.8	4.9	12.4
T14	48	30	451	29%	332.2	4.5	4.1	8.6
T15	50	31	477	60%	349.1	6.3	14.4	33.2

### Differentiated AoI-based bandwidth allocation reduces collision triggers

First, we validate whether the proposed differentiated bandwidth allocation strategy based on collision risk level can effectively reduce the AoI of high-risk task executors, thereby improving the timeliness of safety monitoring. Scenario T6 (32 tasks, 15 executors, shared workspace task proportion 52%) from the test set is selected for comparative experiments. The experimental group adopts the differentiated bandwidth allocation strategy based on collision risk level; the control group adopts an undifferentiated bandwidth allocation strategy. Each group of experiments is independently run 20 times, and statistics are collected for the average AoI of high-priority executors (humans executing shared workspace tasks), average AoI of low-priority executors, average collision response count, average robot safety pause time, and average Makespan as shown in Table S2.

As shown in Table S2, the differentiated bandwidth allocation strategy achieves significant improvements across all safety-critical metrics. The average AoI of high-priority executors is reduced by 65.7% (from 0.178 s to 0.061 s) compared to the uniform allocation control group, enabling substantially more timely collision risk monitoring. The average collision response count decreases from 24.5 to 16.1 (a reduction of 34.3%), and the average robot safety pause time is reduced by 44.7% (from 32.0 s to 17.7 s), both indicating that more accurate and timely state information suppresses false collision triggers. Consequently, average Makespan decreases from 279.9s to 257.4s, a reduction of 8.0%. Although the low-priority executor AoI in the experimental group (0.317s) is higher than that of the control group (0.178s), since low-priority executors perform exclusive workspace tasks with no collision risk, the elevated AoI does not compromise safety. This reflects the core design philosophy of “safety-critical information priority transmission” embedded in the proposed method.

**Figure 2:**
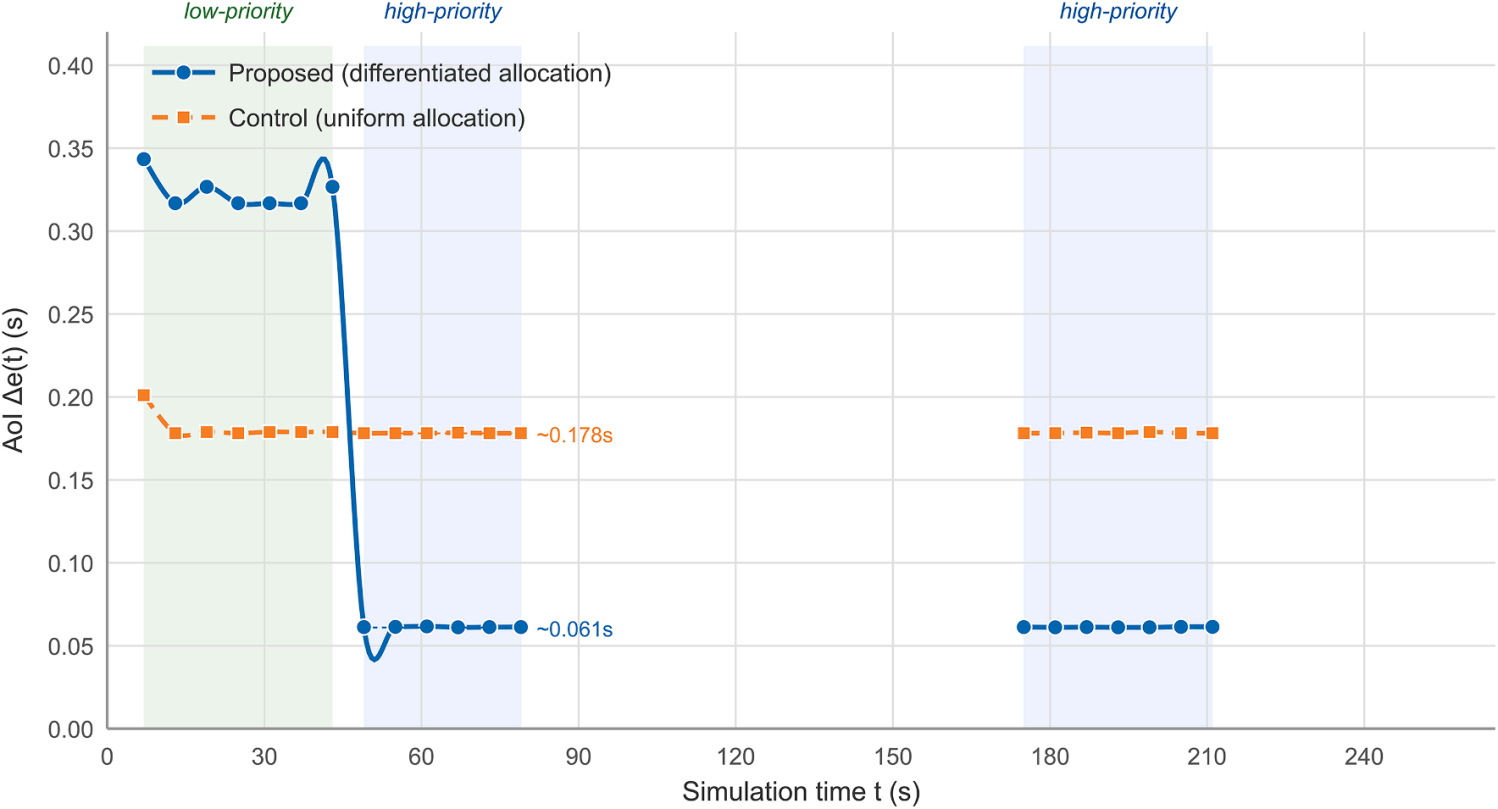
Differentiated bandwidth allocation reduces high-priority AoI by 65.7% upon entry into shared workspace AoI time series for executor H3 from a representative single run of scenario T6. The solid line represents the experimental group with differentiated bandwidth allocation, and the dashed line represents the control group with uniform bandwidth allocation. Shaded regions indicate task execution phases, including one low-priority exclusive-workspace phase and two high-priority shared-workspace phases. AoI is zero during idle periods because communication resources are not allocated to idle executors by design. Aggregate statistics are calculated across *n* = 20 independent runs.

Figure 2 presents the AoI time series of executor H3 across two shared workspace task execution phases (*t* = 49–79 s and *t* = 175–211 s) and one low-priority phase (*t* = 7–43 s). During the low-priority phase, H3 executes an exclusive workspace task and is allocated a lower bandwidth quota in the experimental group; the resulting AoI levels differ only marginally from those of the control group (approximately 0.32s in the experimental group versus approximately 0.20s in the control group), which is consistent with the design intent. During the high-priority phases, H3 transitions to executing shared workspace tasks, and the differentiated allocation strategy prioritizes increasing H3’s bandwidth, reducing its AoI to approximately 0.061s—a 65.7% reduction relative to the control group (approximately 0.178s). The control group maintains consistently high AoI throughout all phases in which H3 is executing tasks, reflecting the inability of the uniform allocation strategy to respond dynamically to changes in collision risk level. To conserve link communication resources as much as possible, no communication resources are allocated by default to any executor in the idle state; consequently, AoI is zero during idle periods in the simulation experiments. These results confirm that the differentiated allocation strategy effectively concentrates link resources at the moment executors enter high-risk shared zones, thereby improving the information freshness of collision prediction inputs.

Comparative experiments are conducted under the same experimental conditions across three scales (small, medium, and large), with results reported in Table S3. The results in Table S3 show that the performance gains of differentiated bandwidth allocation hold consistently across all three scales and exhibit a systematic trend that is positively correlated with the shared workspace task proportion. The magnitude of high-priority AoI reduction remains highly stable across the three scenarios (T3: 66.4%, T6: 65.7%, T12: 63.2%), indicating that the improvement in information freshness achieved by the differentiated allocation strategy is independent of workshop scale. The magnitude of collision trigger reduction increases monotonically with the shared workspace task proportion (T3: 21.6%, T6: 34.3%, T12: 35.4%): In larger workshops with higher shared workspace task proportions, bandwidth competition among high-priority executors is more intense, and the prioritization effect of the differentiated allocation strategy is therefore more pronounced. The magnitude of Makespan reduction follows the same monotonically increasing pattern (T3: 2.6%, T6: 8.0%, T12: 9.1%), which is highly consistent with the trend in collision trigger reduction, further corroborating the causal chain by which perception-layer AoI improvement propagates to scheduling efficiency through enhanced collision prediction accuracy. These results demonstrate that the AoI differentiated allocation mechanism possesses reliable generality within the operational range of the framework, and that its benefits systematically strengthen as workshop scale and task complexity increase.

### DT elastic deployment reduces high-priority latency and Makespan

Next, we validate the effectiveness of the proposed DT elastic deployment strategy. Scenario T9 (39 tasks, 21 executors) with intense edge resource competition is selected, with three groups set up for comparison: The experimental group adopts the elastic deployment strategy (high-priority deployed to edge, low-priority deployed to cloud), control group 1 uses static cloud deployment, and control group 2 uses undifferentiated edge deployment. Each group of experiments is independently run 20 times, and statistics are collected for average total latency of high-priority executors (including link latency + server latency), average total latency of low-priority executors, average task waiting time, collision response trigger count, average Makespan and other metrics, with experimental results shown in Table S4.

**Figure 3:**
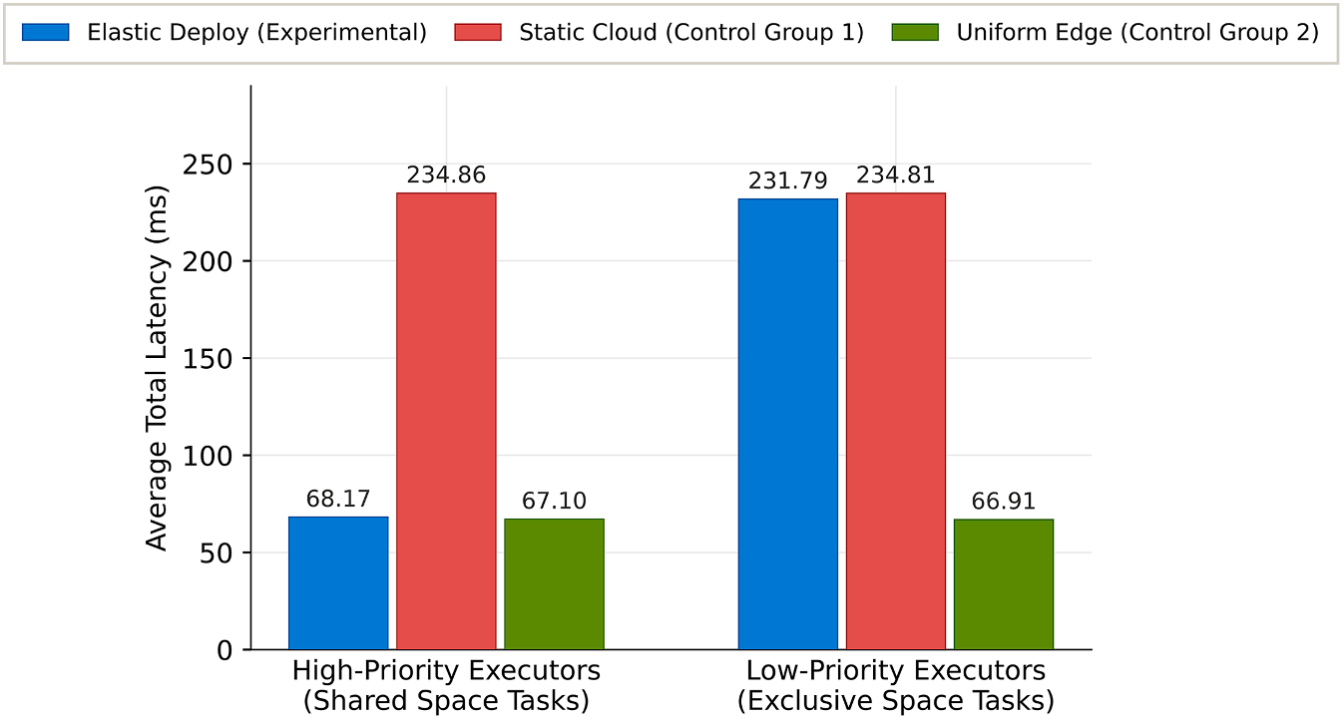
Elastic deployment achieves low high-priority latency without triggering edge resource overload Bar charts compare three digital twin deployment strategies in scenario T9 (39 tasks, 21 executors): elastic deployment, static cloud deployment, and undifferentiated edge deployment. The elastic deployment strategy assigns high-priority digital twins to edge resources while assigning low-priority digital twins to the cloud. Bars represent mean values across *n* = 20 independent runs. The results show that elastic deployment maintains low latency for high-priority executors while avoiding task delays caused by edge resource exhaustion.

As shown in Table S4, control group 2 achieves low latency for both high-priority and low-priority executors (67.10 ms and 66.91 ms, respectively), comparable to the experimental group’s high-priority latency (68.17 ms). However, this “undifferentiated edge deployment” strategy leads to rapid exhaustion of edge resources. Once edge server capacity is fully occupied, subsequent tasks must wait for existing tasks to complete and release resources before they can begin execution; this generates an average of 36.9 task delays caused by insufficient edge resources per experimental group run, resulting in an average task waiting time of 51.7 s–5.1 times that of the experimental group (10.1 s)—and an average Makespan of 341.8 s, representing a 16.5% increase relative to the experimental group. This demonstrates that blindly pursuing low latency while ignoring resource constraints actually damages overall scheduling efficiency. The experimental group achieves the dual objective of “low latency for high-priority executors and no edge resource overload” through the differentiated deployment strategy. Compared to control group 1, the experimental group achieves a 71.0% reduction in high-priority latency, a 51.4% reduction in collision response trigger count, a 56.6% reduction in average robot safety pause time, and a 10.3% reduction in Makespan. Compared to control group 2, the experimental group achieves a 14.2% reduction in Makespan while maintaining a comparable safety level. Figures 3 and S3 provide intuitive visual comparisons of the results for each metric. The experimental results demonstrate that the DT elastic deployment model, through its differentiated deployment strategy, simultaneously satisfies the low-latency requirement of high-risk task executors and avoids task delays caused by edge resource overload, achieving a strong balance between safety and production efficiency.

The results in Table S5 show that the DT elastic deployment strategy consistently achieves the expected benefits across all three scales, while exhibiting a scale-dependent pattern that reflects the underlying resource competition mechanism. The reduction in high-priority latency relative to static cloud deployment remains highly significant at all scales (78.4%, 71.0%, and 69.8% for T3, T9, and T12, respectively) and shows a slight narrowing trend as scale increases. This is consistent with the fact that edge resource contention becomes more intense in larger-scale scenarios, slightly increasing the probability of occasional deployment failures; nonetheless, the overall magnitude of reduction remains considerable, indicating that the proximity advantage of elastic edge deployment maintains stable consistency within the operational range of the framework. By contrast, the advantage relative to undifferentiated edge deployment increases substantially with scale: The number of task delays caused by edge resource exhaustion under the undifferentiated edge strategy grows from 27.6 in T3 to 36.9 in T9 and further to 43.8 in T12, whereas the elastic strategy maintains zero task delays across all three scales. This pattern directly validates the design rationale of the elastic deployment model: In small workshops with fewer executors, edge resource competition is mild and the two strategies perform similarly; as workshop scale and shared workspace task density increase, the ability of the differentiated deployment strategy to prevent edge resource overload becomes increasingly critical to scheduling efficiency.

### PPD three-layer coupling yields larger makespan reduction with superadditive gains

**Figure 4:**
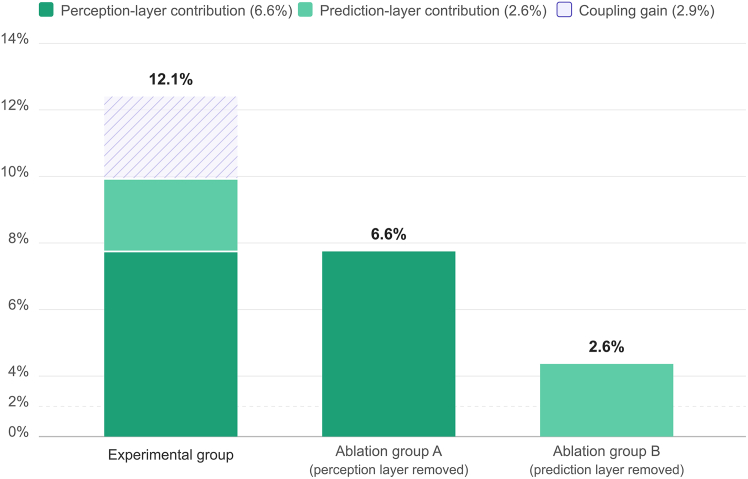
Cross-layer coupling contributes a 2.9-percentage-point superadditive gain beyond the sum of individual layer contributions The figure decomposes the Makespan improvement of the full PPD framework relative to the baseline. The full framework achieves a 12.1% Makespan reduction, whereas the independent contributions of the perception layer and prediction layer account for 6.6% and 2.6%, respectively. The remaining 2.9 percentage points indicate a positive cross-layer coupling gain. Values are averaged over all 15 test scenarios, with *n* = 20 independent runs per scenario. The coupling gain is computed as: total improvement − (perception contribution + prediction contribution).

The overall effectiveness of the PPD three-layer coupled optimization framework is validated through ablation experiments. All 15 scenarios from the test set are selected with 4 groups set up for comparison: experimental group (PPD three-layer coordinated optimization), ablation group A (remove AoI differentiated allocation, adopt equal bandwidth allocation, retain prediction layer and decision layer), ablation group B (remove DT elastic deployment, all DTs deployed to cloud, retain perception layer and decision layer), baseline group (retain only decision layer). Each configuration is independently run 20 times on each of the 15 test scenarios, and the high-priority executor average total latency, collision response trigger count (PPD soft-stop activation count), SSM hard-stop trigger count, SSM hard-stop total duration, and average Makespan are statistically recorded, with experimental results shown in Table 2. Among these, the SSM hard-stop metrics originate from the ISO/TS 15066-based hard safety constraint embedded in the simulation: at each simulation time step, both the “stale” human limb position based on information freshness and the actual human limb position are simultaneously computed, and SSM hard-stop events are recorded—along with their cumulative duration until recovery—based on the actual human-robot separation distance between the actual human limb position and the robot limb position at that moment. These two SSM metrics are completely independent of the PPD probabilistic layer and the IDQN policy, providing a deterministic physical-layer safety reference. Meanwhile, Figures 4 and S4 are plotted to more intuitively demonstrate the contribution of each layer’s optimization and the coupling effect. In Figure S4, scores are normalized: 1.0 represents the experimental group, and 0 represents the baseline group; a larger polygon area indicates better overall performance.

**Table 2:** Ablation experimental results of PPD coupled framework

Metric	Experimental group	Ablation group A	Ablation group B	Baseline group
High-priority total latency (ms)	67.1	137.2	282.2	408.9
Collision response trigger count	17.9	26.1	36.1	44.6
SSM hard-stop trigger count (times/run)	4.2	3.9	3.3	2.9
Total SSM hard-stop duration (s/run)	6.9	6.8	6.1	5.0
Average Makespan (s)	317.3	337.2	351.6	360.9

As shown in Figure S4 and Table 2, the average Makespan of the experimental group is reduced by 12.1% compared to the baseline group’s 360.9 s; the PPD-based collision response trigger count decreases from 44.6 to 17.9, a reduction of 59.9%. Notably, as shown in Figure 4, the sum of the Makespan improvement margins of ablation groups A and B (6.6% + 2.6% = 9.2%) is less than the improvement margin of the experimental group (12.1%), revealing a positive cross-layer coupling gain of 2.9 percentage points: The reduction in perception-layer link latency provides the prediction layer with fresher state data; the reduction in prediction-layer deployment latency provides the decision layer with more accurate collision predictions; the synergistic effect of all three causes the overall system performance to exceed the simple sum of each individual layer’s independent optimization. The experimental results demonstrate that the PPD framework, by establishing a cross-layer joint optimization model that precisely allocates limited resources to high-risk phases, performs well in the synergistic optimization of personnel safety improvement and scheduling efficiency improvement in large-scale HRC scenarios.

Furthermore, the two SSM-based metrics reveal an important interaction relationship between the two safety layers. As shown in Table 2, the SSM hard-stop trigger count and total duration exhibit a pattern opposite to that of the collision response trigger count: The experimental group has the highest SSM activity (4.2 times/run, 6.9 s/run), while the baseline group has the lowest (2.9 times/run, 5.0 s/run). This apparent reversal is a direct consequence of the substitution effect between the PPD soft-stop layer and the SSM hard-stop layer. In the experimental group, the low end-to-end latency (67.1 ms) produces a compact forward reachable set and accurate collision probability estimates, enabling the PPD soft-stop to intervene precisely and early—the robot pauses before the human limb approaches the SSM threshold distance *S*(*t*), thereby reducing the occasions on which the SSM is forced to act as a last resort; but at the same time, genuine collision risks are also identified and responded to earlier. In the baseline group, the high latency (408.9 ms) causes the reachable set to expand, leading to a large number of false triggers by the PPD (44.6 times/run); the robot is frequently and prematurely placed in a paused state by PPD soft-stops, which objectively maintains physical distance between the robot and the human body, and in turn reduces the occasions on which the SSM threshold is physically reached. In other words, the more frequently the PPD triggers, and the earlier the robot stops, the fewer genuine physical near-proximity situations actually occur. This substitution mechanism means that, across groups with different PPD activation frequencies, the SSM trigger count is not a monotonic indicator of the degree of safety improvement, but rather reflects the residual physical near-miss risk that the PPD soft layer failed to intercept. The higher SSM activity of the experimental group relative to the baseline group does not mean that the experimental group is physically less safe; rather, it means that the PPD’s excessive triggering in the baseline group incidentally suppressed physical near-proximity events as a side effect. Taken together, these four metrics jointly constitute a multi-level safety-efficiency evaluation system: The collision trigger count characterizes the intervention frequency of the PPD probabilistic mechanism; the SSM trigger count and hard-stop duration characterize the residual physical near-miss risk after PPD intervention; and Makespan captures the overall production efficiency cost of all safety interventions. These results simultaneously reveal the complementary relationship between the PPD framework and the SSM hard constraint: the PPD, by improving the timeliness of DT state perception, significantly reduces collision probability misjudgments based on inflated reachable sets, thereby reducing the frequency of unnecessary robot pauses and improving scheduling efficiency; however, the proactive prevention mechanism based on a probabilistic threshold is inherently unable to provide deterministic interception of all physical near-miss events, and residual risk remains when human limb motion exceeds the boundaries of model assumptions. The SSM hard constraint exists precisely as the ultimate safety net for this residual risk: It intervenes based on a deterministic geometric condition that is completely independent of the probabilistic model and scheduling policy, ensuring that physical contact can still be prevented when the PPD fails to respond in time. The two layers have clearly defined and complementary roles, and together they form the complete closed loop of the safety architecture proposed in this paper.

The performance advantage of the complete framework holds consistently across all 15 test scenarios: Within the full range of problem scales tested, the complete PPD configuration achieves the lowest Makespan in every scenario, confirming that the coupling gains among the three layers are robust to variation in task count, executor count, and shared workspace task proportion.

By tracing the quantitative causal chain from the perception layer through the prediction layer to the decision layer, the cross-layer interaction mechanism is made more explicit:

(1) The first link in the causal chain is the perception-layer contribution: the differentiated bandwidth allocation reduces AoI, thereby tightening the forward reachable set and suppressing excessive activation of collision triggers. The experimental data from Section “Differentiated AoI-based Bandwidth Allocation reduces Collision triggers” show that differentiated bandwidth allocation reduces the high-priority executor AoI from 0.178 s to 0.061 s (a reduction of 65.7%), decreases the collision response trigger count from 24.5 to 16.1 (a reduction of 34.3%), and reduces the average Makespan by 8.0%. In the ablation experiments (Table 2), this perception-layer contribution is further quantified: Removing the AoI differentiated allocation (ablation group A) increases high-priority total latency from 67.1 ms to 137.2 ms (+104.5%), raises the collision trigger count from 17.9 to 26.1 (+45.8%), and narrows the Makespan improvement over the baseline group from 12.1% to 6.6%. The underlying mechanism is as follows: equal bandwidth allocation reduces the link bandwidth allocated to high-priority executors, *D*_*link*_ increases accordingly, the effective AoI rises, the forward reachable set radius *r*_*reach*_(Δ) expands, the collision probability estimate is biased upward, and this leads to more frequent unnecessary robot pauses.
(2) The second link in the causal chain is the prediction-layer contribution: DT elastic deployment reduces the state transmission latency from the DT to the executor, further tightening the reachable set on top of the bandwidth allocation. The experimental data from section “DT elastic deployment reduces high-priority latency and Makespan” show that DT elastic deployment reduces the high-priority executor total latency from 234.86 ms to 68.17 ms (a reduction of 71.0%), decreases the collision response trigger count from 17.7 to 8.6 (a reduction of 51.4%), reduces the robot safety pause time from 37.1 s to 16.1 s (a reduction of 56.6%), and reduces the Makespan by 10.3%. In the ablation experiments (Table 2), removing DT elastic deployment (ablation group B) increases high-priority total latency from 67.1 ms to 282.2 ms (+320.6%), raises the collision trigger count from 17.9 to 36.1 (+101.7%), and narrows the Makespan improvement to 2.6%. Notably, the latency penalty incurred by removing the prediction layer (+215.1 ms) is 3.1 times that of removing the perception layer (+70.1 ms), indicating that DT elastic deployment quantitatively dominates the independent contributions of the two layers in terms of latency reduction and collision trigger suppression.
(3) The third link in the causal chain explains the 2.9-percentage-point coupling gain (12.1%–6.6% − 2.6%) that separates the complete PPD framework from the sum of the two individual layer contributions. This superadditive gain originates from the forward reachable set radius’s joint dependence on communication link latency (perception layer) and DT-to-executor deployment latency (prediction layer): since *r*_*reach*_(Δ) grows with effective AoI, and effective AoI is jointly determined by both latencies, the reduction in reachable set volume achieved by simultaneously compressing both latencies is greater than the sum of compressing each independently, thereby yielding greater collision trigger suppression and Makespan reduction. In other words, each layer’s optimization amplifies the effect of the other in a nonlinear manner rather than linearly stacking, and this is precisely the mechanism behind the coupling gain observed in Figure 4 and Table 2.

### IDQN outperforms baselines in scheduling performance and dynamic robustness

To validate the effectiveness and real-time feasibility of IDQN as a scheduling solver for handling human temporal uncertainty in HRC, three representative baseline algorithms are selected for comparison:

(1) Standard DQN: adopts the same Dueling DQN network architecture and state/action space as IDQN, but uses uniform random experience replay in place of dynamic priority replay and does not include constraint-based action masking; all other hyperparameters, including learning rate, batch size, replay buffer capacity, discount factor, and soft update coefficient, are identical to the IDQN specifications in Table S1, ensuring that the comparison isolates the effect of the two IDQN-specific improvements.
(2) GA: population size 100, real-valued chromosome encoding (each gene represents the assigned executor for one task), crossover rate 0.8 (single-point crossover), mutation rate 0.1 (random reassignment of a randomly selected gene to a different executor), iterated for 1,000 generations. The fitness function is the total Makespan of the pre-scheduled plan, computed by simulating task execution in topological order using the distribution upper bound of human execution time, 60 s (the maximum of U(30,60)), to generate a conservative pre-schedule before task execution begins; predecessor constraints are enforced during fitness evaluation by penalizing schedules that violate dependency ordering. The GA generates a static task-to-executor assignment before execution and does not reassign tasks during execution regardless of actual human execution times.
(3) Rule-based scheduling: at each decision step, idle executors are assigned tasks according to a composite priority score *P*(*j*,*e*) = *ω*_1_·*S*(*j*) + *ω*_2_·*I*(*e*) + *ω*_3_·*M*(*j*,*e*), where *S*(*j*) is the normalized task slack time (deadline minus earliest completion time), *I*(*e*) is the normalized cumulative idle duration of executor *e*, *M*(*j*,*e*) is the task-executor type match (1 if the executor type matches the task type, 0 otherwise), and *ω*_1_ = *ω*_2_ = *ω*_2_ = 1/3 (equal weights). When the edge resource capacity reaches its upper limit, the rule-based scheduler defers the assignment of shared-workspace tasks until edge capacity becomes available, handling resource constraints in the same feasibility-preserving manner as IDQN’s action masking.

**Figure 5:**
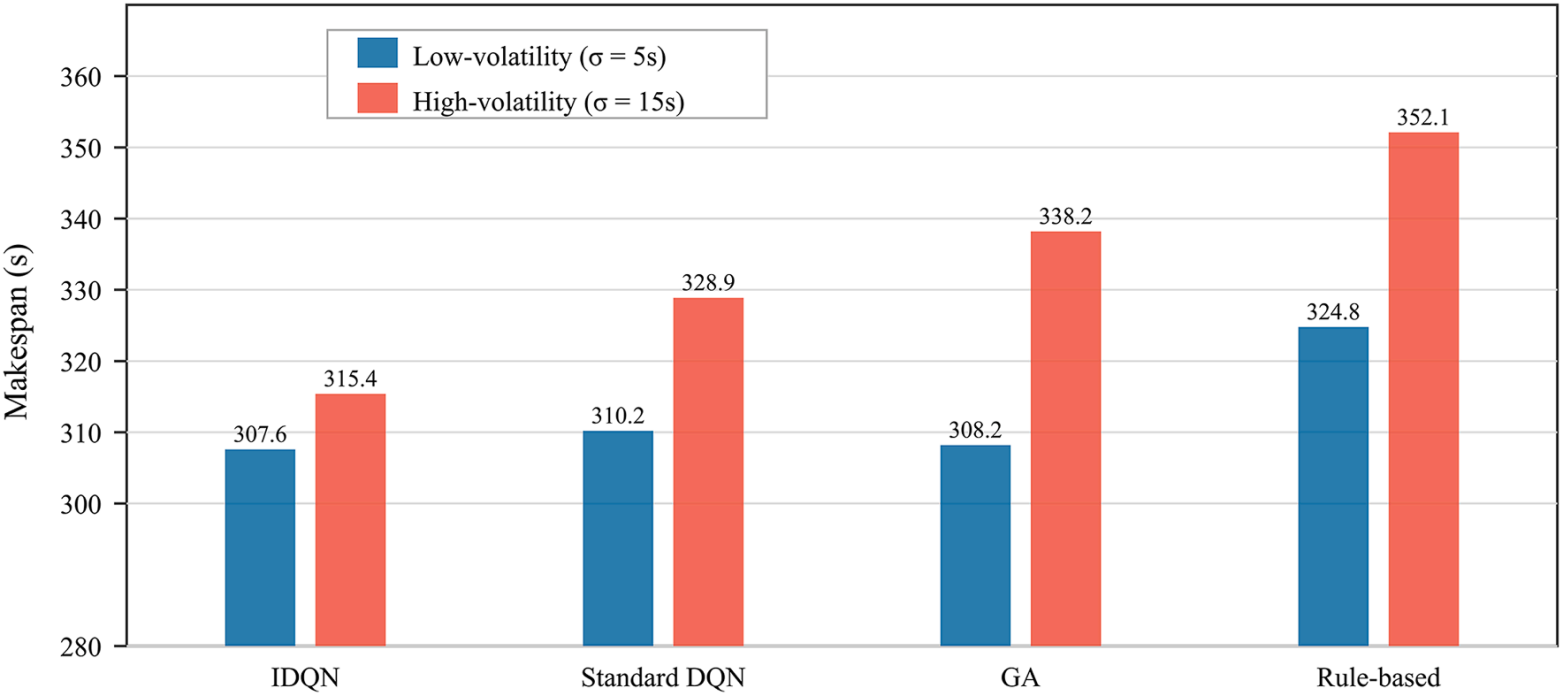
IDQN maintains the lowest performance degradation rate (2.5%) as human execution volatility increases Grouped bar charts compare the average Makespan of IDQN, standard DQN, genetic algorithm, and rule-based scheduling under low-volatility (σ = 5 s) and high-volatility (σ = 15 s) human execution time conditions. Bars represent mean values across all 15 test scenarios, with *n* = 20 independent runs per scenario. IDQN shows the smallest performance degradation rate, indicating stronger robustness to temporal uncertainty in human task execution.

Experiments are conducted on all 15 test scenarios, with each scenario independently run 20 times, comprehensively evaluated from four dimensions: convergence, scheduling performance, dynamic robustness, and computational efficiency (as shown in Table 3). The comprehensive performance comparison results of the four algorithms on the 15 test scenarios are presented. Figure S5 illustrates the training convergence curve comparison, and Figure 5 shows the Makespan comparison under different volatility levels to more intuitively demonstrate the performance differences of each algorithm.

**Table 3:** Comprehensive algorithm performance comparison

Evaluation dimension	Metric	IDQN	Standard DQN	GA	Rule-based
Convergence	convergence episodes^a^	5907	8174	–	–
	post-convergence reward standard deviation	17.9	32.1	–	–
Scheduling performance	average Makespan (s)	315.9	329.4	321.6	335.7
	makespan standard deviation (s)	17.8	24.6	61.9	63.4
	optimal solution acquisition rate (%)^b^	74.1	40.9	63.5	17.1
Dynamic robustness	low-volatility scenario Makespan (s)^c^	307.6	310.2	308.3	324.8
	high-volatility scenario Makespan (s)^d^	315.4	328.9	338.2	352.1
	performance degradation rate (%)^e^	2.5	6.0	9.7	8.4
Computational efficiency	average single decision time (ms), T1 (E = 8)	19.7	18.9	–	1.4
	average single decision time (ms), T8 (E = 19)	24.5	24.3	–	1.5
	average single decision time (ms), T15 (E = 31)	27.3	26.8	–	1.5
	max single decision time (ms), T15	32.8	31.6	–	2.7
	real-time constraint satisfied (≤1000 ms)	yes (all 15 scenarios)	yes (all 15 scenarios)	–	yes (all 15 scenarios)

The reported value is averaged across all 15 test scenarios.

a Convergence episodes refer to the training episode at which the cumulative reward first reaches a stable state.

b The optimal solution acquisition rate refers to, for each test scenario, the proportion of runs out of 20 independent runs in which the algorithm’s Makespan falls within 10% of the best Makespan achieved by that algorithm across its own 20 runs for that scenario.

c The low-volatility scenario refers to a human execution time standard deviation of σ = 5 s.

d The high-volatility scenario refers to σ = 15 s.

e The performance degradation rate is calculated as (high-volatility Makespan—low-volatility Makespan)/low-volatility Makespan.

As shown in Figures 5 and S5 and Table 3, IDQN’s convergence episodes are reduced by 27.7% compared to standard DQN (5,907 vs. 8,174 episodes), with smaller reward fluctuations after convergence (post-convergence reward standard deviation: 17.9 vs. 32.1). This benefits from the dynamic priority experience replay mechanism’s ability to adaptively focus on high-value experiences, and the constraint-based action masking’s avoidance of invalid action exploration. Regarding scheduling performance, IDQN achieves the lowest average Makespan (315.9s) among all four algorithms, outperforming GA by 1.8% (321.6s), standard DQN by 4.1% (329.4s), and rule-based by 5.9% (335.7s). Although GA’s Makespan standard deviation (61.9s) is 3.5× higher than IDQN’s (17.8s), indicating high output variance, IDQN maintains both a lower mean and a lower variance simultaneously. IDQN also achieves the highest optimal solution acquisition rate (74.1%) among all algorithms—10.6 percentage points above GA (63.5%) and 33.2 percentage points above DQN (40.9%)—demonstrating IDQN’s superior ability to consistently find high-quality scheduling solutions. When human execution time volatility increases from low to high, IDQN’s PDR (2.5%) is significantly lower than all comparison algorithms (DQN: 6.0%, rule-based: 8.4%, GA: 9.7%). GA, as a static pre-scheduling algorithm, cannot adapt to dynamic changes during execution, resulting in the highest degradation rate. Rule-based, although capable of real-time decision-making, its fixed priority rules are difficult to handle complex uncertainty scenarios. IDQN, through online learning and real-time decision-making, dynamically adjusts scheduling strategies based on current system states, effectively absorbing temporal uncertainty in human execution. Regarding computational efficiency, IDQN’s single decision time satisfies the real-time constraint (≤1,000 ms) across all 15 test scenarios. Although rule-based is fastest (1.4 ms at T1), its scheduling performance is significantly inferior. IDQN achieves a good balance between scheduling performance and computational efficiency. In summary, IDQN demonstrates competitive performance across all four evaluation dimensions. In particular, under high-uncertainty dynamic scenarios, its PDR (2.5%) is lower than that of all competing algorithms (DQN: 6.0%, rule-based: 8.4%, GA: 9.7%), confirming that IDQN is competent as a robust and real-time dynamic scheduling solver in HRC environments subject to temporal uncertainty.

To directly demonstrate the real-time feasibility of the IDQN model at maximum scale, we report per-scenario inference time measurements across scenarios T1–T15. As shown in Table 3, the average single-decision time increases from 19.7 ms at T1 (E = 8) to 27.3 ms at T15 (E = 31), with a maximum single-decision time of 32.8 ms observed at T15. As the number of executors increases by 287.5% (from 8 to 31), the average inference time increases by only 38.6%, confirming the sub-linear growth consistent with the complexity analysis derived in Section “Complexity Analysis of State Space and Scalability Discussion.” Critically, even the worst-case maximum decision time of 32.8 ms at T15 remains 96.7% below the real-time constraint $T_{dec}^{\max}=1$ s, providing a substantial margin for deployment on resource-constrained edge computing platforms. These results confirm that the IDQN algorithm maintains practical real-time feasibility across the full range of tested scales.

### IDQN achieves competitive scheduling performance with lower decision latency

To further examine the performance of IDQN, we compare IDQN against QMIX^20^ and MAPPO,^21^ both adapted to the centralized HRC scheduling setting of this paper. To ensure fairness of comparison, QMIX and MAPPO are both adapted to the centralized HRC scheduling setting of this paper as follows:

(1) For QMIX, each executor is treated as an independent agent; each agent receives the same globally structured state vector as IDQN as input, and the local Q-network adopts a fully connected network with two hidden layers of 256 neurons each; the mixing network uses a single hidden layer of 64 neurons to implement monotonic mixing, satisfying QMIX’s monotonicity constraint.
(2) For MAPPO, both the Actor network and the Critic network adopt a fully connected structure with two hidden layers of 256 neurons each; the Critic receives the global state as input, while the Actor receives the same structured state vector as IDQN and outputs the action probability distribution for each executor.

All three algorithms apply the same constraint-based action masking to ensure feasibility of scheduling decisions, and are trained for the same total number of steps (10,000 steps) on the same set of training scenarios, with the Adam optimizer and a unified learning rate of 1 × 10^−4^. All three algorithms are independently run 20 times on all 15 test scenarios, and the results are summarized in Table S6.

As shown in Table S6, the three algorithms exhibit distinctly differentiated performance across the three dimensions of scheduling performance, dynamic robustness, and computational efficiency. In terms of scheduling performance, MAPPO, leveraging the more thorough exploration of the policy space afforded by policy gradient methods, achieves the lowest average Makespan among the three (313.7 s), slightly outperforming IDQN (315.9 s, a gap of 0.7%) and QMIX (321.4 s), owing to the richer policy space exploration characteristic of policy gradient approaches. However, the Makespan standard deviations of MAPPO and QMIX (26.9 s and 32.1 s, respectively) are higher than that of IDQN (17.8 s), being 1.51 and 1.80 times that of IDQN, respectively, indicating that while the richer policy exploration space of frontier MARL methods can yield relatively higher-quality solutions, it also poses challenges to the stability of obtaining high-quality solutions during cross-scenario training. This is further corroborated by the optimal solution acquisition rate, where IDQN (74.1%) outperforms both QMIX (61.8%) and MAPPO (62.4%). In terms of dynamic robustness, when human execution time volatility increases from low volatility (σ = 5 s) to high volatility (σ = 15 s), IDQN’s PDR is 2.5%, which, compared to 2.2% and 2.0% for QMIX and MAPPO, respectively, is equally acceptable. This stems from the dynamic priority experience replay mechanism, which, by adaptively focusing on high-value experiences, enables IDQN to effectively absorb the stochastic fluctuations in human execution time even in real HRC environments with high uncertainty. As shown in Figure S6, in terms of computational efficiency, IDQN’s single decision time increases from 19.7 ms at T1 (E = 8) to 27.3 ms at T15 (E = 31), with a maximum decision time of 32.8 ms at T15, fully satisfying the real-time constraint (≤1000 ms) with a substantial margin. By contrast, QMIX achieves an average decision time of 101.2 ms (maximum 118.6 ms) and MAPPO 88.3 ms (maximum 109.2 ms) at T15, which are 3.7 and 3.2 times that of IDQN, respectively. IDQN’s decision speed advantage originates from the idle-duration-based sequential priority ordering mechanism, which decomposes the joint action space into *E* sequential decisions, thereby circumventing the combinatorial joint action computation required by MARL methods (QMIX requires an additional mixing network forward pass, while MAPPO requires forward passes through both Actor and Critic networks, making both slower than the single-network IDQN).

It is worth noting that the architectural advantages of QMIX and MAPPO are subject to structural constraints under the problem formulation of this paper. QMIX’s value decomposition framework can provide principled cross-agent credit assignment in genuinely decentralized multi-agent problems, but the HRC scheduling in this paper is essentially a centralized single-agent assignment problem, where the theoretical advantages of value decomposition cannot be leveraged, and the additional mixing network instead introduces training complexity, preventing QMIX’s advantages from being fully realized. MAPPO’s policy gradient approach does demonstrate more thorough policy space exploration under low-uncertainty conditions, but requires forward passes through both Actor and Critic networks, leaving room for further optimization in computational efficiency. How to integrate QMIX’s value decomposition or MAPPO’s actor-critic advantage with IDQN’s HRC-specific constraint handling mechanism is a future research direction worth exploring.

### Action masking dominates training efficiency while decision priority drives dynamic robustness

To quantify the independent contributions of the executor decision priority mechanism, the constraint-based action masking mechanism, and the DT-based candidate-action forward simulation mechanism to IDQN’s scheduling performance, five groups of ablation experiments are conducted. All groups retain the dynamic priority experience replay mechanism to ensure consistent experience sampling quality and differ only in the combination of the two mechanisms under study. The ablation group configurations are: Group A (full IDQN, serving as the comparison baseline in this experiment), Group B (decision priority removed, using random ordering), Group C (action masking removed, allowing the full action space), Group D (DT forward simulation removed, agent makes action decisions based on Q-values only), and Group E (standard DQN with all three mechanisms removed). All 15 test scenarios are used, with 20 independent runs per scenario. The results are summarized in Figure S7 and Table S7.

The ablation results reveal the differentiated functional roles of the three mechanisms. In the convergence efficiency dimension, removing action masking (Group C, 7,681 episodes) has the most pronounced impact on training efficiency, representing a 30.0% increase relative to the full IDQN (Group A, 5,914 episodes); removing DT forward simulation (Group D, 6,717 episodes) ranks second with a 13.6% increase; removing decision priority (Group B, 6,432 episodes) has the smallest impact with an 8.8% increase. This gradient clearly reflects the distinct operating levels of the three mechanisms: action masking directly trims infeasible actions to compress the effective exploration space, and its effect on training efficiency is the most fundamental; without masking, the agent must rely purely on the reward signal to exclude infeasible assignments, substantially increasing the number of samples required for effective exploration; DT forward simulation operates at the inference stage rather than at the level of training space structure, and thus has a moderate effect; decision priority primarily regulates the balance of resource allocation within an already effectively explored space, and has limited impact on the structure of the exploration space itself.

In the scheduling performance dimension, the Makespan improvements contributed by each of the three mechanisms are as follows: the decision priority mechanism (Group A vs. Group B) contributes 13.2 s, the action masking mechanism (Group A vs. Group C) contributes 16.1 s, and the DT forward simulation mechanism (Group A vs. Group D) contributes 6.1 s. The sum of the individual contributions of decision priority and action masking (29.3 s) exceeds the improvement of the full IDQN relative to the reference baseline with both removed, indicating a complementary positive interaction between the two: The ordered decision sequence provides supplementary support to the action masking mechanism by further eliminating infeasible actions caused by resource constraints and similar factors for lower-priority decisions, thereby further compressing the effective exploration space and enabling the IDQN agent to further accelerate its learning efficiency.

The optimal solution acquisition rate most clearly characterizes the different contributory nature of the three mechanisms. Removing decision priority (Group B, 52.1%) and removing action masking (Group C, 44.8%) both lead to a substantial decline in the optimal solution rate, falling by 27.2 percentage points and 34.5 percentage points respectively relative to the full IDQN (79.3%), indicating that both are critical supports for the policy’s stable pursuit of optimal solutions across scenarios. The impact of removing DT forward simulation (Group D, 67.8%) is comparatively concentrated in the refinement stage of “pushing good solutions toward optimal solutions:” Group D’s average Makespan degradation is only 1.9%, whereas its optimal solution acquisition rate drops by 11.5 percentage points, because the core value of DT forward simulation lies in incorporating collision risk into decision-making through candidate-action forward prediction to refine the safety-efficiency tradeoff, rather than altering the coarse-grained structure of scheduling.

In the dynamic robustness dimension, removing decision priority (Group B, 5.4%) yields the highest PDR among all ablation groups, substantially exceeding that of the full IDQN (3.1%), indicating that this mechanism is the primary contributor to enhancing the policy’s dynamic robustness. The fundamental reason is that the decision priority mechanism, by guiding the agent to respond preferentially to executors with the longest waiting times, continuously maintains load balance among executors. When human execution times exhibit high volatility, a load-balanced executor scheduling regime provides greater absorption capacity for fluctuations, thereby suppressing the sensitivity of Makespan to temporal uncertainty. By contrast, removing action masking (Group C, 3.3%) and removing DT forward simulation (Group D, 3.7%) both yield degradation rates close to that of the full IDQN, indicating that neither mechanism is a decisive factor affecting the algorithm’s dynamic robustness—action masking primarily contributes to training efficiency and policy feasibility, while DT forward simulation primarily contributes to decision refinement. Overall, the three mechanisms form a functionally complementary hierarchical structure: Action masking establishes the feasibility foundation of the policy and determines learning efficiency; the decision priority mechanism ensures the balanced allocation of executor resource utilization; and DT forward simulation provides fine-grained calibration of the safety-efficiency tradeoff. The three working in concert enable the full IDQN to outperform the standard DQN baseline (Group E) across all three dimensions of average Makespan, optimal solution acquisition rate, and dynamic robustness.

### IDQN maintains the lowest performance degradation under skill-fatigue coupled heterogeneity

In the preceding sections, IDQN has been demonstrated to be an effective HRC dynamic scheduling solver, exhibiting a low PDR under the temporal uncertainty benchmark setting (U(30,60)). However, the above conclusions are all built upon a homogeneous assumption: The execution times of all human executors are independently and identically sampled from the same distribution, meaning all workers share the same skill level and are unaffected by within-shift accumulated fatigue. Yet the workforce in real-world manufacturing shops deviates systematically from this assumption. Jaber et al.^22^ explicitly identified workers’ skill level and fatigue state as coupled factors jointly affecting task execution efficiency, and mathematically demonstrated the central property of this coupling mechanism within the learning-forgetting-fatigue-recovery model (LFFRM) for manufacturing environments: Skill accumulation (learning) can systematically reduce the rate of fatigue accumulation. Liu et al.,^23^ in studying the hybrid flow shop scheduling problem that incorporates both multi-skilled workers and fatigue factors, likewise incorporated worker fatigue and skill level simultaneously into the modeling framework for hybrid flow shops based on this coupling mechanism, establishing an agent-based simulation system to address uncertainty in worker fatigue models. This implies that while humans exhibit heterogeneity in task execution, they also embody the regularity that “skilled workers not only execute faster, but also experience a slower rate of speed decline as the number of tasks increases.”

Based on the above analysis, the core focus of the experimental design in this section is to validate the robustness of the IDQN policy itself to heterogeneous human behavior that lies outside the training distribution: specifically, to verify whether the IDQN policy trained under the homogeneous distribution assumption can maintain stable scheduling performance under heterogeneous labor force conditions. To characterize the coupled effect of skill and fatigue in simulation, this section introduces a task execution time generation model based on human heterogeneity. When worker *e* executes their *k*-th task within the current shift, the execution time is sampled from *T*_*j*,*e*,*k*_∼*U*(*t*_*min*_/(*s*_*e*_·*f*_*e*,*k*_),*t*_*max*_/(*s*_*e*_·*f*_*e*,*k*_)), where the capacity retention coefficient *f*_*e*,*k*_ is given by $f_{e,k}=\max\left(f_{\min},1-\alpha_{e}\cdot k\right),\alpha_{e}=\alpha_{0}\cdot {s_{e}}^{-\gamma }$, where *s*_*e*_ ∈ {0.75,1.00,1.30} is the worker skill coefficient, corresponding to novice, ordinary, and skilled workers, respectively; *α*_*e*_ is the fatigue decay rate of worker *e*, characterized by the power-law function $\alpha_{e}=\alpha_{0}\cdot {s_{e}}^{-\gamma }$, where *α*_0_=0.05 is the reference fatigue rate for ordinary workers, and *γ*=1.5 is the exponent representing the strength of skill’s suppression of fatigue; *f*_*min*_=0.50 is the lower bound of capacity retention; *k* is the number of tasks the worker has completed in the current episode. The rationale for introducing the power-law function $\alpha_{e}=\alpha_{0}\cdot {s_{e}}^{-\gamma }$ derives from the conclusion of Jaber et al.^22^ within the LFFRM framework: Skill accumulation reduces the rate of fatigue accumulation in a nonlinear manner, and the power-law form captures the law of diminishing marginal returns of skill improvement. This setting fixes the parameter values for the three worker types as: novice (*s*_*e*_ = 0.75, *α*_*e*_≈0.077), ordinary worker (*s*_*e*_ = 1.00, *α*_*e*_≈0.050), and skilled worker (*s*_*e*_ = 1.30, *α*_*e*_≈0.033), with the three worker types distributed in equal proportions at each HRC workstation.

The model involves two key couplings: first, *s*_*e*_ simultaneously affects the “starting speed” (higher skill means a faster baseline) by modulating the upper and lower bounds of the distribution support; second, *α*_*e*_ decreases monotonically with *s*_*e*_, causing skilled workers to accumulate within-shift fatigue significantly more slowly than novices, thereby exhibiting a widening performance advantage over novices at the *k*-th task—this is precisely the essence of the coupling between fatigue and skill proficiency, rather than their independence. Compared to the homogeneous baseline (all workers independently and identically sampled from U(30,60), this model simultaneously introduces between-worker variation (heterogeneity in *s*_*e*_) and within-worker temporal drift (the decline of *f*_*e*,*k*_ as *k* increases), constituting a more realistically challenging robustness testing scenario. It should be noted that skill proficiency and fatigue level are set as “parameters known prior to task assignment” in the simulation. Therefore, in the simulation, the upper and lower bounds of human execution times based on heterogeneity are set as quantities known prior to the task assignment decision.

The experiment defines three human behavior settings and evaluates IDQN and three comparison baselines under each setting:

(1) Group A (Homogeneous Baseline): All workers’ execution times are independently and identically sampled from U(30,60), fully consistent with the training distribution, serving as the reference baseline.
(2) Group B (Skill Heterogeneity Only): Workers are assigned to three skill types, with execution times drawn from *U*(*t*_*min*_/*s*_*e*_,*t*_*max*_/*s*_*e*_), introducing only the between-worker variation in *s*_*e*_ without the fatigue drift that increases with the number of tasks (*f*_*e*,*k*_≡1).
(3) Group C (Skill-Fatigue Coupling): The full coupled model described above is adopted, simultaneously introducing skill heterogeneity and fatigue decay.

The experimental focus of this section is to longitudinally track the Makespan degradation trajectory of each algorithm across the three settings, in order to evaluate its robustness to heterogeneous labor force conditions. The comparison baselines comprise three representative algorithm types: standard DQN (a learning-based online method), GA (a pre-scheduling static method), and rule-based scheduling (a heuristic online method), to compare the differences in degradation magnitude across scheduling paradigms under heterogeneity pressure.

**Figure 6:**
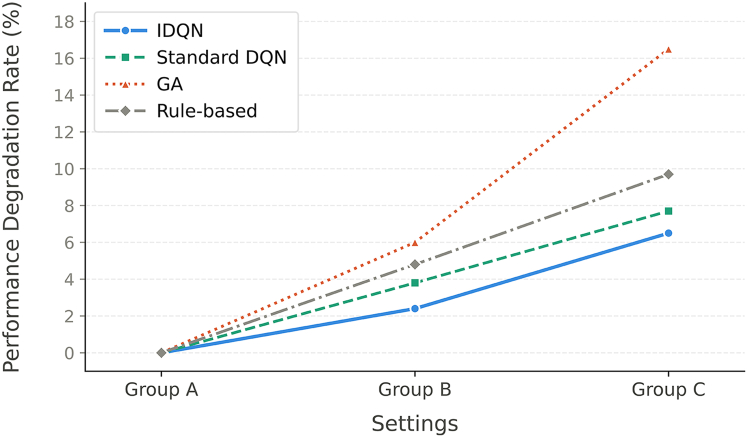
IDQN shows the flattest PDR trajectory under skill-fatigue coupling, confirming superior robustness to heterogeneous labor conditions The figure compares the performance degradation rate trajectories of IDQN, standard DQN, genetic algorithm, and rule-based scheduling under different labor-force heterogeneity settings. PDR = 0 for all algorithms in Group A (homogeneous baseline). The slope from Group B to Group C reflects each algorithm’s sensitivity to the additional fatigue-skill coupling effect. A flatter trajectory indicates lower sensitivity to the additional effects of skill heterogeneity and fatigue accumulation. The results show that IDQN maintains the strongest robustness under coupled skill-fatigue heterogeneity.

All 15 test scenarios identical to those in section “IDQN outperforms baselines in scheduling performance and dynamic robustness” are used, with each setting run independently 20 times per scenario. All algorithms use policy weights trained under the homogeneous baseline setting Group A, with no retraining whatsoever—that is, a zero-shot transfer test. The evaluation metrics are the average Makespan under each setting and the corresponding PDR relative to setting Group A. In addition, to characterize the change in Makespan variance for each algorithm under coupled heterogeneity, the Makespan standard deviation under Group C is additionally reported. The experimental results are summarized in Figure 6 and Table S8.

As shown in Table S8, the Makespan degradation trajectories of the four algorithms across the three experimental settings display clear hierarchical differentiation, revealing the intrinsic differences among scheduling paradigms under heterogeneous labor force pressure.

In Group B, which introduces only skill heterogeneity, the PDR ranking of the four algorithms already preliminarily reflects differences in their respective adaptability: GA degrades most significantly (6.0%), followed by rule-based scheduling (4.8%), while standard DQN (3.8%) and IDQN (2.4%) degrade the least. The significant degradation of GA originates from the inherent vulnerability of its static pre-scheduling scheme to skill heterogeneity: GA uses the execution time upper bound (*t*_*max*_/*s*_*min*_ = 80s) as a conservative estimate to generate the assignment plan, but when one-third of skilled workers’ actual completion times are systematically lower than this upper bound, the critical path timing assumption of the pre-schedule is violated, and GA cannot respond during execution. Online methods (DQN, Rule-based, IDQN) can all partially absorb the disturbance introduced by skill differences through real-time state observation, and thus their degradation is relatively moderate. IDQN achieves the lowest degradation in Group B (2.4%), already outperforming standard DQN (3.8%), owing to the implicit awareness of executor heterogeneity formed during training through dynamic priority experience replay: even though fatigue drift has not yet been introduced in Group B, skill differences alone already produce a partial shift in the state distribution, and the dynamic priority mechanism’s preferential attention to high-TD-error experiences makes IDQN more resilient to the single-factor disturbance of skill heterogeneity.

Upon entering Group C, which simultaneously introduces skill heterogeneity and fatigue decay, the degradation magnitudes of the four algorithms diverge significantly, fully exposing the structural differences among scheduling paradigms under coupled disturbance. GA’s PDR jumps sharply from 6.0% in Group B to 16.5%, an increment of 10.5 percentage points—the largest increase among the four algorithms; the Makespan standard deviation simultaneously surges from 73.1s to 94.2s, with output stability severely compromised. This sharp degradation is a structural failure of the pre-scheduling paradigm when confronted with within-shift temporal drift: fatigue-induced systematic drift causes the pre-scheduled critical path estimate to fail continuously, while GA is entirely unable to respond during execution. Rule-based scheduling’s PDR rises from 4.8% to 9.7%, an increment of 4.9 percentage points; standard DQN’s rises from 3.8% to 7.7%, an increment of 3.9 percentage points. By contrast, IDQN’s PDR rises from 2.4% to 6.5%, an increment of only 3.1 percentage points—the lowest absolute degradation in Group C (6.5%) and the smallest PDR increment from Group B to Group C among all four algorithms; the Makespan standard deviation increment (26.1–17.6 = 8.5 s) is likewise substantially lower than that of DQN (13.8 s), Rule-based (14.3 s), and GA (29.1 s), demonstrating the strongest output stability.

The full manifestation of IDQN’s robustness advantage in Group C originates from the intrinsic alignment of its two core design features with the challenges of coupled heterogeneity. First, $T_{e}^{\text{remain}}\left(t\right)$ as a state input allows the policy to directly observe the actual deviation in current execution progress at each decision step—fatigue causes workers’ later-task completion times to be systematically longer, and this information enters the decision basis directly through $T_{e}^{\text{remain}}\left(t\right)$; IDQN can implicitly perceive the fatigue level of executors through the signal “the current task is observed to be slower than expected” and dynamically adjust subsequent assignments, without explicitly modeling the fatigue state. Second, the advantage of dynamic priority experience replay is fully activated in Group C: novices with advanced fatigue, when executing late-stage tasks, generate actual time costs far exceeding the Q-value estimate, forming rare transitions with large TD errors; the dynamic priority mechanism samples such high-deviation experiences preferentially by weighting with TD error, enabling Q-values to adapt to fatigue states far faster than the standard DQN with uniform replay. This also directly explains why, from Group B to Group C, IDQN’s PDR increment (3.1 percentage points) is smaller than that of standard DQN (3.9 percentage points): the introduction of fatigue drift is precisely the activating condition under which the dynamic priority mechanism generates a differentiated advantage over uniform replay.

Synthesizing the results across the three settings, this section confirms IDQN’s robustness to heterogeneous labor force conditions from three dimensions: In terms of absolute degradation magnitude in Group C, IDQN (6.5%) is substantially lower than GA (16.5%), rule-based (9.7%), and standard DQN (7.7%); In terms of PDR increment from Group B to Group C, IDQN (3.1 percentage points) is the smallest among all four algorithms; In terms of output stability, IDQN’s Makespan standard deviation increment in Group C (8.5s) is far lower than that of the other comparison algorithms. The above results confirm that IDQN’s capacity for handling temporal uncertainty does not rely on the uniform distribution assumption, and can be effectively generalized to realistic heterogeneous labor force conditions involving the coupled effects of skill and fatigue.

### IDQN generalizes to out-of-distribution scenarios with near-in-distribution performance

To evaluate whether the trained IDQN policy can generalize to scenarios outside the training distribution without retraining, 20 out-of-distribution (OOD) test scenarios were constructed along five structurally independent shift dimensions. The in-distribution (ID) baseline is defined as the mean Makespan of standard test scenarios T1–T15 (each run independently 20 times). All OOD scenarios are evaluated using the policy weights learned on the 70 training scenarios, without any fine-tuning, weight updates, or hyperparameter modifications.

The five shift dimensions are defined as follows. D1 tests scale extrapolation in task count: four scenarios with |*J*| ∈ [60,90,120,150] exceed the training upper bound of |*J*| = 50, with the maximum extrapolation reaching three times the training scale. D2 tests joint scale extrapolation in executor count: In four scenarios, both *N* and *K* exceed the training upper bound, thereby testing the zero-padding mechanism that handles variable executor pool sizes. D3 tests DAG topology extrapolation: four scenarios feature DAG depths of 7–10 layers with up to 4 predecessors per task, whereas the training range is depths of 2–6 layers with at most 2 predecessors, resulting in longer critical paths and more constrained scheduling flexibility. D4 tests extreme shared workspace proportions: Four scenarios are positioned at boundary extremes (*σ* ∈ [0.05,0.10,0.70,0.85]), far below or above the training range of 0.20–0.60, stress-testing the PPD framework. D5 tests a structural shift in task type composition: Four scenarios consist entirely of HRF-type tasks—a composition the policy has never encountered during training—requiring it to make all allocation decisions solely based on executor workload status and availability, rather than task-type constraints. Each OOD scenario is run independently 20 times, yielding a total of 400 OOD evaluations. The primary evaluation metrics are the mean Makespan and its standard deviation. The experimental results are summarized in Table 4.

**Table 4:** Zero-shot generalization test results on out-of-distribution scenarios (mean ± standard deviation across 20 independent runs per scenario)

Dimension	Scenario	|*J*|	|*E*|	Key OOD deviation	Average makespan (s)	Makespan standard deviation (s)
D1	OOD1	60	22	task scale × 1.2	327.6	24.3
	OOD2	90	26	task scale × 1.8	378.4	31.2
	OOD3	120	28	task scale × 2.4	443.7	38.6
	OOD4	150	29	task scale × 3.0	507.3	46.4
D1 mean					414.3	35.1
D2	OOD5	60	38	executor scale, |*E*|=38	319.4	22.7
	OOD6	90	51	executor scale, |*E*|=51	363.2	29.1
	OOD7	120	64	executor scale, |*E*|=64	421.8	36.9
	OOD8	150	77	executor scale, |*E*|=77	486.4	44.3
D2 mean					397.7	33.3
D3	OOD9	35	17	DAG depth 7–8, predecessors ≤3	371.4	33.8
	OOD10	40	22	DAG depth 7–8, predecessors ≤3	396.3	37.2
	OOD11	45	25	DAG depth 8–9, predecessors ≤4	427.2	41.1
	OOD12	50	31	DAG depth 9–10, predecessors ≤4	463.5	46.4
D3 mean					414.6	39.6
D4	OOD13	35	17	*σ*= 0.05(very low collision risk)	271.3	18.4
	OOD14	40	22	*σ*= 0.10 (low collision risk)	280.6	19.7
	OOD15	40	22	*σ*= 0.70 (high collision risk)	362.4	37.8
	OOD16	45	25	*σ*= 0.85 (very high collision risk)	406.3	47.6
D4 mean					330.2	30.9
D5	OOD17	35	17	pure HRF	301.2	20.8
	OOD18	40	22	pure HRF	306.4	21.7
	OOD19	45	25	pure HRF	313.8	22.6
	OOD20	50	31	pure HRF	321.4	23.9
D5 mean					310.7	22.3
ID baseline					310.8	16.2

As shown in Table 4, the trained IDQN policy produced valid scheduling solutions across all 400 OOD runs spanning the five shift dimensions. The experimental results exhibit a clear hierarchical pattern in the degree of Makespan degradation. D1 and D2 both show monotonically increasing absolute Makespan values as the degree of extrapolation grows. Across both dimensions, the relative performance gap between OOD and ID conditions narrows as problem scale increases. This narrowing is a natural characteristic of zero-shot transfer protocols rather than an indication of model failure: In both D1 and D2, executor count scales alongside task count, so the increased parallelism partially offsets the additional task load, and Makespan grows sub-linearly with problem size. D5 (pure HRF task composition) achieves a mean Makespan of 310.7 s, nearly identical to the ID baseline. When all tasks are of HRF type, executor assignment is no longer constrained by task-type restrictions, allowing both humans and robots to execute any task. This complete flexibility does not lead to performance degradation; rather, it enables the scheduler to achieve a degree of parallelism nearly equal to or better than the training distribution. The mild upward trend from OOD17 (301.2 s) to OOD20 (321.4 s) merely reflects the incremental increase in scale as task count grows from 35 to 50. D4 (extreme shared workspace proportions) produces asymmetric results that directly validate the PPD framework’s bidirectional responsiveness. Scenarios with *σ* below the training range (OOD13: 271.3 s, OOD14: 280.6 s) yield Makespan values lower than the ID baseline: When fewer tasks require shared workspace monitoring, the frequency of safety-triggered robot pauses decreases, saving additional time. Conversely, scenarios with *σ* above the training range (OOD15: 362.4 s, OOD16: 406.3 s) exhibit a marked Makespan increase: Under the extreme condition of *σ*= 0.85, near-universal collision monitoring leads to frequent and prolonged robot safety pauses that accumulate into substantial scheduling delays. D3 (deep DAG topology) produces the largest Makespan increase among dimensions tested within the training task-count range, with the mean rising from 371.4 s at OOD9 to 463.5 s at OOD12. This pronounced degradation relative to the ID baseline reflects the fundamental cost of increased task serialization: when DAG depth reaches 9–10 layers with up to 4 predecessors per task, the proportion of tasks that can be executed in parallel at any given moment decreases as the critical path lengthens. Unlike the safety-pause cost in D4, which can be partially offset by better scheduling decisions, the serialization cost imposed by deep DAGs is an unavoidable constraint inherent to the dependency structure itself—regardless of the quality of allocation decisions, the policy must wait for predecessor tasks to complete. The constraint-based action masking mechanism correctly enforces these deep dependency chains without modification, confirming that the Makespan increase reflects the elevated complexity of the problem itself rather than a failure of policy capability.

## Discussion

Addressing the challenges posed by the dual uncertainty of human behavior (temporal uncertainty in execution time and spatial uncertainty in position) to production scheduling and safety assurance in HRC workshops, this paper proposes a dynamic task scheduling method for large-scale HRC oriented toward human dual uncertainty. First, to address the spatial uncertainty of humans, a systematic PPD optimization framework is constructed: At the perception layer, a differentiated link resource allocation model based on AoI is established, which significantly reduces the transmission latency of critical safety information by prioritizing the allocation of limited communication bandwidth to executors with high collision risk tasks; at the prediction layer, a DT elastic deployment model is constructed, which ensures that DTs of high-priority executors can always be deployed nearby under edge resource-constrained conditions through bidirectional constraints between task scheduling and DT deployment; at the decision layer, a collision prediction and proactive safety response mechanism based on probabilistic occupancy is proposed, which precisely quantifies collision risk using forward reachable sets and triggers robot action pauses for proactive obstacle avoidance. Second, to address the temporal uncertainty of humans, an IDQN scheduling algorithm integrating action masking and dynamic priority experience replay is proposed, achieving real-time dynamic scheduling decisions during task execution. Simulation experiments across 15 test scenarios of varying scales validate the effectiveness of the proposed method. Regarding safety, the PPD framework’s three-layer cascading optimization reduces the collision response trigger count by 59.9% compared to the baseline (from 44.6 to 17.9 times per run); while this metric does not guarantee the absolute elimination of physical contact, it effectively reflects the framework’s systematic suppression of collision risk, and demonstrates that the coordinated suppression of false collision triggers across the perception, prediction, and decision layers translates directly into measurable reductions in unnecessary robot interruptions, thereby mitigating the degradation of task efficiency caused by safety response mechanisms. Regarding efficiency, the average Makespan is reduced by 12.1%, a gain that exceeds the sum of individual layer contributions (9.2%), confirming the existence of positive cross-layer coupling effects identified in the ablation study. Taken together, these results validate the proposed framework’s capacity for simultaneous improvement in collision risk reduction and scheduling efficiency in large-scale HRC scenarios.

### Limitations of the study

The research in this paper still has the following limitations that warrant further exploration:

(1) The temporal uncertainty model employs a uniform distribution to represent human execution durations. This choice is the maximum-entropy prior over the observed feasible interval, providing worst-case robustness guarantees within the class of bounded-support distributions. However, in real industrial environments, execution durations may exhibit non-uniform patterns—such as right-skewed distributions reflecting fatigue effects, or bimodal distributions reflecting heterogeneity across workers from different shifts. In the future, data-driven methods—such as Bayesian nonparametric models or mixture distributions fitted to real workshop records—can be adopted to dynamically learn the distribution characteristics of human execution behavior, thereby improving the model’s adaptability to diverse environments. Similarly, the isotropic Gaussian assumption in the spatial uncertainty model ignores strict biomechanical joint constraints, which in reality render limb displacement probabilities directionally non-uniform. Future work can extend the current isotropic formulation to an anisotropic covariance model parameterized by motion capture data or task-specific biomechanical studies, further improving the fidelity of collision probability estimation;
(2) The collision prediction in this paper is based on pose information from a single sensor; in the future, multi-modal perception data (such as vision, force sensing, and so forth) can be fused, combined with attention mechanisms to further improve the accuracy of human intention recognition and trajectory prediction.
(3) This paper assumes that the computational resources of edge servers are constant; in the future, more complex cloud-edge collaboration scenarios such as dynamic fluctuations in server load and network congestion can be considered to further enhance the robustness and practicality of the method.
(4) Comparative experiments demonstrate that IDQN, as a centralized single-agent scheduling solver with domain-specific design choices, achieves competitive scheduling performance relative to MARL methods such as QMIX and MAPPO under the HRC temporal-uncertainty setting: The mean Makespan (315.9 s) is only marginally higher than MAPPO (313.7 s, a gap of 0.7%) and superior to QMIX (321.4 s); the optimal-solution acquisition rate (74.1%) is substantially higher than both QMIX (61.8%) and MAPPO (62.4%). In terms of dynamic robustness, IDQN’s PDR (2.5%) is comparable to QMIX (2.2%) and MAPPO (2.0%), with all three demonstrating good robustness. Meanwhile, QMIX and MAPPO retain complementary architectural advantages: QMIX’s value-decomposition framework provides principled multi-agent credit assignment, and MAPPO’s policy-gradient approach enables exploration of a richer policy space. Future work could investigate hybrid architectures that combine the value decomposition or actor-critic advantages of QMIX/MAPPO with IDQN’s HRC-specific constraint handling and robustness mechanisms.
(5) The algorithmic performance reported in this paper is achieved in a simulation environment under specific model assumptions: human limb acceleration bounded by *a*_*max*_, a deterministic AoI model, and instantaneous robot pausing. Under these conditions, the forward reachable set provably covers all physically possible limb positions within the AoI window. However, real physical deployment introduces conditions that go beyond these assumptions (including instantaneous accelerations exceeding *a*_*max*_, stochastic AoI variability caused by wireless channel fading, non-zero robot braking delays, and real sensor noise), any one of which could in principle allow a collision event to occur even when the PPD framework is active. Further enriching the model by incorporating more detailed physical-level constraints so as to validate algorithmic performance under real deployment conditions represents a priority direction for future research.
(6) The reward function of the current IDQN algorithm optimizes solely for makespan via a per-step time penalty, without incorporating operational cost metrics such as mechanical wear or energy consumption; the scheduling policy is therefore agnostic to the trade-offs between equipment longevity and energy efficiency. Because the PPD hard-stop is enforced by the constraint layer independently of the reward signal, introducing wear or energy consumption as secondary penalty terms into the reward function would not destabilize the PPD constraint. Accordingly, designing a multi-objective reward formulation that balances makespan against mechanical wear and energy consumption, and validating it through higher-fidelity actuator simulations, represents an important direction for future work.

## Resource availability

### Lead contact

Requests for further information and resources should be directed to and will be fulfilled by the lead contact, Tong Huagang (c196537@njtech.edu.cn).

### Materials availability

This study did not generate new unique reagents.

### Data and code availability

• The data generated in this study, including experiment configuration files, scenario definitions, run-level simulation outputs, and training logs, have been deposited at Mendeley Data and are publicly available. The DOI is listed in the key resources table: Mendeley Data: https://doi.org/10.17632/d33926c68z.1.
• The source code supporting the scheduling experiments, simulations, and analyses reported in this paper has been deposited at Mendeley Data and is publicly available. The DOI is listed in the key resources table: Mendeley Data: https://doi.org/10.17632/d33926c68z.1.
• Any additional information required to reanalyze the data reported in this paper is available from the lead contact upon reasonable request.

## Acknowledgments

The work was supported by the Basic Science (Natural Science) Research Project of Higher Education Institutions of Jiangsu Province (23KJB62006), the Humanities and Social Sciences Program of the Ministry of Education (23YJCZH201), the Jiangsu Social Science Foundation (23GLLC015), the 10.13039/501100001809National Natural Science Foundation of China (72401128), the 10.13039/501100004608Natural Science Foundation of Jiangsu Province (BK20240537), the 10.13039/501100012456National Social Science Fund of China (21CJY055), and the Qing Lan Project of Jiangsu Provincial Higher Education Institutions (54905013).

## Author contributions

Conceptualization, Z.S. and H.T.; methodology, Z.S.; software, Z.S.; validation, Z.S.; formal analysis, Z.S.; investigation, Z.S.; writing – original draft, Z.S.; writing – review and editing, H.X. and H.T.; resources, H.X. and H.T.; funding acquisition, H.T.; supervision, H.T.

## Declaration of interests

The authors declare that they have no competing financial interests or personal relationships that could have influenced the work reported in this paper.

## Declaration of generative AI and AI-assisted technologies in the writing process

During the preparation of this work, the author(s) used DeepSeek in order to assist with language polishing, translation, and grammar checking. After using this tool, the author(s) reviewed and edited the content as needed and take(s) full responsibility for the content of the publication.

## STAR★Methods

### Key resources table

**Table:** 

REAGENT or RESOURCE	SOURCE	IDENTIFIER
**Deposited data**		

Experimental data, simulation outputs, scenario definitions, run-level outputs, and training logs	This paper; Mendeley Data	Mendeley Data: https://doi.org/10.17632/d33926c68z.1

**Software and algorithms**		

Source code for scheduling experiments	This paper; Mendeley Data	Mendeley Data: https://doi.org/10.17632/d33926c68z.1
Python 3.10	Python Software Foundation	https://www.python.org RRID: SCR_008394
PyTorch	Meta AI	https://pytorch.org/ RRID: SCR_018536
NumPy	NumPy Developers	https://numpy.org/ RRID: SCR_008633

### Method details

#### HRC task scheduling problem description

This paper focuses on large-scale automotive assembly HRC workshops. With the widespread application of collaborative robots in the automotive manufacturing industry, modern automotive assembly workshops generally adopt HRC workstation layouts, where each workstation is equipped with a human operator and a collaborative robot, collaboratively completing assembly tasks within a shared workspace.^24^

As shown in Figure S8, executors in the workshop can be classified into three categories: human executors $H=\left\{H_{1},H_{2},...,H_{N}\right\}$, collaborative robots $R=\left\{R_{1},R_{2},...,R_{N}\right\}$, and Autonomous Mobile Robots (AMR) $M=\left\{M_{1},M_{2},...,M_{K}\right\}$. Among them, H and R are responsible for assembly tasks, while M is responsible for material transportation tasks between workstations. The total set of executors is E=H∪R∪M, comprising 2*N*+*K* executors in total. The workshop is deployed with *N* HRC workstations, denoted as $W=\left\{W_{1},W_{2},...,W_{N}\right\}$. Each workstation is configured with one human operator and one collaborative robot, and the workstation worktable contains the robot’s exclusive workspace, the human’s exclusive workspace, and the shared workspace.

The set of tasks to be scheduled in the workshop is denoted as $J=\left\{J_{1},J_{2},...,J_{M}\right\}$., comprising *M* subtasks in total. Based on execution characteristics, each subtask can be pre-classified into one of the following three categories^24^: (1) Robot-Only Task (RO), denoted as $J^{RO}\subseteq J$, can only be executed by robots, with typical scenarios including high-load tasks, material transportation tasks, and high-precision repetitive assembly tasks; (2) Human-Only Task (HO), denoted as $J^{HO}\subseteq J$, can only be executed by human executors, with typical scenarios including high-dexterity fine assembly tasks, quality inspection tasks requiring cognitive judgment, etc.; (3) Human-Robot Flexible Task (HRF), denoted as $J^{HRF}\subseteq J$, can be executed by either humans or robots. The three task categories satisfy $J=J^{RO}\cup J^{HO}\cup J^{HRF}$ and are mutually disjoint. Before being scheduled, each subtask *J*_*j*_ possesses the inherent attributes shown in Table S9, which can serve as inputs to the scheduling algorithm.

Facing the dual uncertainty problem of temporal and spatial aspects of human executors, the core problem studied in this paper is how to reasonably allocate *M* subtasks to 2*N*+*K* executors in large-scale HRC workshops, and determine the execution sequence and start time of each task, so as to minimize the Makespan while maximally suppressing collision risk through the dual-layer safety architecture.

This paper addresses two types of human uncertainty that are physically distinct in nature. To establish the foundation for the mathematical models that follow, this section provides formal definitions of both types of uncertainty as follows:

##### Temporal uncertainty

The temporal uncertainty of human workers during task execution stems from individual differences in skill level, fatigue accumulation, and task familiarity. The execution duration of human executor *e* when assigned sub-task *J*_*j*_ is modeled as a random variable drawn independently from a bounded uniform distribution, $T_{j}^{H}∼U\left(t_{\min},t_{\max}\right)$.The specific value of $T_{j}^{H}$ is unknown to the scheduler at the time of task assignment and only becomes known upon task completion. This “unknown before completion” property is encoded in the state space of the IDQN algorithm via the remaining task time ${T_{e}}^{remain}\left(t\right)$, enabling the IDQN agent to learn to make decisions based on actual progress rather than prior estimates.

The uniform distribution is adopted for three reasons. First, in the absence of historical data for a specific worker-task combination, it is the maximum-entropy prior over a bounded support—that is, the least informative assumption when only the feasible interval is known.^25^ Second, it is the standard benchmark distribution used in the HRC scheduling literature for modeling variability in human task durations, ensuring direct comparability with prior work.^26^ Third, within the class of bounded-support distributions, it represents the hardest case for the scheduler, as it assigns equal probability to the fastest and slowest execution outcomes; a policy that performs well under U(*t*_*min*_,*t*_*max*_) is therefore robust to any distribution with the same support.

##### Spatial uncertainty

The spatial uncertainty of human workers arises from the unpredictability of limb trajectories during task execution. Due to communication latency (AoI = Δ), the human limb state observed by the digital twin is already Δ seconds stale. Spatial uncertainty is modeled as the set of all positions the limb may currently occupy, subject to the physiological acceleration constraint *a*_*max*_, and is quantified probabilistically via an isotropic Gaussian distribution whose covariance grows explicitly with Δ: *p*(*t*)∼*N*(*p*_*pred*_,*σ*(Δ)), where *σ*(Δ) = *σ*_0_ + *a*_*max*_·Δ^2^/2. Here *p*_*pred*_ is the constant-velocity predicted position and *σ*_0_ is the sensor baseline measurement uncertainty. This model is grounded entirely in physically derived parameters and real-time sensor observations, requiring neither motion capture datasets nor pre-trained trajectory models. Two properties make it particularly well-suited to the PPD framework: uncertainty grows quantitatively with AoI, establishing a direct cross-layer coupling between the perception layer (AoI management) and the decision layer (collision risk assessment); and the framework provides provably conservative safety guarantees that are independent of the specific execution trajectory. The complete derivation—including forward reachable set construction, spatial grid discretization, occupancy probability computation, and the collision probability formula—is presented in Section “Decision Layer: Collision Response Mechanism Based on Probabilistic Occupancy Grid”.

#### Mathematical model

##### Basic task scheduling model

Define binary variable *y*_*j*,*e*_ to indicate whether subtask *J*_*j*_ is assigned to executor *e* for execution: *y*_*j*,*e*_ = 1 indicates assignment, *y*_*j*,*e*_ = 0 indicates otherwise. Define continuous variables $t_{j}^{s}$ and $t_{j}^{e}$ to represent the start time and end time of subtask *J*_*j*_ respectively, where $t_{j}^{e}=t_{j}^{s}+T_{j,e}$, and *T*_*j*,*e*_ is the execution duration of subtask *J*_*j*_ when executed by executor *e*.

The optimization objective of this paper is to minimize the Makespan: $\min\;C_{\max}=\min\max_{j\in J}t_{j}^{e}$.

The scheduling problem must satisfy the following basic constraints: (1) Unique assignment constraint—each subtask must be assigned to exactly one executor for execution: $\sum_{e\in E}y_{j,e}=1,\forall J_{j}\in J$; (2) Predecessor constraint—for tasks with predecessor relationships, the predecessor task $t_{i}^{e}$ must be completed before the successor task $t_{j}^{s}$ starts: $t_{i}^{e}<t_{j}^{s},\forall i\neq j$; (3) Executor capacity constraint—at any time, each executor can execute at most one subtask, i.e., the current execution count $n_{e}^{tk}$ is 1: $n_{e}^{tk}\left(t\right)\leq 1,\forall e\in E,\forall t$; (4) Material constraint—in the material storage area of workstation *i* where executor *e* is located, executor *e* can execute task *J*_*j*_ if and only if the remaining material quantity *GS*_*i*_ satisfies the material requirement *G*_*j*_ of task *J*_*j*_ (*G*_*j*_<0 indicates AMR material transportation task, which increases inventory): $GS_{i}\left(t_{j}^{s}\right)\geq G_{j},\forall y_{j,e}=1$; (5) Load constraint—the remaining physical load *L*_*e*_ of executor *e* (human/robot) must at least satisfy the load requirement *F*_*j*_ of task *J*_*j*_ for *e* to execute task *J*_*j*_: $L_{e}\left(t_{j}^{s}\right)\geq F_{j},\forall y_{j,e}=1$; (6) Task type constraint—different task types have corresponding assignment restrictions on executors.

##### Perception layer: Differentiated link resource allocation model based on age of information

Humans exhibit spatial uncertainty during task execution: unlike robots with pre-programmable deterministic trajectories, human limbs may enter the shared workspace at any time. To suppress collision risk to personnel, the system needs to monitor the current position of human limbs in real-time, so as to detect potential collision hazards in a timely manner and trigger proactive responses. However, in large-scale HRC workshops, numerous executors need to share limited wireless communication bandwidth. If an equal resource allocation strategy is adopted for all executors, the state information of high-risk task executors cannot be transmitted with priority. When the state information transmission latency is too large, the system’s cognition of human limb positions is lagging, and information latency will directly compress the system’s response time window, increasing collision risk.

To this end, this section introduces the concept of Age of Information (AoI) and establishes a differentiated link resource allocation model based on collision risk as shown in Figure S9. The core idea is to prioritize the allocation of limited communication resources to executors with high collision risk tasks, reducing the AoI of their state information, ensuring that the system can detect potential collision risks in a timely manner.

First, define the collision risk level. The collision risk level of subtask *J*_*j*_ is defined as *ρ*_*j*_ = *σ*_*j*_ ∈ 0,1. When *σ*_*j*_ = 1 (shared workspace task), human limbs may enter the human-robot shared workspace during task execution, there is collision possibility, and real-time monitoring is required; when *σ*_*j*_ = 0 (exclusive workspace task), human limbs will not enter the shared workspace, there is no collision risk, and high-frequency monitoring is not required. This definition judges whether there is collision possibility based on the spatial attributes of the task itself; as long as the task involves shared workspace, regardless of how humans actually move, it should be considered as having collision risk.

The AoI of executor *e* at time *t*, Δ_*e*_(*t*), is defined as^27^ $\Delta_{e}\left(t\right)=t-t_{e}^{last}$. Here $t_{e}^{last}$ is the generation time of the state information of executor *e* most recently successfully received by the DT. Δ_*e*_(*t*) characterizes the degree of lag in the DT’s cognition of the current state of executor *e*; the smaller Δ_*e*_(*t*) is, the closer the state information held by the DT is to the true current state of the executor. For humans executing shared workspace tasks, the system needs to monitor the real-time distance between their limbs and the robot. Due to the existence of AoI, the monitored calculated value actually reflects the distance Δ seconds ago. This cognitive lag brings two types of risks: human limbs may have already entered the danger zone but the system based on lagging information still considers it safe; even if the system detects risk, the time window left for robot response is also compressed. Therefore, the lower the AoI, the more timely the safety monitoring, and the higher the safety level.

It is worth noting that the AoI model in this paper is purposefully simplified to serve a specific objective: to establish a quantitative causal chain from bandwidth allocation decisions, through information freshness, through uncertainty in collision risk estimation, and ultimately to the timeliness of safety responses. This causal chain is precisely the transmission path through which perception-layer resource allocation decisions affect decision-layer safety outcomes within the PPD framework. The simplified bandwidth–AoI mapping is sufficient to faithfully capture the essence of this causal chain: under the differentiated allocation strategy, it correctly ranks executors by information freshness and correctly predicts the direction and relative magnitude of the effect of bandwidth allocation on AoI and, in turn, on collision probability. Physical-layer phenomena such as channel fading and packet retransmission introduce stochastic perturbations at the entry node of this causal chain; incorporating them into the model enables a more fine-grained characterization of AoI, but does not alter the structure of the causal chain, nor does it invalidate the differentiated allocation strategy.

Based on the above analysis, we propose a differentiated resource allocation strategy. Define the resource allocation priority *π*_*e*_(*t*) of executor *e* at time *t* as follows: if e∈H and is executing a shared workspace task (*ρ*_*j*(*e*,*t*)_ = 1, where *j*(*e*,*t*) represents the subtask being executed by executor *e* at time *t*), then *π*_*e*_(*t*) = 1; otherwise *π*_*e*_(*t*) = 0. Let the set of high-priority executors (i.e., those executing high-risk tasks) at time *t* be $E^{high}\left(t\right)=\left\{e\in E|\pi_{e}\left(t\right)=1\right\}$, and the set of low-priority executors be $E^{low}\left(t\right)=\left\{e\in E|\pi_{e}\left(t\right)=0\right\}$. Adopting a priority-based bandwidth allocation strategy, when high-priority executors exist, allocate *β* proportion (*β* ∈ (0.5,1]) of the total available bandwidth *B*_*total*_ to high-priority executors, and allocate the remaining (1-*β*) proportion to low-priority executors.

The state information sampling period *δ*_*e*_(*t*) = *b*/*B*_*e*_(*t*) and transmission latency *D*_*e*_(*t*) = *b*/*B*_*e*_(*t*) of executor *e* are both related to its allocated bandwidth *B*_*e*_(*t*), where *b* is the data packet size of a single state information transmission. Therefore, the AoI of executor *e* can be approximately expressed as Δ_*e*_(*t*)≈*δ*_*e*_(*t*) + *D*_*e*_(*t*) = 2*b*/*B*_*e*_(*t*). High-priority executors obtain more bandwidth, have shorter sampling periods and transmission latencies, reduced AoI, and more timely safety monitoring.

Communication resource allocation must satisfy the following resource constraints: (1) Total bandwidth constraint: $\sum_{e\in E^{active}\left(t\right)}B_{e}\left(t\right)\leq B_{total}$, where $E^{active}\left(t\right)=E^{high}\left(t\right)\cup E^{low}\left(t\right)$ is the set of all active executors; (2) Minimum bandwidth constraint: $B_{e}\left(t\right)\geq B_{\min},\forall e\in E^{active}\left(t\right)$, ensuring that each active executor can at least transmit basic state information; (3) Active executor count constraint: $\left|E^{active}\left(t\right)\right|\leq B_{total}/B_{\min}$.

It is worth noting that the AoI model above is built on assumptions of a stable wireless channel and deterministic bandwidth allocation. In real industrial wireless environments (e.g., Wi-Fi 6 or 5G private networks), channel fading, packet retransmission, and queuing delays will superimpose random variability on top of the deterministic AoI values. These physical-layer effects can be understood as random perturbations applied at the entry node of the causal chain described above: they alter the specific AoI value of each link, but do not affect the validity of the differentiated bandwidth allocation strategy. In a physical deployment scenario, a more fine-grained characterization of uncertainty can be obtained by incorporating physical-layer stochasticity into the AoI model (for example, by employing stochastic network calculus to establish a probabilistic upper bound on AoI); such extensions do not affect the cross-layer coupling logic of the PPD framework proposed in this paper.

##### Prediction layer: DT elastic deployment model

In the safety assurance framework of this paper, the DT undertakes the core functions of state aggregation and distance monitoring: receiving real-time state information from executors, maintaining virtual mapping of physical entities, and calculating human-robot distance in real-time based on the current positions of humans and robots to determine collision risk. The previous section ensured that the state information of high-risk task executors can be transmitted to the edge network with lower AoI through differentiated link resource allocation. However, state information arriving at the edge network does not mean arriving at the DT—the deployment location of the DT determines the last-hop latency of state information from the edge network to the DT.

In the edge network, DTs can be deployed on cloud servers (abundant computational resources but higher transmission latency) or edge servers (closer to executors, lower transmission latency but limited computational resources).^5^ Assuming the DT of executor *e* is deployed on server *v*, the total latency of state information from the executor to the DT is $D_{e}^{total}=D_{e}^{link}+D_{e}^{server}\left(v\right)$, where $D_{e}^{link}$ is the communication link transmission latency, $D_{e}^{server}\left(v\right)$ is the transmission latency from the edge network to server $v\in V^{edge}$, and $V^{edge}$ is the set of edge servers. The larger the total latency, the more severe the cognitive lag of the DT regarding the current state of the executor, and the later the timing of detecting collision risk. Due to limited computational resources of edge servers, when DTs of multiple executors all wish to be deployed on the same edge server, resource insufficiency may occur. Therefore, a DT dynamic deployment strategy needs to be established: under edge resource constraints, prioritize deploying DTs of high collision risk task executors to nearby edge servers to reduce their total latency.

Consistent with the bandwidth allocation priority, define the DT deployment priority *φ*_*e*_(*t*) of executor *e* at time *t*: if e∈H and is executing a shared workspace task, then *φ*_*e*_(*t*)=1; otherwise *φ*_*e*_(*t*)=0. The deployment priority is kept consistent with the resource allocation priority, ensuring that high-risk task executors receive priority assurance in both communication resources and deployment location dimensions. For each executor e, define its nearby edge server as $v_{e}^{near}=\arg\min_{v\in V^{edge}}d\left(e,v\right)$. The transmission latency from the edge network to server *v* is positively correlated with the physical distance *d*(*e*,*v*), where *α* is the latency coefficient: $D_{e}^{server}\left(v\right)=\alpha \cdot d\left(e,v\right)$.

Define binary decision variable *x*_*e*,*v*_(*t*): equals 1 if the DT of executor *e* is deployed on server *v* at time *t*, otherwise 0. Deployment must satisfy the following constraints: (1) Unique deployment constraint: $\sum_{v\in \left(V^{edge}\cup v^{cloud}\right)}x_{e,v}\left(t\right)=1,\forall e\in E,\forall t$; (2) High-priority must be deployed on edge: $\sum_{v\in V^{edge}}x_{e,v}\left(t\right)=1,\forall e\in E^{high}\left(t\right)$, ensuring that for human executors executing shared workspace tasks, their DTs must be deployed on edge servers to guarantee the timeliness of safety monitoring; (3) Edge server resource capacity constraint: $\sum_{e\in E}x_{e,v}\left(t\right)\cdot C^{\min}\leq C_{v}^{\max},\forall v\in V^{edge}$, where *C*^*min*^ is the minimum resource required for DT operation, and $C_{v}^{\max}$ is the resource capacity of edge server *v*. In this paper, edge server resources are modeled using a normalized logical resource unit that abstracts the combined computational memory of the edge server. Each DT instance requires *C*^*min*^ standardized units to sustain its state synchronization and collision probability computation, and each edge server can concurrently host up to $C_{v}^{\max}/C^{\min}$ DT instances. This modeling approach preserves the structural constraint that edge resources are finite and contested—the mechanism driving the bidirectional coupling between DT deployment and task scheduling—while avoiding dependence on hardware-specific FLOPs or memory specifications, thereby ensuring the generality of the model.

When edge server resources are insufficient to simultaneously accommodate DTs of all high-priority executors, task scheduling must make adjustments. Define the necessary condition for shared workspace task *J*_*j*_ to be assigned to executor *e* at time *t* as: $\left(\left|E^{high}\left(t\right)\right|+1\right)\cdot C^{\min}\leq \sum_{v\in V^{edge}}C_{v}^{\max}$. If this condition is not satisfied, task *J*_*j*_ is an infeasible assignment to executor *e* at the current time, and the scheduling system should assign other executable exclusive workspace tasks to executor *e*, or temporarily wait until other high-priority executors complete tasks and release edge resources.

Based on the above constraints, the dynamic deployment rules are as follows: if *φ*_*e*_(*t*) = 1 (high-priority), deploy the DT to the nearby edge server $v_{e}^{near}$; if *φ*_*e*_(*t*)=0 (low-priority), deploy to the cloud server *v*^*cloud*^. In this way, DTs of high-priority executors are forced to be deployed on the edge to ensure minimum transmission latency, while DTs of low-priority executors are deployed on the cloud so as not to occupy edge resources. The differentiated link resource allocation reduces $D_{e}^{link}$, and the dynamic deployment strategy in this section reduces $D_{e}^{server}$; the two work synergistically to minimize the total latency of high-risk task executors.

It is worth noting that the DT elastic deployment strategy described in this section instantiates a core capability of DT technology—the virtual-real interaction closed loop: the physical state of each executor (limb position, velocity, and load) is continuously synchronized to its corresponding DT virtual replica; the DT uses the virtual replica to compute collision probabilities in real time and feeds the results back into the decision process of the scheduling algorithm; the scheduling decision is ultimately translated into control commands for the physical robot’s motion, thereby completing the full “physical-virtual-physical” interaction cycle. The elastic deployment model, by prioritizing edge resources for high-risk executors, ensures that the end-to-end latency of this closed loop is minimized for high-risk executors—a critical condition for the effective operation of virtual-real interaction in safety-critical real-time applications. Furthermore, the DT virtual environment also provides support for the iterative optimization of scheduling decisions: in the IDQN algorithm, candidate task assignment schemes will be evaluated through DT virtual forward simulation before the final decision is committed, enabling the system to select more favorable scheduling decisions from both the efficiency and safety perspectives.

The DT deployment model in this paper treats edge server resource capacity and latency coefficients as fixed and known parameters in the simulation environment. In a real deployment scenario, edge server resources will fluctuate dynamically due to competing workloads from co-located applications, and latency coefficients will also change with variations in network topology and wireless channel conditions. Furthermore, the DT migration process itself will incur state synchronization overhead, which has not yet been modeled in the current framework. However, the differentiated DT deployment strategy adopted in this paper—which prioritizes deploying the DT of high-risk executors to the nearest edge server—remains directionally valid under the above dynamic conditions, because the relative ranking of executors by risk level is independent of absolute latency values. The primary adaptation work required for physical deployment includes: experimentally calibrating latency coefficients for the specific edge hardware configuration, and introducing a DT migration cost term into the scheduling constraints to balance migration overhead against latency gains.

##### Decision layer: Collision response mechanism based on probabilistic occupancy grid

The spatial uncertainty of human limbs is characterized through physics-constrained forward reachable sets and probabilistic occupancy grids, with the magnitude of uncertainty growing explicitly with AoI, thereby establishing a direct quantitative coupling between perception-layer bandwidth allocation and decision-layer collision risk assessment. The choice of a physics-constrained reachability framework over a data-driven trajectory prediction model is based on two reasons: first, this framework enables tractable real-time computation on the edge server without requiring the prior collection of motion datasets; second, it establishes an explicit quantitative coupling between AoI and collision risk, which is precisely the core mechanism through which the PPD framework achieves cross-layer integration.

As mentioned above, the human state information received by the DT has an AoI Δ, meaning the DT observes the human state from Δ seconds ago rather than the current true state. Let the human limb position observed by the DT at time *t* be *p*^*obs*^ and velocity be *v*^*obs*^, then *p*^*obs*^=*p*(*t*-Δ),*v*^*obs*^=*v*(*t*-Δ). Within this Δ-second cognitive blind spot, human limbs may have already moved to a new position, but the DT cannot know their current true position with certainty. This paper adopts the Reachability Analysis paradigm: instead of trying to predict where humans will reach, it analyzes all positions humans may possibly reach, constructs the Forward Reachable Set, and combines it with Probabilistic Occupancy Grid to quantify the occupancy possibility of each spatial cell. This idea draws on research achievements in the field of robot safety planning in recent years.^11^^,^^28^^,^^29^

For ease of calculation and judgment, the continuous workspace is discretized into 3D grids. On each grid cell, an occupancy probability is defined, representing the possibility that the cell is occupied by human limbs at the current time. This modeling approach combines the forward reachable set that describes the spatial range humans may reach and the probabilistic occupancy grid that quantifies the occupancy possibility of each spatial cell.^7^^,^^8^ First, discretize the workspace W of the HRC workstation into cubic cells with edge length *l*: $U=\left\{u_{i,j,k}|i\in \left[1,N_{x}\right],j\in \left[1,N_{y}\right],k\in \left[1,N_{z}\right]\right\}$, where *N*_*x*_,*N*_*y*_,*N*_*z*_ are the number of cells in the x, y, z directions respectively. The center coordinates of a spatial cell are *c*_*i*,*j*,*k*_ = ((*i*-0.5)*l*,(*j*-0.5)*l*,(*k*-0.5)*l*). The setting of cell edge length *l* requires balancing collision detection precision and computational efficiency: the smaller *l* is, the higher the spatial resolution, the more precise the collision detection, but the more cells there are, the greater the computational overhead.

During the AoI Δ, humans may continue moving at the observed velocity, or may change movement direction or speed magnitude, but limb acceleration is physiologically limited to a maximum value *a*_*max*_. Considering the maximum acceleration constraint, the velocity reachable set of human limbs is $V^{reach}\left(\Delta \right)=\left\{v|∥v-v^{obs}∥\leq a_{\max}\cdot \Delta \right\}$, which is a sphere centered at *v*^*obs*^ with radius *a*_*max*_·Δ. The outer boundary of the position reachable set is $P^{reach}\left(\Delta \right)=\left\{p|∥p-p^{pred}∥\leq r^{reach}\left(\Delta \right)\right\}$, where *p*^*pred*^ = *p*^*obs*^ + *v*^*obs*^·Δ is the predicted position assuming velocity remains unchanged, *r*^*reach*^(Δ) = *a*_*max*_·Δ^2^/2 is the reachable radius caused by acceleration uncertainty, and human limbs may appear at any position within a spherical region with radius *r*^*reach*^(Δ). Discretizing the position reachable set, the reachable spatial cell set is $U^{reach}\left(\Delta \right)=\left\{u\in U|u\cap P^{reach}\left(\Delta \right)\neq \emptyset \right\}$, representing all spatial cells that intersect with the position reachable set constitute the reachable spatial cell set.

For each cell *u* in the reachable spatial cell set, define its occupancy probability *P*(*u*) representing the possibility that human limbs occupy that cell at the current time. Assuming the actual human position follows a three-dimensional Gaussian distribution centered at the predicted position,^30^ i.e., $p\left(t\right)∼N\left(p^{pred},\Sigma \left(\Delta \right)\right)$, where the covariance matrix Σ(Δ) = *σ*^2^(Δ)·**I**_3_ reflects position uncertainty and is positively correlated with AoI Δ, where *σ*(Δ) = *σ*_0_ + *a*_*max*_·Δ^2^/2, *σ*_0_ is the baseline sensor measurement uncertainty under stable, unoccluded conditions, calibrated by taking repeated depth measurements of a static target at the nominal operating distance with the deployed sensor (e.g., an RGB-D camera) and computing the standard deviation of the results, **I**_3_ is the 3 × 3 identity matrix. In the absence of prior knowledge about human movement preference directions, an isotropic assumption is adopted, ignoring strict biomechanical joint constraints, i.e., uncertainty is assumed equal in all three spatial directions with no correlation. The formula *σ*(Δ) = *σ*_0_ + *a*_*max*_·Δ^2^/2 is derived from a worst-case kinematic upper bound under maximum acceleration constraint *a*_*max*_: given that the DT last observed a human limb at time *t*- Δ, within the AoI window the limb may have accelerated in any direction at up to *a*_*max*_, so the maximum additional positional displacement from the observed position is bounded above by *a*_*max*_·Δ^2^/2, which follows directly from the kinematic relationship for uniformly accelerated motion. The forward reachable set is constructed to contain all physically possible positions within the given acceleration constraint, making the uncertainty bound a conservative, deterministic bound. The occupancy probability of spatial cell *u* is obtained by integrating the Gaussian probability density function within that cell, and normalized within the reachable spatial cell set. The larger the AoI, the greater the position uncertainty, the larger the reachable set, and the higher the probability of the system misjudging collisions.

Let the robot end-effector position at time *t* be *p*_*R*_(*t*), and the set of spatial cells it occupies be $U_{R}\left(t\right)=\left\{u\in U|d\left(c_{u},p_{R}\left(t\right)\right)\leq r_{R}\right\}$, where *r*_*R*_ is the equivalent radius of the robot end-effector. The probability of collision between human limbs and the robot is the probability of overlap between the spatial cell sets occupied by both, i.e., the sum of probabilities that all spatial cells currently occupied by the robot are occupied by human limbs: $P_{collision}\left(t\right)=\sum_{u\in U_{R}\left(t\right)}\tilde{P}\left(u\right)$.

The above collision prediction mechanism is built upon four simplifying assumptions, whose rationale and applicable scope warrant explicit clarification. First, the maximum acceleration constraint *a*_*max*_, as the physiological upper limit for intentional upper-limb movement during normal manufacturing operations, provides the theoretical basis for the coverage guarantee of the forward reachable set; this upper limit may be transiently exceeded during emergency or reflexive movements, but in such extreme circumstances the SSM hard-stop mechanism^31^ will provide a safety backstop. Second, the isotropic Gaussian uncertainty model is the maximum-entropy distribution when only the magnitude of uncertainty is known and prior information about the direction of movement is absent, making it the information-theoretically optimal choice; its cost is a potential overestimation of uncertainty in directions where the probability of real movement is low. Third, the quadratic mapping from AoI to positional uncertainty is a worst-case kinematic upper bound, which may systematically overestimate the reachable set for typical sub-maximal movements. Fourth, the single-sensor baseline uncertainty *σ*_0_ assumes stable, unoccluded observation conditions. All four assumptions share a unified conservative design principle: systematically overestimating risk in order to minimize the possibility of missed detections of safety incidents within the model’s stipulated constraint envelope. The implications of each assumption for reliability in physical deployment will be further discussed in the subsequent physical deployment notes and the limitations analysis in the Discussion section.

Based on collision probability, the system determines whether to intervene in robot actions according to the intervention mechanism: $x_{rs}\left(t\right)=\left\{ \begin{array}{cc} 1, & P_{collision}\left(t\right)\geq \theta_{safe}\\ 0, & P_{collision}\left(t\right)<\theta_{safe} \end{array}\right.$, where *θ*_*safe*_ is the preset risk threshold. When collision probability exceeds the threshold, the robot immediately pauses its action until the risk disappears. After the action pause ends, the robot will continue executing the subtask it was executing before the pause, manifested as extended task execution time. The duration of each robot pause is determined by the duration of the collision risk condition: the robot remains paused at every time step where *P*_*collision*_(*t*)≥*θ*_*safe*_, and immediately resumes when *P*_*collision*_(*t*)<*θ*_*safe*_. Since *P*_*collision*_(*t*) depends on the current position of the human limb (which is governed by the human’s stochastic spatial motion), the pause duration is random and cannot be predicted at the time of task assignment. This is the mechanism by which spatial uncertainty propagates into the temporal dimension of the scheduling problem: due to human spatial uncertainty, the robot accumulates unpredictable additional delays beyond its nominal execution time, equal to the total duration of collision-induced pauses during task execution. In the model, this additional delay is represented through the state variable $T_{e}^{remain}\left(t\right)$: during robot pauses, $T_{e}^{remain}\left(t\right)$ remains frozen while the global clock continues to advance, so the cumulative pause duration is automatically encoded in the difference between elapsed wall-clock time and task progress. The duration of a robot’s action pause is in essence a form of second-order temporal uncertainty, induced by human spatial uncertainty and propagated through the safety response mechanism, which reveals the coupling relationship between temporal uncertainty and spatial uncertainty. With the optimization of AoI, the forward reachable set becomes more compact, collision probability estimation becomes more accurate, reducing the probability of false triggering collision response while ensuring personnel safety, enabling the system to operate more stably and efficiently.

In addition to the probability-based soft safety constraint described above, the system also requires a hard safety mechanism that can reliably prevent physical contact in any circumstance, independent of the accuracy of the probabilistic model. To this end, this paper introduces the Speed and Separation Monitoring (SSM) framework from ISO/TS 15066 (the international technical specification for collaborative robot safety) as the technical foundation for the hard safety constraint. The SSM framework has been thoroughly validated in industrial HRC applications: Karagiannis et al.^31^ demonstrated its effectiveness in a real collaborative workcell through adaptive safety zone switching; Faroni et al.^11^ established a formal theoretical foundation for embedding SSM safety constraints under uncertainty in human state estimation.

The core principle of SSM is: the actual separation distance *d*(*t*) between the human and the robot must at all times remain above the dynamic minimum protective separation distance *S*(*t*), which accounts for the relative approach velocity and the braking capability of the system. When *d*(*t*) falls below *S*(*t*) = *v*_*rel*_(*t*)·*T*_*stop*_ + *C*_*body*_, a safety-rated stop command is issued immediately, before physical contact can occur, where *v*_*rel*_(*t*) is the projected relative approach velocity between the human limb and the robot end-effector along the line connecting them, computed from the observed limb velocity *v*_*obs*_ and the robot end-effector velocity *v*_*R*_(*t*); *T*_*stop*_ is the total system response and braking time; *C*_*body*_ = *r*_*H*_ + *r*_*R*_ is the body geometry margin, defined as the sum of the cylindrical envelope radius of the human limb *r*_*H*_ and the cylindrical envelope radius of the robot end-effector *r*_*R*_. A hard stop is triggered when the condition shown in $x_{hard}\left(t\right)=\left\{ \begin{array}{l} 1,d\left(p_{R}\left(t\right),p_{H}\left(t\right)\right)<S\left(t\right)\\ 0,otherwise \end{array}\right.$ is satisfied.

The physical interpretation of this constraint is: *S*(*t*) is the minimum distance at which the robot must begin braking to guarantee that, given the current relative approach velocity, the robot comes to a complete stop before the human limb reaches the robot end-effector. When the human is stationary (*v*_*rel*_(*t*) = 0), *S*(*t*) degenerates to the pure geometric threshold *C*_*body*_. When the human approaches at *v*_*rel*_, *S*(*t*) = *v*_*rel*_(*t* )+ *C*_*body*_, and the robot begins braking when the human is at a distance of *v*_*rel*_(*t*) + *C*_*body*_, ahead of physical contact. This dynamic adaptation ensures that the hard stop triggers earlier when the approach velocity is higher, providing a safety guarantee that automatically tightens as risk increases. Critically, when *x*_*hard*_(*t*)=1, the robot stops immediately and unconditionally—independent of *P*_*collision*_(*t*), independent of the IDQN agent’s action selection, independent of the training or deployment phase, and independent of any probabilistic model.

The two-layer mechanism described above together constitute the dualayer safety architecture of this framework, with each layer having a clearly defined responsibility, a different type of constraint, and being logically independent from the other. Layer 1 (the PPD decision layer) is the probabilistic proactive soft safety layer: when *P*_*collision*_(*t*)≥*θ*_*safe*_, the robot proactively pauses before the human limb has yet reached a dangerous distance, substantially reducing the collision risk under the conditions where the model assumptions hold, but without providing a deterministic guarantee independent of the probabilistic model’s accuracy. Layer 2 (the SSM-based hard safety constraint) is the backstop hard safety guarantee: when *d*(*p*_*R*_(*t*),*p*_*H*_(*t*))<*S*(*t*), the robot immediately stops unconditionally, independent of the RL policy selection, the probabilistic model accuracy, and the training phase, grounded in the ISO/TS 15066 SSM framework.^31^ During *ε*-greedy exploration, both layers are simultaneously activated: Layer 1 proactively intercepts collision risk at the probabilistic level, while Layer 2 serves as the final backstop, ensuring that throughout the entire training process, physical contact events are provably prevented regardless of policy quality. The two layers are functionally complementary—Layer 1 intervenes proactively with a more generous distance margin, thereby reducing the frequency with which Layer 2 is triggered; Layer 2 uses a geometrically deterministic condition to guarantee physical safety when Layer 1 fails to respond in time.

The collision prediction model in this section treats the baseline sensor uncertainty *σ*_0_ as a known, fixed parameter within the Gaussian occupancy model. In actual physical deployment, the measurement noise of depth cameras or motion capture systems will introduce additional perturbations on top of *σ*_0_, increasing the effective positional uncertainty. Critically, an increase in additional sensor noise will cause *P*_*collision*_(*t*) to increase, making collision prediction more conservative rather than more permissive—that is, safety guarantees are maintained at any level of sensor noise, and the observable practical cost is a potential increase in the false-trigger rate. Therefore, the primary calibration task during physical deployment is: to experimentally measure the noise characteristics of the target sensor suite and adjust *θ*_*safe*_ and *σ*_0_ accordingly, so as to strike a reasonable balance between safety conservatism and operational efficiency. Furthermore, single-sensor pose estimation schemes are susceptible to occlusion interference in real workshop environments; multi-modal sensor fusion (e.g., combining depth cameras with wrist-mounted inertial measurement units) is a viable technical direction for improving the robustness of limb position estimation in physical deployments.

##### Integrated mathematical model

Integrating the above sub-models, the task scheduling problem in this paper involves two types of decision variables: *y*_*j*,*e*_ (whether subtask *J*_*j*_ is assigned to executor *e*) and $t_{j}^{s}$ (start time of subtask *J*_*j*_). The optimization model of this paper is as follows:

$$\min\;C_{\max}=\min\max_{j\in J}t_{j}^{e}$$

$$s.t.\;\sum_{e\in E}y_{j,e}=1,\forall J_{j}\in J$$

$$t_{i}^{e}<t_{j}^{s},\forall i\neq j$$

$$n_{e}^{tk}\left(t\right)\leq 1,\forall e\in E,\forall t$$

$$GS_{i}\left(t_{j}^{s}\right)\geq G_{j},\forall y_{j,e}=1$$

$$L_{e}\left(t_{j}^{s}\right)\geq F_{j},\forall y_{j,e}=1$$

$$\sum_{e\in E^{active}\left(t\right)}B_{e}\left(t\right)\leq B_{total},\forall t$$

$$B_{e}\left(t\right)\geq B_{\min},\forall e\in E^{active}\left(t\right),\forall t$$

$$|E^{active}\left(t\right)|\leq \lfloor B_{\text{total}}/B_{\min}\rfloor ,\forall t$$

$$\sum_{v\in \left(V^{edge}\cup v^{cloud}\right)}x_{e,v}\left(t\right)=1,\forall e\in E,\forall t$$

$$\sum_{v\in V^{edge}}x_{e,v}\left(t\right)=1,\forall e\in E^{high}\left(t\right),\forall t$$

$$\sum_{e\in E}x_{e,v}\left(t\right)\cdot C^{\min}\leq C_{v}^{\max},\forall v\in V^{edge},\forall t$$

$$\left(|E^{high}\left(t\right)|+1\right)\cdot C^{\min}\leq \sum_{v\in V^{edge}}C_{v}^{\max}$$

$$x_{rs}\left(t\right)=\left\{ \begin{array}{cc} 1, & P_{collision}\left(t\right)\geq \theta_{safe}\\ 0, & P_{collision}\left(t\right)<\theta_{safe} \end{array}\right.$$

The above model reveals the cascading influence mechanism among the three layers of perception-prediction-decision: the AoI at the perception layer directly determines the uncertainty boundary at the prediction layer, the response latency at the prediction layer constrains the available time window at the decision layer, and the three work together to address safety issues caused by spatial uncertainty of humans during task execution. Based on this cross-layer coupling relationship, the system is able to precisely allocate limited communication and computational resources to high-risk time windows and high-risk executors, thereby achieving coordinated optimization between collision risk reduction and resource allocation efficiency. Furthermore, the probabilistic soft safety layer described in *x*_*rs*_(*t*) and the SSM hard safety layer together constitute the two-tier safety assurance mechanism of this paper: the former proactively avoids collision risk through IDQN scheduling decisions, while the latter provides a deterministic fallback guarantee that is independent of the probabilistic model under any circumstances; the two are logically independent and functionally complementary.

##### Comprehensive algorithm of the PPD framework

Algorithm 1 presents the comprehensive operational logic of the PPD framework. The algorithm runs continuously during task execution, with discrete time steps advanced by system events (executor state updates, task completions, or collision detections). At each time step, the three layers execute in strict sequential order (Perception, Prediction, Decision), with the output of each layer serving as the input of the next, realizing cascaded coupling.

Algorithm 1 **Comprehensive algorithm of the PPD framework**

Input: Task set *J* = {*J*_1_,…,*J*_*M*_}, Executor set *E* = *H*∪*R*∪*M*, Edge server set *V*_*edge*_, Total bandwidth *B*_*total*_, Minimum bandwidth *B*_*min*_, High-priority bandwidth ratio *β*, Latency coefficient *α*, Collision probability threshold *θ*_*safe*_, Risk threshold *σ*_0_, *a*_*max*_.

Output: Real-time task scheduling decisions (*e*,*j*), Robot safety response *x*_*rs*_(*t*)

Initialization:

 Initialize all executor states *μ*_*e*_(*t*) = 0 (idle)

 Initialize AoI of all executors Δ_*e*_(*t*) = 0

 Initially deploy all DTs to cloud server *v*_*cloud*_

 Load the trained IDQN policy network

Main Loop: Repeat for each system event time *t* while not all tasks completed do:

 LAYER 1: PERCEPTION — AoI-Driven Bandwidth Allocation

 Classify executors by collision risk:

 *E*_*high*_(*t*) = {*e*∈*H*|*ρ*_*j*(e,t)_ = 1} // Humans executing shared-workspace tasks

 *E*_*low*_(*t*) = {*e*∈*E*|*μ*_*e*_(*t*) = 1,*e*∉*E*_*high*_(*t*)} // Other executors

 if *E*_*high*_(*t*) is not empty then:

 Allocate bandwidth:

 *B*_*e*_(*t*) = *β*·*B*_*total*_/|*E*_*high*_(*t*)| for *e*∈*E*_*high*_(*t*)

 *B*_*e*_(*t*) = 1-*β*·*B*_*total*_/|*E*_*low*_(*t*)| for *e*∈*E*_*low*_(*t*)

 subject to *B*_*e*_(*t*) ≥*B*_*min*_ for all active *e*

 else:

 Allocate bandwidth uniformly: *B*_*e*_(*t*) = *B*_*total*_/|*E*_*active*_(*t*)|

 end if

 Update AoI for all active executors

 LAYER 2: PREDICTION — Risk-Triggered DT Elastic Deployment

 r each executor *e* in *E* do:

 if *φ*_*e*_(*t*)=1 (*e*∈*E*_high_(*t*)):

 if $\left(\left|E_{high}\left(t\right)\right|+1\right)\cdot C_{\min}\leq \sum_{v}C_{v}^{\max}$: // Check edge capacity

 $x_{e,v_{e}^{near}}\left(t\right)=1$ // Deploy *DT*_*e*_ to nearby edge server

 $D_{e}^{server}=\alpha \cdot d\left(e,v_{e}^{near}\right)$ // Compute server latency

 else:

 Edge capacity insufficient: block shared-workspace task assignment

 Scheduling layer will not assign new shared-workspace tasks to *e*

 Maintain *DT*_*e*_ at cloud, flag e as edge-resource-constrained

 else:

 $x_{e,v_{cloud}}\left(t\right)=1$ // Deploy *DT*_*e*_ to cloud

 $D_{e}^{server}=D_{cloud}$ // fixed cloud latency

 end if

 $D_{e}^{total}\left(t\right)=D_{e}^{link}+D_{e}^{server}$ // Total latency

 end for

 LAYER 3: DECISION — Collision Prediction and Proactive Response

 for each *e*∈*E*_*high*_(*t*) do:

 Retrieve from DT: observed limb state (*p*_*obs*_,*v*_*obs*_) with staleness Δ_*e*_(*t*)

 Compute reachable radius: *r*_*reach*_=0.5·*a*_*max*_·Δ_*e*_(*t*)^2^

 Construct forward reachable set *P*_*reach*_(Δ)

 Compute occupancy probability for each cell *u* in *U*_*reach*_:

 *σ*(Δ) = *σ*_0_+0.5·*a*_*max*_·Δ_*e*_(*t*)^2^

 *p*(*t*)∼N(*p*_*pred*_,*σ*^2^·***I***_3_)

 *P*(*u*) = integral of *N* over cell *u*, normalized within *U*_*reach*_

 Compute collision probability:

 
$P_{collision}\left(t\right)=\sum_{u\in U_{R}\left(t\right)}\tilde{P\left(u\right)}$

 Determine safety response:

 if *P*_*collision*_(*t*)≥*θ*_*safe*_:

 *x*_*rs*_(*t*)=1 // robot pauses; task remaining time $T_{e}^{remain}$ unchanged

 else:

 *x*_*rs*_(*t*)=0 // robot continues

 # SSM-Based Hard Safety Constraint

 Compute *v*_*rel*_(*t*); compute *S*(*t*)

 if *d*(*p*_*R*_(*t*),*p*_*H*_(*t*))<*S*(*t*) then

 Robot hard stop (unconditional), $T_{e}^{remain}\left(t\right)$ unchanged

 end if

 end for

 SCHEDULING FEEDBACK: IDQN Task Assignment

 Construct system state *s*_*t*_ from (task states, executor states, env. states)

 Execute IDQN sequential decision:

 for each *e*∈*E*_idle_(*t*), ordered by descending idle duration $\pi_{e}^{dec}\left(t\right)$ (ties broken by ascending executor index):

 Compute feasible action set (applying constraint-based action masking)

 Compute Top-*K* candidates from Q-values;

 execute DT N-step forward simulation;

 select action with highest $Score_{a_{k}}$

 Assign task $J_{a_{e}\left(t\right)}$ to executor *e* (or idle-wait if *a*_*e*_(*t*)=0)

 end for

 Update system state: executor states, remaining loads, material inventory, task completion status;

 update AoI timestamps for executors whose DT received a new state packet in this time step.

 end while

 Output: Final Makespan $C_{\max}=\max\;t_{j}^{e},j\in J$

Three cross-layer coupling paths exist in the above algorithm. First, the AoI values computed by the Perception Layer are directly passed to the Decision Layer’s forward reachable set radius and Gaussian covariance: a lower AoI makes the reachable set more compact, simultaneously reducing the rate of false-positive collision triggers and unnecessary robot pauses. Second, the edge resource feasibility check performed by the Prediction Layer generates a constraint signal that is fed back to the action mask of the scheduling layer’s IDQN: when edge server capacity is saturated, the scheduler is forced to defer the assignment of shared-workspace tasks, preventing edge resource overload. Third, the robot safety pause signal freezes the remaining task time of the affected executor, the system state is updated accordingly, and this prompts the IDQN to reactively re-optimize task assignments for other executors. These three coupling paths are the precise mechanism underlying the super-additive performance gains observed in the ablation study (Table 2).

#### Dynamic task scheduling algorithm based on deep reinforcement learning

The task execution duration of human executors is unpredictable due to temporal uncertainty. Traditional optimization methods based on precise models are difficult to effectively solve such dynamic stochastic problems. To this end, this section introduces Deep Q-Network (DQN)^16^ to construct a real-time dynamic scheduling algorithm. Unlike traditional heuristic methods that generate static schedules before execution, the DQN agent assigns tasks to idle executors based on real-time system states during task execution, effectively addressing the uncertainty in human execution time.

The HRC task scheduling problem is modeled as a Markov Decision Process (MDP), defined as a quadruple (S,A,P,R).

##### State space S

As shown in Table S10, the system state *s*_*t*_ at time *t* consists of task state feature vector f^*task*^, executor state feature vector f^*exec*^, and environment state feature vector f^*env*^.

##### Action space A

At each decision time *t*, the agent needs to assign tasks to all idle executors. Define action *a*_*t*_ as $a_{t}=\left\{\left(e,j\right)|e\in E^{idle}\left(t\right),j\in J^{available}\left(t\right)\cup \left\{\emptyset \right\}\right\}$, where $E^{idle}\left(t\right)=\left\{e\in E|\mu_{e}\left(t\right)=0\right\}$ represents the set of idle executors; $J^{available}\left(t\right)$ represents the set of assignable tasks (predecessors completed and not yet assigned); ∅ represents no task assignment (executor continues to wait). For each idle executor e, its action *a*_*e*_(*t*) is $a_{e}\left(t\right)\in \left\{0,1,2,\ldots ,|J^{available}\left(t\right)|\right\}$. Where *a*_*e*_(*t*) = 0 represents no task assignment, and *a*_*e*_(*t*)=*j* represents assigning task *J*_*j*_. To improve the learning efficiency of the agent, we introduce the constraint-based action masking,^18^ which masks infeasible actions based on the constraints described above before the agent makes decisions. Masking infeasible actions ensures that the agent only explores within the feasible action space.

##### State transition P

Given current state *s*_*t*_ and action *a*_*t*_, the system state transitions to *s*_*t*+1_ according to the following rules: for assigned tasks, update their status to executing and record the start time; for tasks being executed, if $t\geq t_{j}^{e}$, mark as completed. Correspondingly, update executor states, remaining loads, and various resource states based on real-time task assignment situations; if collision probability *P*_*collision*_(*t*)≥*θ*_*safe*_ is detected, the collaborative robot pauses its action, and the task remaining time $T_{e}^{remain}\left(t\right)$ remains unchanged until the risk is eliminated. The high-priority executor set *E*_*high*_(*t*) is updated synchronously at each time step, defined as the set of all human executors currently performing shared-workspace tasks: *E*_*high*_(*t*) = {*e* ∈ *H*|*ρ*_*j*(*e*,*t*)_ = 1,*μ*_*e*_(*t*) = 1}, where *j*(*e*,*t*) denotes the task currently being executed by executor *e*, and *ρ*_*j*_ denotes the collision risk level of task *j*. This set is recomputed at each time step, allowing executors to enter or exit the high-priority state in real time as task assignments change dynamically.

##### Reward function *R*

The optimization objective of the IDQN scheduling layer is to minimize the completion time (Makespan). To this end, the reward function adopts a per-step time penalty that accumulates over the episode duration. The exact expression is $r_{t}=r_{t}^{time}=-\lambda_{time}\cdot \Delta t$, where *r*_*t*_ is the reward received at time step *t*, Δ*t* is the duration of the current time step (in seconds), and *λ*_*time*_ is the time penalty coefficient. The cumulative reward for an episode of duration *T* is $R=\sum_{t=0}^{T}\gamma^{t}\cdot r_{t}=-\lambda_{time}\cdot \sum_{t=0}^{T}\gamma^{t}\cdot \Delta t$, where *γ* is the discount factor. Minimizing the negative of this cumulative reward is equivalent to minimizing the discounted sum of time steps, which incentivizes the agent to complete all tasks as quickly as possible by making efficient task assignment decisions at every step.

In the proposed framework, the objectives of safety and efficiency are handled by entirely independent mechanisms and are not merged into a single weighted reward. The reward function above contains only the time penalty term; no distance-based safety penalty term appears, and there is therefore no need to specify or tune a weighting coefficient between efficiency and safety. Safety is guaranteed by the decision layer of the PPD framework as a model-based hard constraint: whenever the collision probability *P*_*collision*_(*t*), computed from the probabilistic occupancy grid, satisfies *P*_*collision*_(*t*)≥*θ*_*safe*_, the collaborative robot immediately and unconditionally pauses its action, regardless of the action selected by the RL policy. Consequently, the IDQN agent learns to schedule tasks efficiently within an environment in which safety violations are already prevented by the hard constraint layer; the time-penalty reward drives it to reduce unnecessary robot pauses through better task sequencing, rather than trading safety for speed.

This decoupled design is superior to a weighted multi-objective reward formulation for two reasons. First, a weighted formulation requires a scalar coefficient to specify the relative importance of safety versus efficiency—a scalar that must be tuned and whose meaning shifts as the distribution of distance values in the training scenarios changes. Second, and more critically, a weighted formulation provides only soft suppression of safety violations. In principle, if the efficiency gain is sufficiently large, the agent may trade safety for speed. The hard-constraint approach adopted in this paper provides an unconditional guarantee that is independent of reward shaping, making the system’s safety properties provably unaffected by changes to the reward signal.

#### Complexity analysis of state space and scalability discussion

The state vector *s*_*t*_ is composed of three feature blocks: (1) task state features, with dimensionality 7*M*, where *M* is the number of subtasks; (2) executor state features, with dimensionality 4*E*, where *E* = 2*N*+*K* is the total number of executors (*N* HRC groups, *K* AMRs); and (3) environment state features, with fixed dimensionality *d*_*env*_ = 3 (remaining edge server capacity, remaining communication resources, and current time). Therefore, the total state dimensionality is |*s*_*t*_| = 7*M* + 4*E* + 3 = 7*M* + 4(2*N* + *K*) + 3.

For the largest test scenario T15 (*M* = 50, *N* = 5, *K* = 3, *E* = 31): |*s*_*T*15_| = 350 + 124 + 3 = 477. This represents linear growth with respect to *M* and *E*, in sharp contrast to the exponential growth $O\left({\left|S_{single}\right|}^{E}\right)$ of the state space in joint-state MARL approaches such as QMIX^20^ and MAPPO.^21^ The key design decision that enables this tractability is: our agent observes a global but structured state, which, instead of enumerating all joint configurations of all agents, concatenates the same information into a feature vector at a cost of *O*(*M*+*E*). The action space per decision step is $\left|A\right|=\left(\left|J^{available}\left(t\right)\right|+1\right)\times \left|E^{idle}\left(t\right)\right|$, with an upper bound of (*M* + 1) × *E*. IDQN employs a fully-connected Dueling DQN with two hidden layers of size 256. Given state dimensionality *d*_*s*_ = 7*M* + 4*E* + 3, the network forward-pass complexity is $O\left(d_{s}\cdot 256+256^{2}+256\cdot \left|A\right|\right)=O\left(d_{s}+\left|A\right|\right)$, both of which are polynomial in *M* and *E*. The sequential decision mechanism further reduces the effective action space from the joint space of (*M* + 1)^*E*^ to *E* sequential decisions, each of scale (*M* + 1). When the scale of executors and tasks expands, the algorithm can be adapted to larger-scale systems by extending the number of state space dimensions, although the real-time decision-making efficiency of the algorithm will be slightly affected. When the real-time decision-making efficiency of the algorithm no longer meets requirements, a possible extension direction worth considering is to partition the workshop into spatial zones and train meta-agents to coordinate zone-level agents.

In addition, the IDQN policy is trained in a simulation environment where human execution times follow a uniform distribution, and can be transferred to a physical deployment scenario without retraining. In a real workshop, workers may exhibit execution time characteristics that deviate to some extent from the training distribution due to the accumulation of fatigue, differences in individual skill levels, and changes in task proficiency. Two intrinsic design properties of IDQN help mitigate this train-deploy distribution gap: first, the policy is trained under a worst-case bounded-support distribution and is theoretically robust to any distribution sharing the same support upper bound; second, the policy takes real-time remaining task duration rather than *a priori* execution time estimates as its decision input, enabling it to adapt to actual execution progress under any distributional assumption.For deployment scenarios where workers’ execution characteristics deviate systematically from the training distribution, lightweight fine-tuning using a small number of real production episodes (e.g., expanding the state space dimensionality) can recalibrate the policy without requiring full retraining.

#### Network architecture and action decision mechanism

The Dueling DQN architecture^17^ is adopted, which decomposes the Q-value $Q\left(s,a;\theta \right)=V\left(s;\theta_{V}\right)+\left(A\left(s,a;\theta_{A}\right)-\sum_{a^{\prime }}A\left(s,a^{\prime };\theta_{A}\right)/\left|A\right|\right)$ into state value function *V*(*s*) and advantage function *A*(*s*,*a*), where *V*(*s*;*θ*_*V*_) represents the overall value of state *s*, independent of specific actions; *A*(*s*,*a*;*θ*_*A*_) represents the advantage of selecting action a in state s relative to the average action. Dueling DQN can more effectively evaluate state values, especially performing better in situations where action selection has less impact on outcomes.

The shared feature extraction backbone consists of three fully connected layers, with neuron counts set to 512, 256, and 128 respectively in the simulation experiments, each followed by a ReLU activation function. The output of the third shared layer is fed into two separate streams: the value stream maps the 128-dimensional shared representation to a scalar state value *V*(*s*) via a single linear layer (128 to 1); the advantage stream maps the same representation to a per-action advantage value vector *A*(*s*,*a*) via a single linear layer (128 to |*A*|). The two streams are combined according to formula $Q\left(s,a;\theta \right)=V\left(s;\theta_{V}\right)+\left(A\left(s,a;\theta_{A}\right)-\sum_{a^{\prime }}A\left(s,a^{\prime };\theta_{A}\right)/\left|A\right|\right)$ to obtain the final Q-value estimate.

Since multiple idle executors may need task assignments at each decision time, this paper introduces a sequential decision strategy based on idle duration. Define the decision priority $\pi_{e}^{dec}\left(t\right)=t-t_{e}^{last}$ of idle executor $e\in E^{idle}\left(t\right)$ as its cumulative idle duration, where $t_{e}^{last}$ is the time when executor *e* last finished task execution. The agent assigns tasks to idle executors in descending order of priority: first calculate the feasible action set, then select the action with the highest Q-value. When multiple executors share an identical idle duration, the one with the smaller executor index is processed first.

To fully leverage the iterative optimization capability of the DT, the action selection step is expanded into a DT-assisted forward simulation evaluation process.^14^ The agent first selects the Top-*K* candidate actions from the feasible action set ranked by Q-value. For each candidate action *a*_*k*_, the DT performs an *N*-step forward simulation in the virtual environment without modifying the physical environment state, recording the simulated collision probability trajectory {*P*_*collision*_(*t*+1),...,*P*_*collision*_(*t* + *N*)} and estimated task progress, and computing a composite score: $Score_{a_{k}}=\alpha \cdot Q\left(s_{t},a_{k}\right)+\left(1-\alpha \right)\cdot \left(-\sum_{n=1}^{N}P_{collision}\left(t+n\right)\right)$, where *α* balances the IDQN Q-value estimate (representing the learned long-term scheduling quality) against the short-term collision risk from DT simulation (representing the immediate safety consequence of the candidate action). The candidate action with the highest composite score is selected for execution. This process runs only during the inference phase; the IDQN training process remains unchanged, and no retraining of the Q-network is required.

Prioritizing task assignment to long-idle executors helps improve overall resource utilization. The theoretical basis of this sorting criterion is rooted in parallel machine scheduling theory: in a parallel-executor system with makespan minimization as the objective, a necessary condition for optimality is that load remains balanced across executors—that is, no executor is persistently underutilized while others are overloaded. Prioritizing task assignment to the longest-idle executor directly counteracts workload imbalance: it identifies the executor that has deviated furthest from the ideal balanced state and prioritizes returning it to active status, which monotonically reduces the maximum idle time in the executor pool and thereby tightens the lower bound of makespan. This mechanism is conceptually aligned with the Longest Idle Time First scheduling principle studied in parallel machine scheduling, which has been shown to improve load balancing and reduce makespan under stochastic processing time settings.^32^ Additionally, to ensure algorithm usability in practical systems, we impose constraints on the agent’s single decision time. Define the maximum allowed time for a single decision as $T_{\max}^{dec}=1$ s (real time), and the agent must complete action selection for all idle executors within this time: $T^{dec}\left(t\right)=T^{mask}\left(t\right)+T^{infer}\left(t\right)+T^{DT-sim}\left(t\right)\leq T_{\max}^{dec}$, where *T*^*mask*^(*t*) is the action mask computation time, *T*^*infer*^(*t*) is the network inference time, *T*^*DT*-*sim*^(*t*) is the forward simulation time of the Digital Twin for Top-*K* candidate actions. If the remaining single decision time is insufficient to complete the action decision for an idle executor e, that executor maintains idle status until the next decision begins, and its cumulative idle duration is updated.

As the number of executors *E* varies across different scenarios, the state vector dimensionality changes accordingly. To accommodate this variation across multiple test scenarios without retraining, we define the fixed input dimensionality of the neural network using the maximum scenario scale in the subsequent simulation experiments, and apply zero-padding to positions corresponding to missing executors and unscheduled tasks in the state vectors of smaller scenarios. This zero-padding strategy maintains a consistent network architecture across all scenarios, with padded positions masked out via the action masking mechanism to ensure that absent entities do not affect Q-value estimation.

#### Dynamic priority experience replay mechanism

Experience Replay breaks the temporal correlation between samples by storing and reusing historical experiences. Traditional uniform sampling strategies ignore the differences in contributions of different experiences to learning. This paper introduces a dynamic priority-based experience replay mechanism,^19^ which adaptively adjusts sampling priorities based on the TD error and training stage of experiences. The sampling priority *p*_*i*_=∣*δ*_*i*_∣+*ϵ* of experience *i* is calculated based on its TD error $\delta_{i}=r_{i}+\gamma \max_{a^{\prime }}Q\left({s_{i}}^{\prime },a^{\prime };\theta^{-}\right)-Q\left(s_{i},a_{i};\theta \right)$. Where *ϵ* is a small constant to prevent zero priority (e.g., *ϵ*=10^-6^), *r*_*i*_ is the immediate reward, *γ* is the discount factor, *θ*^-^ is the target network parameters, and *θ* is the current network parameters. TD error reflects the “prediction deviation” of the current Q-network for that experience. Larger TD error means the experience contains information that the current network has not yet fully learned, and prioritizing sampling of such experiences helps accelerate network convergence. To make the priority threshold adaptive to the training process, define the average priority of the experience pool as $\overset{-}{p}\left(t\right)={\left|D\right|}^{-1}\cdot \sum_{i=1}^{\left|D\right|}p_{i}$, and dynamically adjust the priority $p_{i}^{dyn}=p_{i}\cdot \left(1+\lambda_{dyn}\cdot \left(p_{i}-\overset{-}{p}\left(t\right)\right)/\overset{-}{p}\left(t\right)\right)$, where *λ*_*dyn*_∈[0,1] controls the dynamic adjustment magnitude. When $p_{i}>\overset{-}{p}$, the adjustment factor $p_{i}-\overset{-}{p}/\overset{-}{p}>0$, and the priority of high-value experiences is further amplified; when $p_{i}<\overset{-}{p}$, the adjustment factor $p_{i}-\overset{-}{p}/\overset{-}{p}<0$, and the priority of low-value experiences is relatively compressed. In the early training stage, the overall TD error is large and $\overset{-}{p}$ is high; in the later training stage, the network gradually converges and $\overset{-}{p}$ decreases. The dynamic adjustment mechanism enables the priority threshold to adapt to the training process, always focusing on the experiences with the highest learning value at the current stage. Based on dynamic priority, the sampling probability of experience *i* is $P\left(i\right)={\left(p_{i}^{dyn}\right)}^{\alpha }/\left(\sum_{k}{\left(p_{k}^{dyn}\right)}^{\alpha }\right)$, where *α*∈[0,1] controls the balance between priority sampling and uniform sampling. Based on *α*, the algorithm can both retain the efficiency advantage of priority sampling and maintain certain sampling diversity to avoid overfitting.

Since priority sampling changes the sampling distribution of experiences, making it deviate from the true data distribution, it may cause bias in Q-value estimation. To correct this bias, importance sampling weights $w_{i}={\left(\left|D\right|P\left(i\right)\right)}^{-\beta }$ are introduced, where *β*=*β*_0_+(1-*β*_0_)·*episode*/*N*_*episodes*_∈[0,1] is the bias correction exponent. To ensure training stability, weights are usually normalized: ${\tilde{w}}_{i}=w_{i}/\max_{j}w_{j}$. In the early training stage, network parameters change dramatically, and the importance of bias correction is relatively low, so a smaller *β* value can be used; as training progresses, *β* is gradually increased to 1 to achieve complete correction. This annealing strategy allows more aggressive priority sampling in the early training stage to accelerate learning, while ensuring unbiasedness of Q-value estimation in the later training stage.

#### Loss function and training process

Combining importance sampling weights, the loss function of DQN is defined as $L\left(\theta \right)=E_{\left(s,a,r,s^{\prime }\right)∼D}\left[w_{i}{\left(y_{i}-Q\left(s_{i},a_{i};\theta \right)\right)}^{2}\right]$, where the target Q-value $y_{i}=r_{i}+\gamma \cdot \max_{a^{\prime }}Q\left({s_{i}}^{\prime },a^{\prime };\theta^{-}\right)$. The target network parameters *θ*^-^ gradually track the main network through soft update: *θ*^-^←*τθ*+(1-*τ*)*θ*^-^, where *τ*∈(0,1) is the soft update coefficient.

The complete training process of the algorithm is shown in Algorithm 2. Each training episode simulates a complete task execution process, and the agent learns the optimal task allocation strategy in the simulated environment. During training, an *ε*-greedy exploration strategy is adopted: the exploration rate *ε* is initialized to 1.0 and gradually decays to a minimum of 0.05 over training iterations, after which it remains fixed. This scheme concentrates exploration in the early stages of training while ensuring sufficient exploitation during the convergence phase.

Algorithm 2 **DQN-based dynamic task scheduling training algorithm**

Input: Task set J, Executor set E, Number of training episodes *N*_*episodes*_

Randomly initialize Q-network parameters *θ*

Initialize target network parameters *θ*^-^

Initialize experience pool D

Initialize exploration rate *ε*

for *episode*=1 to *N*_*episodes*_ do:

 *t*=0:

 Initialize all task states: $\eta_{j}\left(0\right)=0,\forall J_{j}\in J$

 Initialize all executor states: $\mu_{e}\left(0\right)=0,L_{e}\left(t\right)=L_{e}^{\max},\forall e\in E$

 Initialize edge server resources: $C_{v}^{remain}\left(0\right)=C_{v}^{\max},\forall v\in V^{edge}$

 Initialize communication resources: *B*^*remain*^(0) = *B*_*total*_

 Obtain initial state

Task execution simulation loop: while $\exists J_{j}\in J:\eta_{j}\left(t\right)\neq 2$ do:  # Uncompleted tasks exist

 Obtain idle executors $E^{idle}\left(t\right)=\left\{e\in E|\mu_{e}\left(t\right)=0\right\}$ Assign tasks to idle executors: if $E^{idle}\left(t\right)\neq \emptyset$ then

 Sort by decision priority: $E^{sorted}\left(t\right)=\text{Sort}\left(E^{idle}\left(t\right),\pi_{e}^{dec}\left(t\right),\text{desc}\right)$ (ties broken by ascending executor index)

 for $e\in E^{\text{sorted}}\left(t\right)$ do

 Compute feasible action set $A_{e}^{valid}\left(t\right)$

 ε-greedy strategy to select action:

 if random()<*ε* then

 *a*_*e*_(*t*)= RandomChoice($A_{e}^{valid}\left(t\right)$)

 else

 *a*_*e*_(*t*)= arg $\max_{a\in A_{e}^{valid}\left(t\right)}Q\left(s_{t},a;\theta \right)$

 end if

 Execute task assignment action:

 if *a*_*e*_(*t*)≠∅ then

 *j* = *a*_*e*_(*t*)*η*_*j*_(0)= 1, $t_{j}^{s}=t$ # Task starts execution

 *μ*_*e*_(*t*)=1 # Executor state update

 $T_{e}^{remain}\left(t\right)=T_{j,e}$ # Set remaining execution time

 Update resource states

 end if

 end for

 end if

# Environment step

 *t* = *t* + Δ*t*

Update task execution progress

Update executor remaining loads

Update resource states

Collision detection and response:

 for $e\in E^{high}\left(t\right)$ do # Iterate through high-priority executors

 Compute collision probability *P*_*collision*,*e*_(*t*)

 if *P*_*collision*,*e*_(*t*)≥*θ*_*safe*_ then

 Collaborative robot pauses, $T_{e}^{remain}\left(t\right)$ remains unchanged

 $E^{high}\left(t\right)$ = $E^{high}\left(t\right)\cup \left\{e\right\}$

 end if

 end for

 Compute reward

 Update state

 Store experience

 Network update

 end while

 Record Makespan

 Exploration rate *ε* decay

 Bias correction exponent *β* annealing

end for

Output: Trained Q-network parameters, Optimal Makespan

### Quantification and statistical analysis

All quantitative results reported in this paper are derived from simulation experiments conducted in a Python-based discrete-event simulation environment. No biological or clinical data are involved. The statistical details of all experiments are reported in the figure legends and result tables throughout the main text.

The primary performance metric is Makespan (total task completion time, in seconds). Secondary metrics include collision response trigger count, SSM hard-stop trigger count and duration, AoI of high-priority executors, DT state transmission latency, average task waiting time, algorithm convergence episodes, post-convergence reward standard deviation, optimal solution acquisition rate, and single-decision inference time.

For each experimental condition, each test scenario is independently run n = 20 times with human execution times resampled independently from U(30,60) seconds in each run, while scenario structural parameters (task graph topology, executor count, task type proportions) are held fixed using pre-assigned random seeds. All reported values represent the mean across these 20 independent runs. Dispersion is reported as standard deviation (SD) throughout. No hypothesis testing or *p*-value thresholds are applied; performance differences are characterized by percentage changes relative to the designated baseline or control group.

Scenario structural parameters and neural network weights are initialized using fixed global random seeds to ensure reproducibility of the scenario set and training process across runs. Human execution times within each run are stochastic and not fixed by seed, ensuring that the 20 runs per scenario constitute genuinely independent replications under identical structural conditions.

All simulation experiments and data analyses were performed using Python 3.10. Neural network training was implemented using PyTorch. No additional statistical software packages were used.
