# Supplementary material for: Balancing safety and efficiency in large-scale HRC: A dynamic scheduling method confronting temporal and spatial uncertainty of human workers
Source: iScience. 2026 Aug 7;29(8):117095. doi: 10.1016/j.isci.2026.117095 (PMC13476530; doi:10.1016/j.isci.2026.117095)
Supplement: Supplementary file 1 [file mmc1.pdf]

iScience, Volume *29*

Supplemental information

Balancing safety and efficiency in large-scale HRC: A dynamic scheduling method confronting temporal and spatial uncertainty of human workers

Houxue Xia, Zhenyu Sun, and Huagang Tong

Figure S1. IDQN Converges Stably within 6,000 Episodes under Complex Constraint Conditions. Three training phases are annotated: exploration (episodes 0–2,000), rapid learning (episodes 2,000–6,000), and stable convergence (episodes 6,000–10,000).

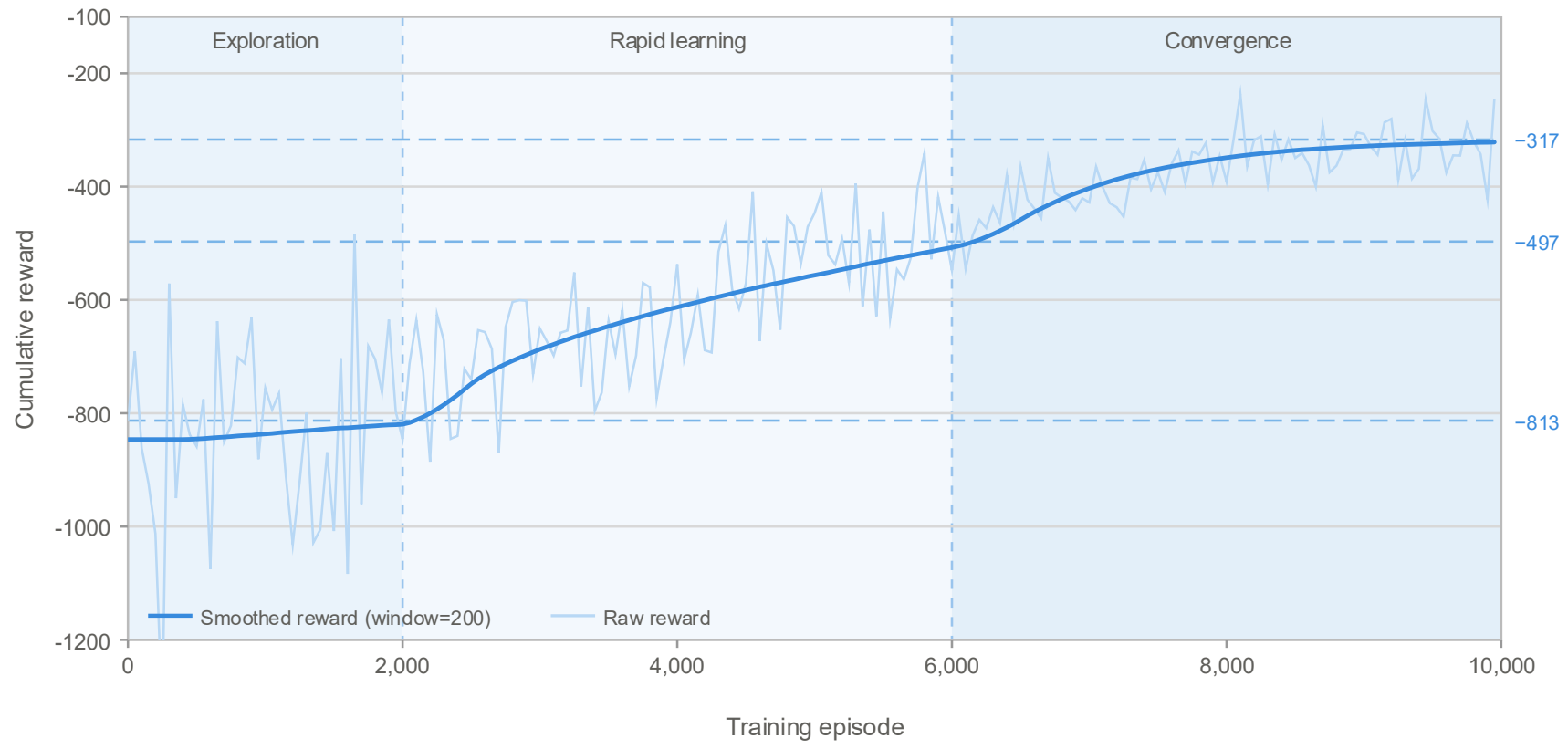

Figure S2. IDQN Produces Balanced Load Distribution across Executors while Satisfying All Precedence Constraints. Gantt chart shows a representative single run for scenario T5 (30 tasks, 13 executors, 7 shared workspace tasks). Task bars are color-coded by executor; shared workspace tasks are highlighted with red borders. Predecessor dependency chains ($J12 \rightarrow J25$, $J28 \rightarrow J15 \rightarrow J6$, $J17 \rightarrow J23$) are respected throughout. This figure presents a single deterministic scheduling outcome for illustration purposes; aggregate statistics across 20 runs are reported in Table 1 in the main text.

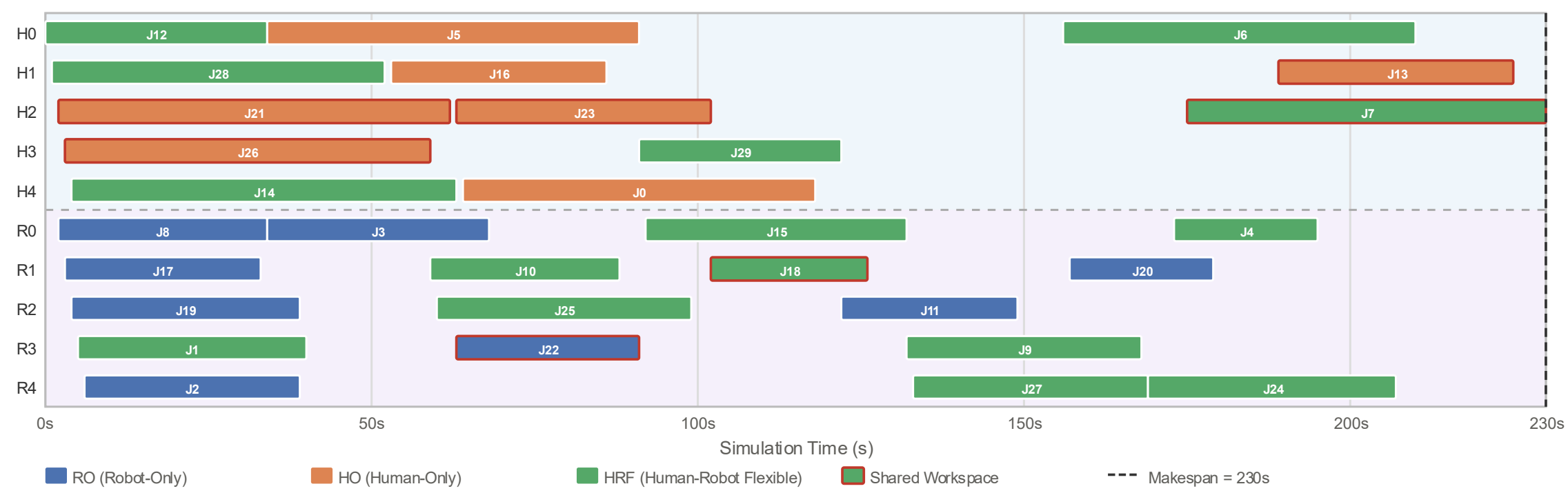

Figure S3. Elastic Deployment Achieves the Lowest Task Waiting Time and Makespan among All Three Deployment Strategies. Bar charts compare three DT deployment strategies in scenario T9 (39 tasks, 21 executors). (A) Average total latency (link + server) for high-priority and low-priority executors. (B) Task delay count caused by edge resource exhaustion and average task waiting time. All bars represent means across n = 20 independent runs. Experimental group: elastic deployment (high-priority to edge, low-priority to cloud); Control group 1: static cloud deployment; Control group 2: undifferentiated edge deployment.

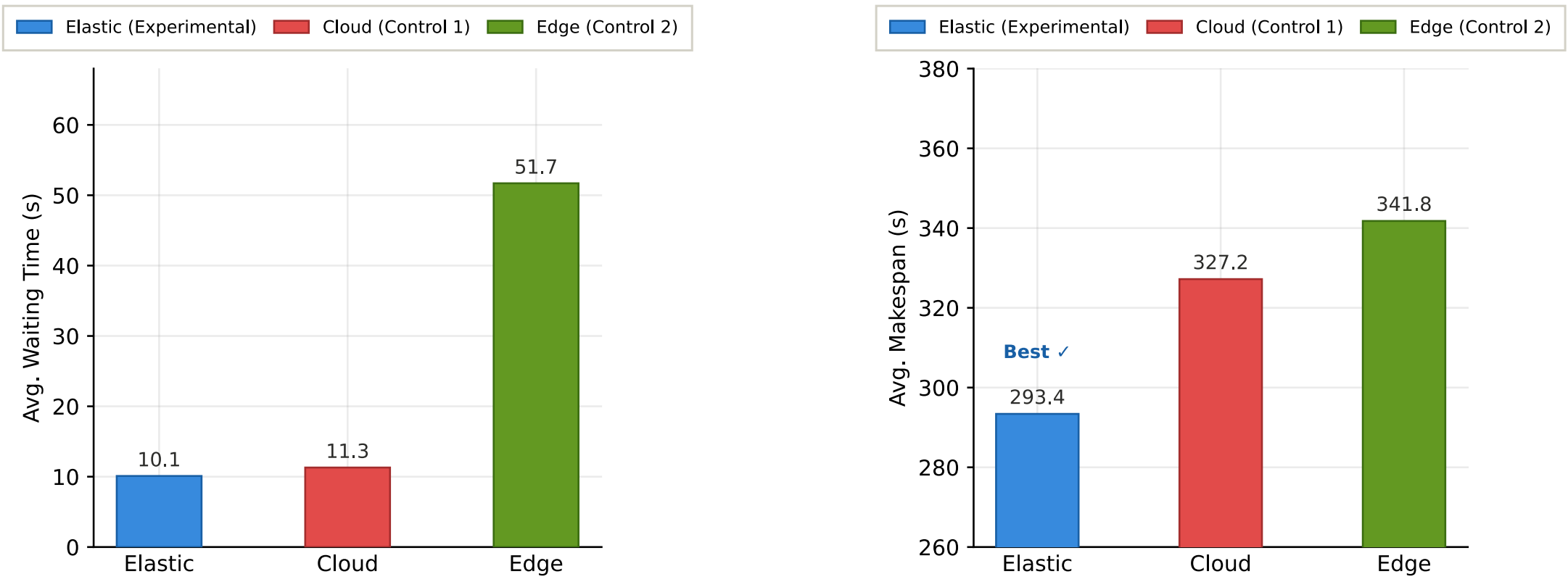

(A) Resource Constraint Impact **(B) Overall Scheduling Efficiency**

Figure S4. Full PPD Configuration Dominates All Ablation Groups across Safety and Efficiency Dimensions. Radar chart shows normalized performance scores across five metrics for four configurations: experimental group (full PPD), ablation group A (no AoI differentiation), ablation group B (no DT elastic deployment), and baseline group (decision layer only). Scores are normalized so that 1.0 = experimental group and 0 = baseline group on each axis; larger polygon area indicates better overall performance. Each data point represents the mean across n = 20 independent runs per scenario, averaged over all 15 test scenarios (300 runs per configuration in total). No within-group error is shown; metric-level statistics are reported in Table 2 in the main text.

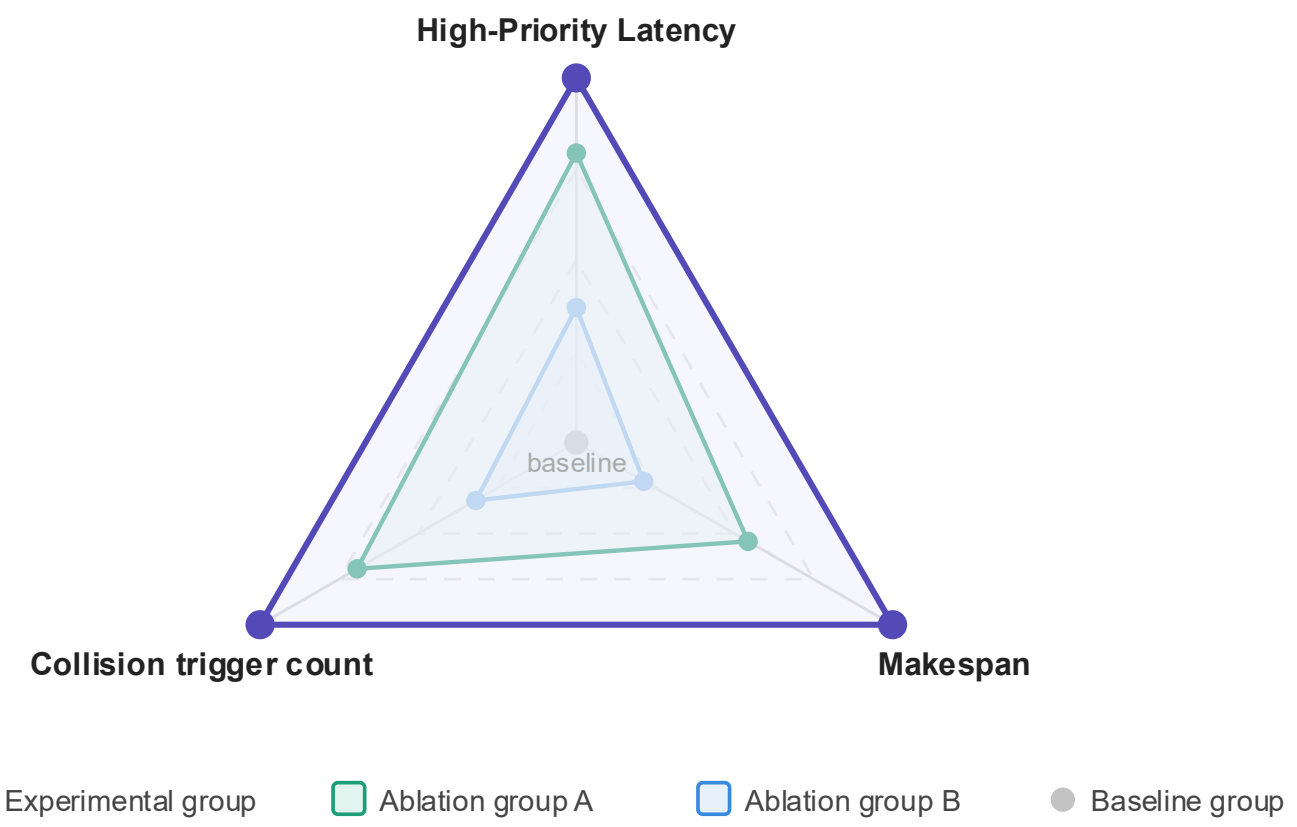

Figure S5. IDQN Converges 27.7% Faster than Standard DQN with Lower Post-Convergence Reward Variance.

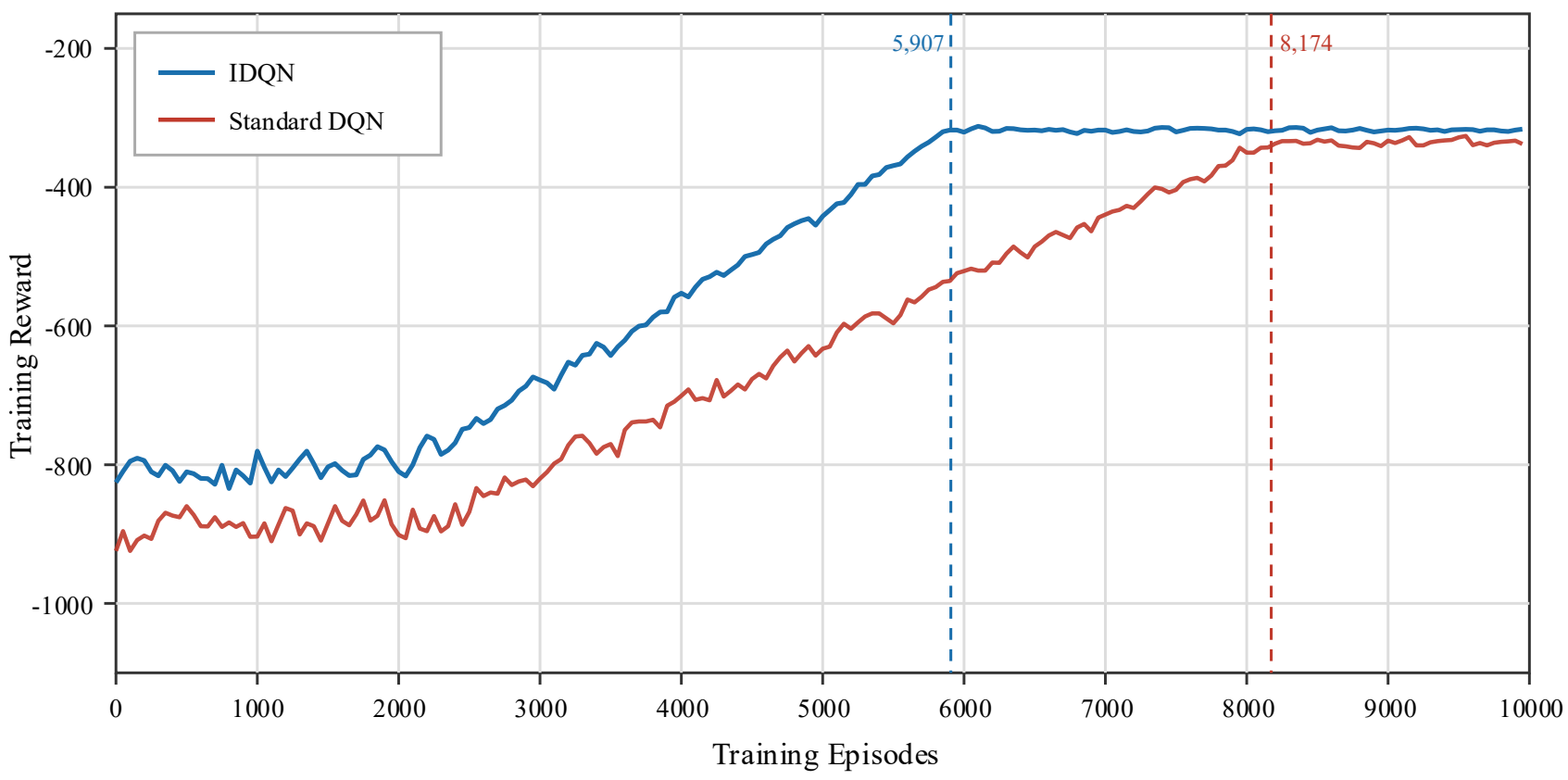

Figure S6. IDQN Achieves the Lowest Decision Latency among Three Algorithms. Line chart shows average single-decision inference time (ms) for IDQN, QMIX, and MAPPO across 15 test

scenarios (E = 8 to 31 executors). Each data point represents the mean inference time across n = 20 independent runs for that scenario. The horizontal dashed line indicates the real-time constraint ceiling (1,000 ms). See also Table S6 for maximum single-decision times.

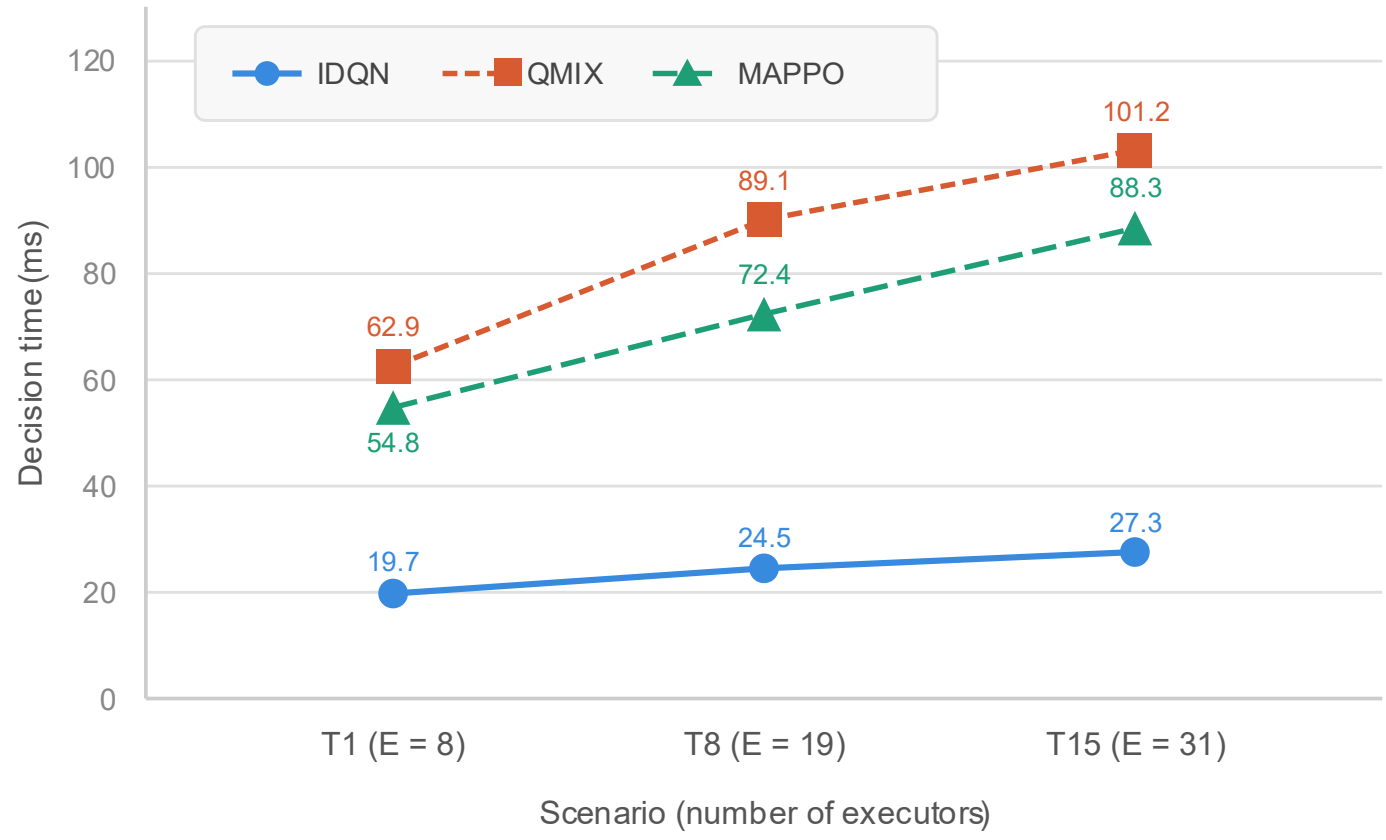

Figure S7. Action Masking Most Strongly Impairs Training Efficiency when Removed, while Decision Priority Most Strongly Impairs Dynamic Robustness.

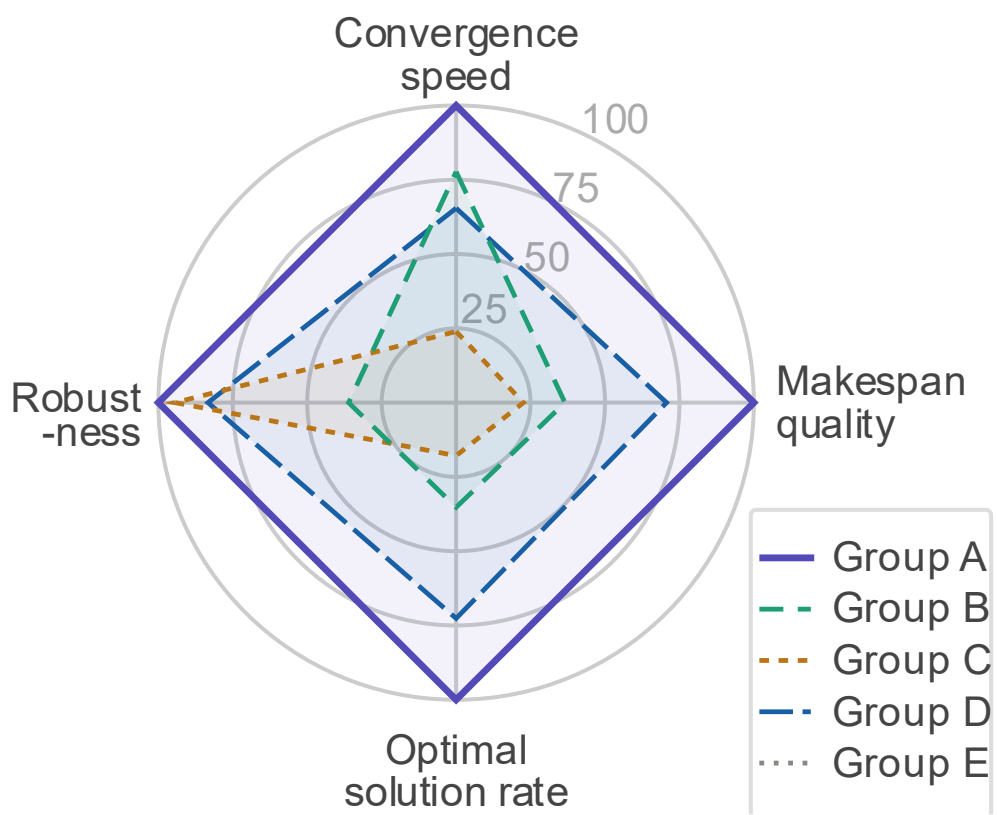

(A) Multi-dimensional performance comparison of ablation groups. Radar chart showing normalized multi-dimensional performance scores for five ablation configurations: Group A (full IDQN), Group B (no decision priority), Group C (no action masking), Group D (no DT forward simulation), Group E (standard DQN, all three mechanisms removed). Normalization: 1.0 = Group A, 0 = Group E on each axis.

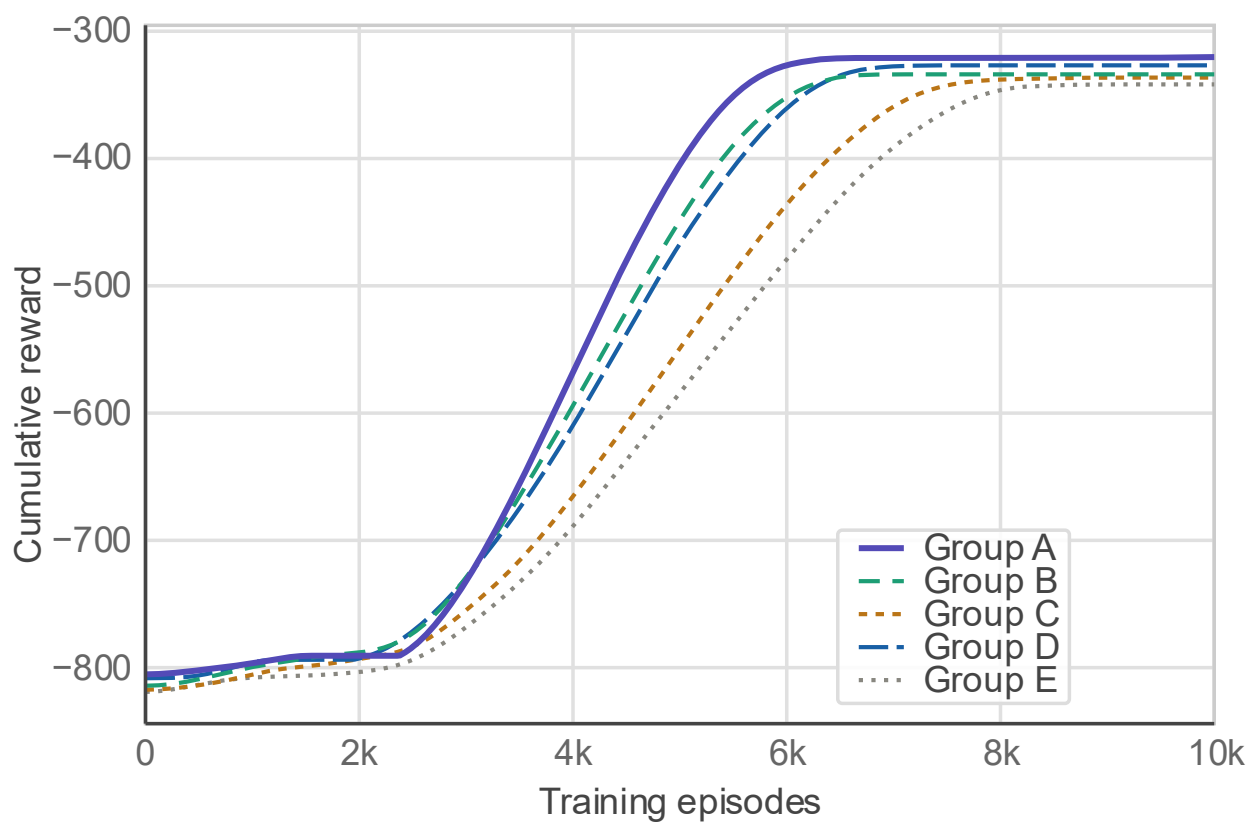

(B) Training convergence curves of ablation groups. All scheduling performance metrics represent means across n = 20 independent runs per scenario, averaged over all 15 test

scenarios. See Table S7 for full statistics.

Figure S8. Schematic layout of HRC workshop, related to STAR Methods.

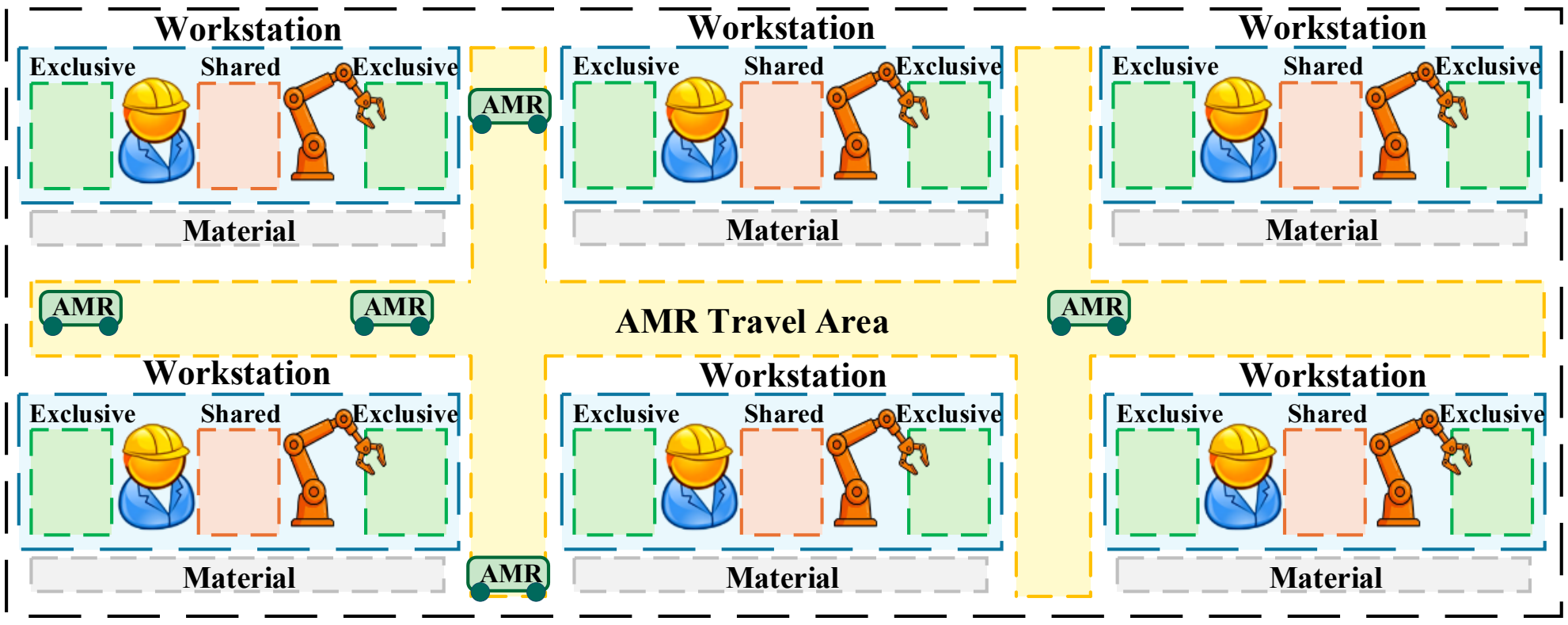

Figure S9. Differentiated link resource allocation, related to STAR Methods.

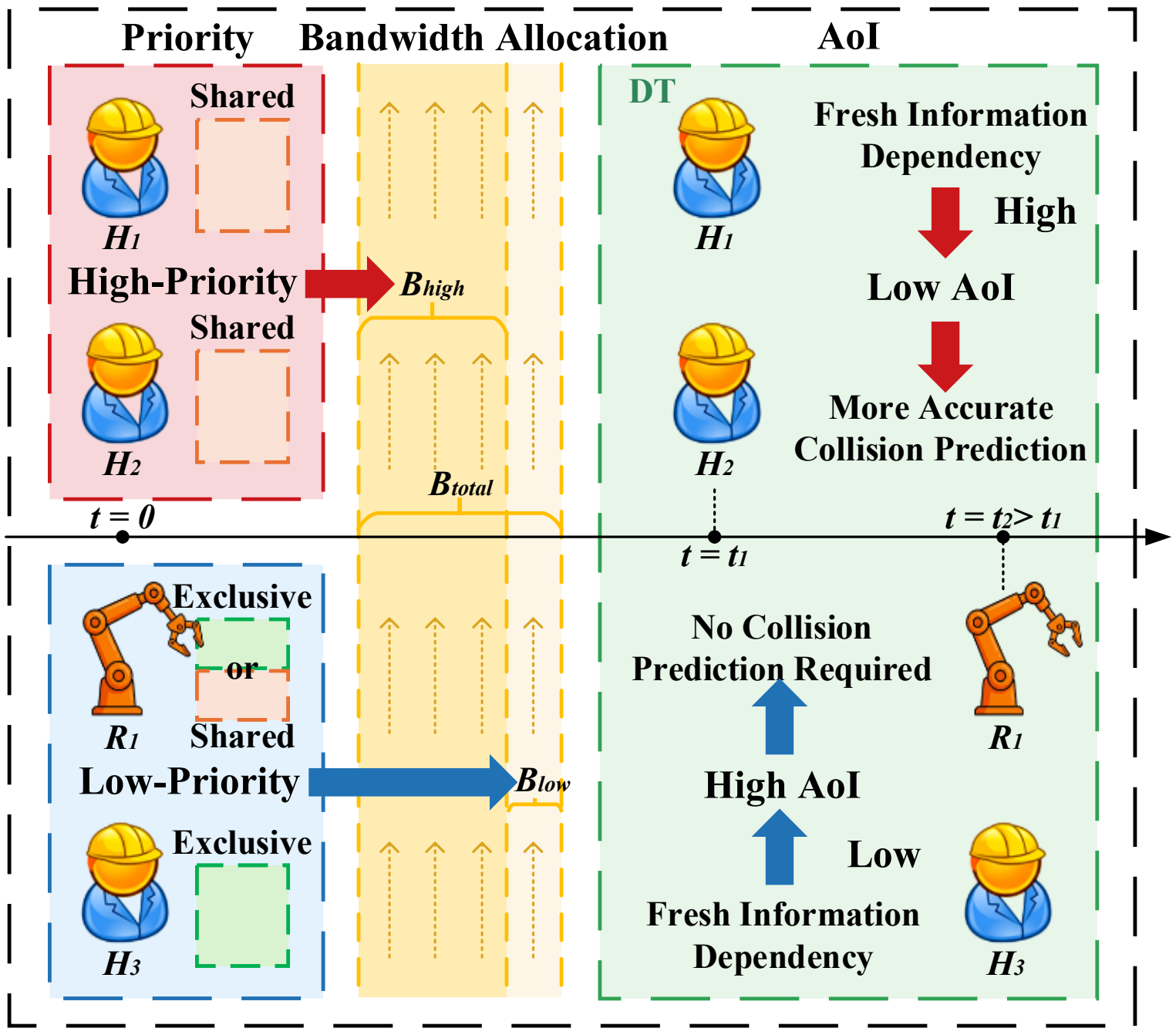

Table S1. Key Parameter Settings.

Parameter	Symbol	Value	Description
Number of training episodes	$N_{episodes}$	10000	Ensure sufficient policy convergence
Discount factor	γ	0.99	Emphasize long-term returns
Learning rate	η	10^{-4}	Adam optimizer
Batch size	N_{batch}	64	Number of experiences sampled each time
Experience pool capacity	$\mathcal{D}_{\max}$	10^5	Store historical experiences
Priority exponent	α	0.6	Dynamic priority sampling intensity

Parameter	Symbol	Value	Description
Soft update coefficient	τ	0.005	Target network update
Time penalty	λ_{time}	1	Reward function weight
Total communication bandwidth	B_{total}	100 Mbps	Total capacity of workshop wireless network
Minimum bandwidth requirement	B_{min}	2 Mbps	Minimum transmission requirement per executor
High-priority bandwidth proportion	β	0.7	High-risk executors share 70% bandwidth
Number of edge servers	$\|\| \mathcal{V}^{edge} \|\|$	5	Distributed in different areas of workshop
Single server resource capacity	$C_v^{\max}$	4	Upper limit of DT count that can be hosted
Single DT resource requirement	$C^{\min}$	1	Standardized resource unit
Human maximum acceleration	$a_{\max}$	3 m/s²	Physiological limit of human limb movement
Spatial cell edge length	l	0.1 m	3D grid resolution
AoI threshold (high/low)	Δ^{th}	0.5/2.0 s	Shared/exclusive workspace tasks
Collision probability threshold	θ_{safe}	0.3	Risk threshold for triggering robot pause
Baseline position uncertainty	σ_0	0.05 m	Considering sensor measurement errors
Human initial physical load	L_H^{max}	100	All human executors have the same initial physical load
Robot initial load capacity	L_R^{max}	100	All robot executors have the same initial load capacity
Task physical load consumption (human execution)	F_j^H	$\mathrm{U}(5,20)$	Physical load consumption when task J_j is executed by human, uniformly distributed
Task load consumption (robot execution)	F_j^R	$\mathrm{U}(3,15)$	Load consumption when task J_j is executed by robot, uniformly distributed
Human physical effort recovery rate	γ_H	0.5 /s	Physical effort recovered per second by human, same for all humans

Parameter	Symbol	Value	Description
Robot load recovery rate	γ_R	0.8 /s	Load recovered per second by robot, same for all robots
Material consumption per assembly subtask	G_j	$U(1,3)$	Minimum material inventory required for task J_j execution
Material replenishment per AMR task	GA	$U(4,6)$	Material quantity that can be replenished to workstation per AMR task execution
Initial material inventory at workstation	$GS_i(t=0)$	$U(10,20)$	Initial material inventory at HRC workstation i at $t=0$
Robot end-effector equivalent radius	r_R	0.30 m	Cylindrical envelope radius
Human limb cylindrical envelope radius	r_H	0.15 m	Cylindrical envelope radius of human forearm
SSM total system response and braking time	T_{stop}	0 s	Instantaneous robot pausing assumption

Table S2. Comparative Experimental Results of Differentiated Link Resource Allocation Strategy.

Metric Value	experimental group	control group	Reduction (%)
Average High-Priority AoI (s)	0.061	0.178	65.7%
Average Low-Priority AoI (s)	0.317	0.178	not safty critical
Average Collision Response Count	16.1	24.5	34.3%
Average Makespan (s)	257.4	279.9	8.0%
Average Robot Safety Pause Time (s)	17.7	32	44.7%

Table S3. Cross-Scenario Robustness Summary of AoI Differentiated Allocation Strategy.

Scenario	T3	T6	T12
Shared Workspace Task Proportion	35%	52%	58%
Average High-Priority AoI (s) (experimental group)	0.046	0.061	0.074
Average High-Priority AoI (s) (control group)	0.137	0.178	0.201
AoI Reduction (%)	66.4	65.7	63.2
Average Collision Response Count (experimental group)	7.6	16.1	27.9
Average Collision Response Count (control group)	9.7	24.5	43.2
Response Count Reduction (%)	21.6	34.3	35.4
Average Makespan (s) (experimental group)	281.6	257.4	301.6

Average Makespan (s) (control group)	289.0	279.9	331.7
Makespan Reduction (%)	2.6	8.0	9.1

Table S4. Comparative Experimental Results of DT Deployment Strategies.

Metric	Experimental Group	Control Group 1	Control Group 2
Average total latency of high-priority executors (ms)	68.17	234.86	67.10
Average total latency of low-priority executors (ms)	231.79	234.81	66.91
Average task waiting time (s)	10.1	11.3	51.7
Task delay count caused by edge resources	0	0	36.9
Collision response trigger count	8.6	17.7	7.9
Average Makespan (s)	293.4	327.2	341.8
Average robot safety pause time (s)	16.1	37.1	16.9

Table S5. Cross-Scenario Robustness Summary of DT Elastic Deployment Strategy.

Metric	T3	T9	T12
Average total latency of high-priority executors (ms)(experimental group)	47.11	68.17	75.20
Average total latency of high-priority executors (ms) (Control Group 1)	217.78	234.86	249.12
Latency Reduction (%)(experimental group vs. Control Group 1)	78.4	71.0	69.8
Task delay count caused by edge resources(experimental group)	0	0	0
Task delay count caused by edge resources (Control Group 2)	27.6	36.9	43.8
Average Makespan (s) (experimental group)	280.9	293.4	301.6
Average Makespan (s) (Control Group 1)	314.1	327.2	342.9
Average Makespan (s) (Control Group 2)	327.0	341.8	366.6

Table S6. Comparison of IDQN with QMIX and MAPPO.

Evaluation Dimension	Metric	IDQN	QMIX	MAPPO
Scheduling Performance	average Makespan (s)	315.9	321.4	313.7
	makespan standard deviation (s)	17.8	32.1	26.9
	optimal solution acquisition rate (%)	74.1	61.8	62.4
Dynamic Robustness	low-volatility Makespan (s)	307.6	310.3	304.2
	high-volatility Makespan (s)	315.4	317.2	310.3

	performance degradation rate (%)	2.5	2.2	2.0
Computational Efficiency	average single decision time (ms), T1 (E=8)	19.7	62.9	54.8
	average single decision time (ms), T8 (E=19)	24.5	89.1	72.4
	average single decision time (ms), T15 (E=31)	27.3	101.2	88.3
	max single decision time (ms), T15	32.8	118.6	109.2

Note: For the definition of optimal solution acquisition rate, see the note to Table 3. Performance degradation rate = (high-volatility Makespan − low-volatility Makespan) / low-volatility Makespan.

Table S7. Ablation Study Results of IDQN Component Mechanisms.

Mechanism Configuration	Group A	Group B	Group C	Group D	Group E
Convergence Episodes	5914	6432	7681	6717	8237
Post-convergence reward standard deviation	18.2	24.3	27.8	19.3	31.7
Average Makespan (s)	321.0	334.2	337.1	327.1	341.8
Makespan standard deviation (s)	16.3	23.5	22.9	23.4	25.1
Optimal solution acquisition rate (%)	79.3	52.1	44.8	67.8	37.3
Performance degradation rate (%)	3.1	5.4	3.3	3.7	6.7

Note: For the definition of optimal solution acquisition rate, see the note to Table 3. Performance degradation rate = (high-volatility Makespan − low-volatility Makespan) / low-volatility Makespan.

Table S8. IDQN Robustness Validation Results under Skill–Fatigue Coupled Heterogeneity Settings.

Setting	Metric	IDQN	Standard DQN	GA	Rule-based
Group A	average Makespan (s)	315.1	330.8	325.7	335.2
	makespan standard deviation (s)	17.6	24.9	65.1	64.0
Group B	average Makespan (s)	322.8	343.7	345.2	351.3
	makespan standard deviation (s)	21.3	30.8	73.1	69.6
	performance degradation Rate (%)	2.4	3.8	6.0	4.8
Group C	average Makespan (s)	335.6	356.3	379.4	367.8
	makespan standard deviation (s)	26.1	38.7	94.2	78.3
	performance degradation Rate (%)	6.5	7.7	16.5	9.7

Note: Performance Degradation Rate = (heterogeneous Makespan − homogeneous Makespan) / homogeneous Makespan; positive values indicate degradation.

Table S9. Task inherent attributes, related to STAR Methods.

Attribute	Symbol	Definition
Task Type	τ_j	$\tau_j \in \{RO, HO, HRF\}$
Predecessor Subtask Set	P_j	Set of tasks that must be completed before J_j
Spatial Type	σ_j	Whether the execution space involves shared workspace
Robot Execution Duration	T_j^R	See below, deterministic value
Load Requirement	F_j	Physical load required to execute the task
Material Consumption	G_j	Amount of material consumed during task execution

Table S10. State feature vector composition, related to STAR Methods.

Feature	Symbol	Definition
State of subtask J_j	$\eta_j(t)$	0: Unassigned, 1: Executing, 2: Completed
Type of subtask J_j	τ_j	RO/HO/HRF
Spatial type of subtask J_j	σ_j	0: Exclusive workspace, 1: Shared workspace
Predecessor completion status of subtask J_j	$\xi_j(t)$	Whether all predecessor tasks are completed (0/1)
Load requirement of subtask J_j	F_j	Remaining physical load required by executor to complete the task
Material requirement of subtask J_j	G_j	Material inventory required for task execution

Feature	Symbol	Definition
Estimated execution duration of subtask J_j	T_j	Depends on executor type: if executed by robot, T_j is a deterministic value; if executed by human, T_j can only be determined after formal assignment
Type of executor e	$type_e$	H/R/M
State of executor e	$\mu_e(t)$	0: Idle, 1: Executing
Remaining physical load of executor e	$L_e(t)$	Remaining physical load of executor at time t
Remaining task time of executor e	$T_e^{remain}(t)$	If executing, remaining execution time; if idle, equals 0
Remaining capacity of edge server	$C_v^{remain}(t)$	$C_v^{remain}(t) = C_v^{\max} - \sum_{e \in \mathcal{E}} x_{e,v}(t) \cdot C^{\min}$ real-time remaining resource capacity of edge server v
Remaining communication resource	$B^{remain}(t)$	$B_{total} - \sum_{e \in \mathcal{E}^{active}(t)} B_e(t)$, currently available communication bandwidth
Dangerous executor set	$\mathcal{E}^{danger}(t)$	$\{e \in \mathcal{E} \mid P_{collision,e}(t) \geq \theta_{safe}\}$, set of executors whose collision probability exceeds the threshold
Current time	t	Current decision time
